# The Diversity of Volatile Compounds in Australia’s Semi-Desert Genus *Eremophila* (Scrophulariaceae)

**DOI:** 10.3390/plants10040785

**Published:** 2021-04-16

**Authors:** Nicholas J. Sadgrove, Guillermo F. Padilla-González, Alison Green, Moses K. Langat, Eduard Mas-Claret, Dane Lyddiard, Julian Klepp, Sarah V. A.-M. Legendre, Ben W. Greatrex, Graham L. Jones, Iskandar M. Ramli, Olga Leuner, Eloy Fernandez-Cusimamani

**Affiliations:** 1 Jodrell Science Laboratory, Royal Botanic Gardens Kew, Richmond TW9 3DS, UK; f.padilla@kew.org (G.F.P.-G.); a.green2@kew.org (A.G.); m.langat@kew.org (M.K.L.); E.mas-claret@kew.org (E.M.-C.); 2 School of Science and Technology and School of Rural Medicine, University of New England, Armidale, NSW 2351, Australia; dane.lyddiard@une.edu.au (D.L.); julian.klepp@googlemail.com (J.K.); sarah.legendre.pro@gmail.com (S.V.A.-M.L.); bgreatre@une.edu.au (B.W.G.); gjones2@une.edu.au (G.L.J.); iskandarmuda.ssi@gmail.com (I.M.R.); 3 Department of Crop Sciences and Agroforestry, Faculty of Tropical AgriSciences, Czech University of Life Sciences Prague, Kamýcká 129, 16500 Prague, Czech Republic; leuner@ftz.czu.cz

**Keywords:** phenylpropanoid, terpene, furanosesquiterpene, *Eremophila*, essential oil

## Abstract

Australia’s endemic desert shrubs are commonly aromatic, with chemically diverse terpenes and phenylpropanoids in their headspace profiles. Species from the genus *Eremophila* (Scrophulariaceae ex. Myoporaceae) are the most common, with 215 recognised taxa and many more that have not yet been described, widely spread across the arid parts of the Australian continent. Over the years, our research team has collected multiple specimens as part of a survey to investigate the chemical diversity of the genus and create leads for further scientific enquiry. In the current study, the diversity of volatile compounds is studied using hydrodistilled essential oils and leaf solvent extracts from 30 taxa. Several rare terpenes and iridoids were detected in chemical profiles widely across the genus, and three previously undescribed sesquiterpenes were isolated and are assigned by 2D NMR—*E*-11(12)-dehydroisodendrolasin, *Z*-11-hydroxyisodendrolasin and 10-hydroxydihydro-α-humulene acetate. Multiple sampling from *Eremophila longifolia*, *Eremophila arbuscular*, *Eremophila latrobei*, *Eremophila deserti*, *Eremophila sturtii*, *Eremophila oppositifolia* and *Eremophila alternifolia* coneys that species in *Eremophila* are highly chemovariable. However, taxa are generally grouped according to the expression of (1) furanosesquiterpenes, (2) iridoids or oxides, (3) mixtures of 1 and 2, (4) phenylpropanoids, (5) non-furanoid terpenes, (6) mixtures of 4 and 5, and less commonly (7) mixtures of 1 and 5. Furthermore, GC–MS analysis of solvent-extracted leaves taken from cultivated specimens conveys that many heavier ‘volatiles’ with lower vapour pressure are not detected in hydrodistilled essential oils and have therefore been neglected in past chemical studies. Hence, our data reiterate that chemical studies of the genus *Eremophila* will continue to describe new metabolites and that taxon determination has limited predictive value for the chemical composition.

## 1. Introduction

**Figure 1:**
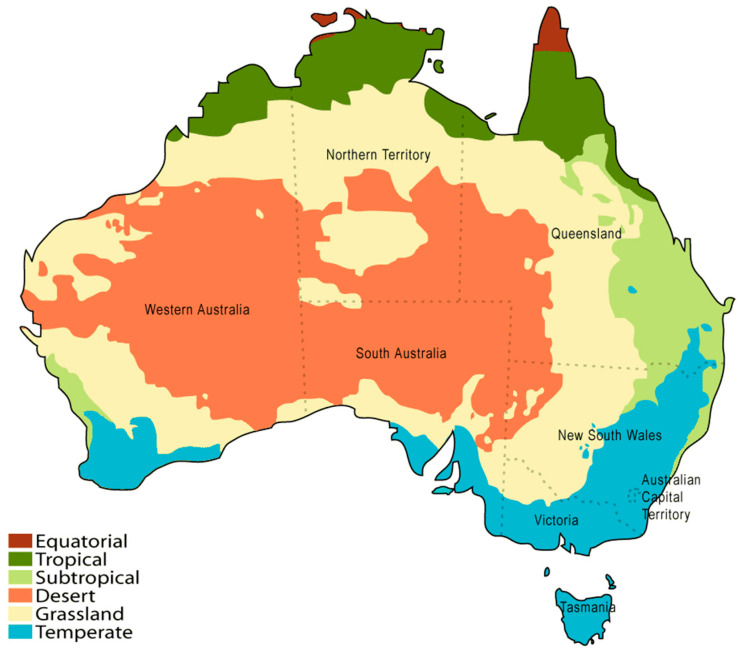
Australian climate map; image taken from https://en.wikipedia.org/wiki/Deserts_of_Australia (accessed on 15 January 2021).

The semi-desert ‘grassland’ region of Australia’s inner perimeter is in a transition zone between the sandy central Australian deserts and the tropical, subtropical, or temperate regions (Figure 1). In these semi-deserts, several species of *Eremophila* are found in dwindling populations [1] adjacent to sandstone or shale ranges that, on rare occasions, channel rainwater to near exhausted water pans underlain by red or grey cracking clay, which is where some of the highest essential oil-yielding aromatic species can be found. Such species include the diploid specimens of *Eremophila longifolia* (R.Br) F.Muell from Mutawintji National Park [2] and the myodesert-1-ene-yielding species *Eremophila dalyana* F.Muell of the ‘grey ranges’ in south-west Queensland [3].

In contrast, other moderately aromatic species are sparsely distributed across the undulating slopes and plains of western New South Wales, the sand-to-clay dermosols on the flat plains of central west Queensland and north-west Western Australia. Human habitation of these arid parts is sparse, particularly outside of the lake regions (i.e., Menindee Lakes), where farming practices include cattle and sheep grazing. Due to populous wild goats, the native flora is under considerable pressure, with some common species in rapid decline as grazing continues to denude the landscape. The so called ‘goat weed’ is a classic example; *Eremophila latrobei* F.Muell is often found with only a few remaining leaves. In contrast, with the disappearance of indigenous foliage, opportunistic native species are rapidly finding a niche. Both *Eremophila sturtii* R.Br and *Eremophila mitchellii* Benth. are becoming so common that they are regarded by some as a weed [4].

Previous phytochemical studies on the volatile organic compounds in species of *Eremophila* demonstrate considerable intraspecific variability, particularly in *E. longifolia* [2,5,6]. Furthermore, aromatic timbers express completely different chemical profiles as compared to their leaves. To illustrate this, the aromatic timbers of *E. mitchellii* were once regarded as a suitable alternative to the Australian sandalwood, *Santalum spicatum* (R.Br) A.DC., producing a termite repellent essential oil comprised of eremophilones and other derivatives [7]. As a substitute, it came to be regarded with distaste and earned the colloquial name ‘bastard sandalwood’ and was discontinued [8].

The etymology of the name *Eremophila* is related to its persistence in arid regions. The name is an amalgamation of the Greek words ‘eremos’, which means desert, and ‘philos’, which means friend (or platonic love); hence, *Eremophila* translates to ‘desert loving’. It is also believed that the ephemeral nature of the arid landscapes is important in persuading the expression of secondary metabolites. Preliminary observations of the effects of persistent drought and the opposite, persistent watering, convey that cycles of wet and dry periods favour the expression of specialised metabolites, particularly the volatile organic compounds [2]. Theoretically, this character survives from an ancestral ‘eremaeon’ stock of diploid *Eremophila* [9], which developed polyploidy in response to pressures from prolonged drought during post-glaciation events some 75K years ago [10], leading to speciation events and creating the diversity that we know of today. *Eremophila* is now comprised by more than 215 species [1], which include several that have not yet been described. Phytochemists have barely covered the full metabolite diversity of *Eremophila*. Over the years, we have retrieved several specimens for phytochemical screening and have elucidated geographically specific chemotypes in multiple species, discovered new terpenes and have made serendipitous observations. A summary of our most recent findings is presented in the current study, and some other species are briefly reviewed to convey the extent of the known chemistry of volatiles in this genus. 

## 2. Results and Discussion

It is common for studies of new essential oils from familiar genera to be comprised of a diversity of known volatile organic compounds. Hence, routine gas chromatography and mass spectrometry (GC–MS), together with a commercial mass spectral library (NIST) and retention indices are usually all that are necessary to chemically characterize new whole essential oils. 

However, volatiles from the genus *Eremophila* are different from those familiar to the species of the northern hemisphere and mass fragmentation spectrums are not present in commercial libraries. Identification of compounds is therefore strongly dependent on nuclear magnetic resonance spectroscopy and authentic standards. The current study chemically assigned 19 rare albeit known sesquiterpenes, and another three previously undescribed. In many cases, only single leaf specimens were studied, so many unknown compounds are highlighted to convey the full chemical diversity of the genus and the potential to identify previously undescribed metabolites.

Furthermore, the chemical profiles within species, and even within populations, can often be entirely different from specimen to specimen. This is illustrated in the current study. Out of the 30 taxa that we studied (Table 1 and Table 2), several replicates were collected for *Eremophila arbuscular*, *Eremophila latrobei*, *Eremophila alternifolia*, *Eremophila longifolia, Eremophila deserti, Eremophila oppositifolia* subsp. *rubra*, *Eremophila mitchellii* and *Eremophila sturtii*. In some cases, only hydrodistilled essential oils were studied but in other cases small leaf samples were taken, extracted in dichloromethane, and analysed by GC–MS. The distinction is explained through the text. 

Some species of *Eremophila* demonstrate a strong chemical overlap into species from *Myoporum*. This is particularly true of specimens that express furanosesquiterpenes in their metabolome. Furanosesquiterpenes are expressed in several species of *Eremophila* and most species of *Myoporum*. Over the years, the synonymity of these two genera has been debated and species are sometimes removed from one genus and placed in the other. This is evident from the high number of synonyms given to several of the species in *Eremophila*, particularly *E. deserti*.

### 2.1. Eremophila Deserti

**Figure 2:**
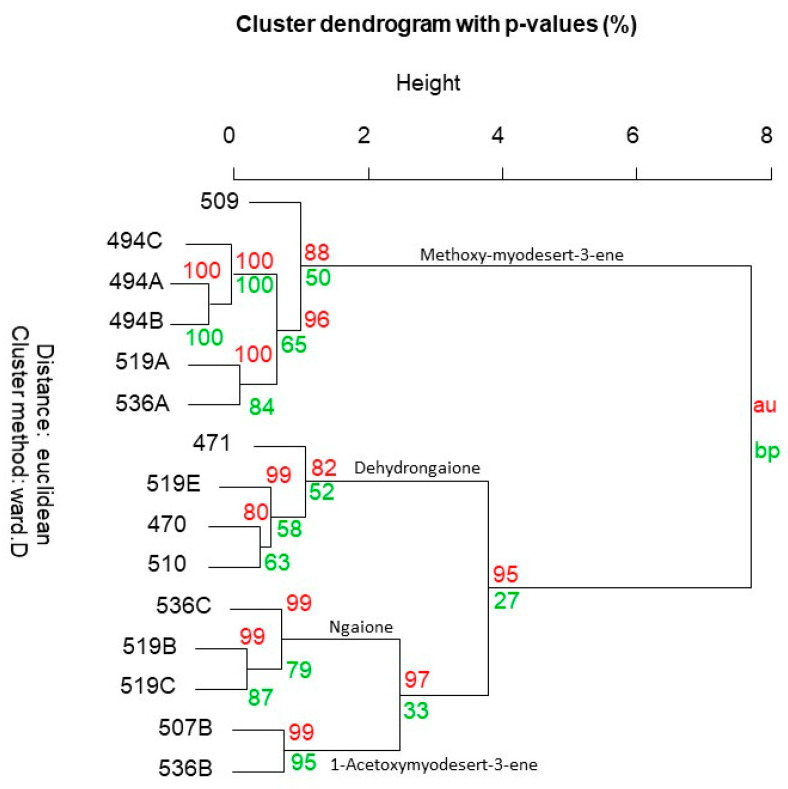
Dendrogram essential oil components from *Eremophila deserti*.

**Figure 3:**
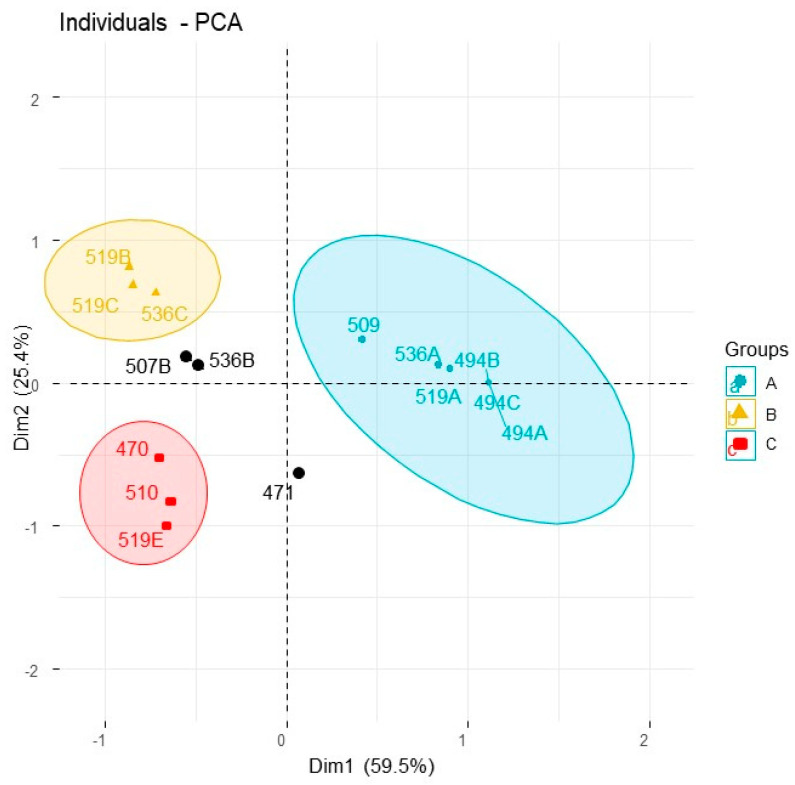
Principal component analysis of essential oil components from *Eremophila deserti*.

*Eremophila deserti* was previously known under the name *Myoporum deserti* and all the past chemical studies of the species were published under this previous name. Consequently, researchers occasionally overlook this, i.e., these details are not included in comprehensive reviews of the genus [11]. Our chemical study of *E. deserti* reiterated data of the species under its previous name *M. deserti*. Table 3 demonstrates the full chemical diversity of the species. By far the most common metabolite in leaves of *E. deserti* is methoxymyodesert-3-ene [12], which can vary in some specimens but is typically close to 100% of the gas chromatographic profile (the only component detected), which yields from the leaves at approximately 1–2% g/g of wet leaves. Furthermore, the cluster dendrogram of the chemistry of volatiles from the leaves of *E. deserti* conveys that the methoxymyodesert-3-ene type represents the most populated branch (Figure 2). In principal component analysis (Figure 3) this chemotype came out as group A. Hence, the methoxymyodesert-3-ene dominated specimens of *E. deserti* will henceforth be known as the ‘type A’ chemotype. The details of type A collection locations are provided in Table 2. 

Replicate samples of Ede-536 (A–C) and Ede-519 (A–D) (Table 2) were taken from individual specimens within common populations. What this conveys is that even within populations the individual specimens are expressing profiles of volatiles that conform to distinctly separate chemotypes in statistical analysis. Within the population found on the outskirts of Cunnamulla, SW Queensland (specimens Ede-519), one type A specimen was sampled out of four collections made within a 50 m radius. Alternatively, two of the specimens expressed a volatiles profile of >96% ngaione, which came out as type B in the PCA (Figure 3). The ngaione chemotype was previously recognised and published under the old name *M. deserti* [13]. Ngaione was recognised as responsible for the toxicity of the plant to grazing sheep. Ngaione from *Eremophila* or *Myoporum* were previously characterised as the levorotatory enantiomer of (+)-ipomeamarone [14]. 

A third chemotype was sampled from this location, which corresponds to type C in PCA (Figure 3), comprised by >93% dehydrongaione. Dehydrongaione was also previously recognised [15] for toxic effects to animals. By examination of the other collections made, the type C chemotype expresses variable amounts of dehydrongaione, in some cases as low as 49.5% of the profile as given by the GC–MS quantitation. 

One of the specimens (Ede-509B) was sampled twice at different growth stages. During the asexual phase in May (Ede-509B) it expressed methoxymyodesert-3-ene and conformed to the type A chemotype, but when the specimen had fruit on it in October (Ede-510) it demonstrated a chemical profile comprised by dehydrongaione, conforming to the type C chemotype. Hence, it is expected that some specimens will be intermediary between these two chemotypes during the year, i.e., one specimen also expressed >39% methoxymyodesert-3-ene in its profile (Ede-471). Furthermore, previous reports of inconsistency of the toxicity of the species may be explained by the changed expression of volatiles during the year. It is not yet clear whether the changes in expression profiles are truly reflective of growth stage or if another factor as yet unrecognised is involved.

A fourth chemotype was identified by two specimens that expressed 1-acetoxymyodesert-3-ene as the dominant component and myodesert-1-ene, *cis,cis*-nepetalactol, and *cis,cis*-nepetalactone as secondary components. The yield of essential oil of this chemotype was unusually low, i.e., 0.2–0.4%, compared with approximately 1–2% from other chemotypes. Furthermore, the current study constitutes the first report of myodesert-1-ene outside of *E. dalyana* [3]. Only two specimens were identified and segregated on a separate clade branch on the dendrogram in Figure 2, which were Ede-507B and Ede-536B. Due to limited samples, they were not recognised in PCA (Figure 3). However, this chemotype was described over 30 years ago, under the previous name *M. deserti* [16]. Hence, this chemotype will be recognised in the current study as Type D.

The current study demonstrates that there is no geographical agreement to the chemotypes identified in *E. deserti*. All chemotypes can be found within a single population. Furthermore, the two specimens that expressed 1-acetoxymyodesert-3-ene were found > 1000 km apart, one on the South Australian border near Broken Hill (Ede-536B) and the other on the Queensland border near Goondiwindi (Ede-507B). Hence, the profile of volatiles in *E. deserti* is extraordinarily plastic and diverse, probably changing chemistry in response to intrinsic rather than extrinsic cues.

Despite wide sampling, the authors of the current study found no specimens that were chemically like the ‘Jackson variety’ which contains myodesmone and isomyodesmone in its profile, or the ‘Theodore variety’ which expresses dehydromyodesmone and dehydroisomyodesmone [17]. Allegedly these myodesmone chemotypes had traces of myoporone in their volatile profiles [18]. Furthermore, the myodesmoid β-ketols that are derived from myoporone [19] were also not observed in any of the specimens of *E. deserti* that we sampled. These chemotypes are reportedly found in south eastern Qld locations that overlap with the locations visited by us. Hence, we cannot confirm or nullify these chemotypes. Since the taxonomic classification of species can be easily [4], it is important to verify these reports. Myodesmone isomers and their β-ketols are expressed abundantly in other species of *Myoporum* and were detected in other species of *Eremophila* in the current study, which is explained in the next section. 

**Table 3:** Essential oil chemistry of specimens determined to be *Eremophila deserti*.

Species Code (See Table 1 and Table 2)			470	471	494A	494B	494C	507B	509-May	510-Oct	519A	519B	519C	519E	536A	536B	536C	105
**Yield *w*/*w* % Fresh Leaves**			0.3	0.5	2.3	1.1	4.1	0.4	0.3	0.5	1.5	1.7	0.8	1.3	1.4	0.2	0.9	1.1
**Compound**	**AI**	**Pub AI**																
Myodesert-1-ene	1139	NMR	-	-	-	-	-	14.3	-	-	-	-	-	-	-	0.7	-	-
β-Pinene oxide	1161	1154	-	-	-	-	-	0.5	-	-	-	-	-	-	-	-	-	-
n.d.	1281	-	-	-	-	-	-	0.4	-	-	-	-	-	-	-	-	-	-
Methoxymyodesert-3-ene	1282	NMR	-	39.3	100.0	100.0	100.0	-	71.8	-	97.1	-	-	-	95.3	-	-	100
*Z,E*-Iridodial-1	1291	-	-	-	-	-	-	3.3	-	-	-	-	-	-	-	3.7	-	-
*Z,E*-Iridodial-2	1296	-	-	-	-	-	-	-	-	-	-	-	-	-	-	4.9	-	-
2-Ethylfenchol	1297	1297	-	-	-	-	-	4.8	-	-	-	-	-	-	-	-	-	-
*E,E*-Iridodial	1314	-	-	-	-	-	-	-	-	-	-	-	-	-	-	3.2	-	-
2*E*,4*E*-Decadienal	1316	1315	-	-	-	-	-	3.2	-	-	-	-	-	-	-	-	-	-
n.d.	1321	-	-	-	-	-	-	0.4	-	-	-	-	-	-	-	-	-	-
n.d.	1324	-	-	-	-	-	-	0.3	-	-	-	-	-	-	-	-	-	-
cis,cis-Nepetalactol	1335	-	-	-	-	-	-	3.4	-	-	-	-	-	-	-	10.2	-	-
α-Cubebene	1350	1345	-	-	-	-	-	0.4	-	-	-	-	-	-	-	-	-	-
β-Damascenone	1381	1383	-	-	-	-	-	0.2	-	-	-	-	-	-	-	-	-	-
α-Duprezianene	1390	1387	-	-	-	-	-	0.4	-	-	-	-	-	-	-	-	-	-
β-Elemene	1395	1389	0.7	0.8	-	-	-	1.0	-	1.7	-	-	-	-	-	0.7	-	-
cis,cis-Nepetalactone	1395	1391	-	-	-	-	-	1.2	-	-	-	-	-	-	-	0.6	-	-
*E*-Caryophyllene	1424	1417	1.2	0.5	-	-	-	1.2	-	2.3	-	-	-	-	-	1.5	-	-
β-Farnesene	1441	1440	-	-	-	-	-	0.2	-	-	-	-	-	-	-	-	-	-
β-Santalene	1455	1457	-	-	-	-	-	0.3	-	-	-	-	-	-	-	-	-	-
(1S)-1-Acetoxymyodesert-3-ene *	1460	NMR	-	-	-	-	-	23.8	-	-	-	-	-	-	-	48.4	-	-
(1S)-1-Acetoxymyodesert-3-ene epimer *	1471	NMR	-	-	-	-	-	0.8	-	-	-	-	-	-	-	3.5	-	-
Germacrene D	1485	1484	1.7	0.6	-	-	-	2.5	-	4.8	-	-	-	-	-	2.6	1.1	-
Bicydogermlacrene	1500	1500	3.7	2.5	-	-	-	11.1	-	3.1	-	0.3	-	-	-	6.1	2.1	-
δ-Cadinene	1524	1522	-	-	-	-	-	0.7	-	1.4	-	-	-	-	-	-	-	-
Spathulenol	1579	1577	-	1.0	-	-	-	1.9	-	-	-	-	-	-	-	0.9	-	-
Presillhiperfolan-8-β-ol	1585	1585	-	-	-	-	-	-	-	-	-	-	-	-	-	0.5	-	-
Gleenol	1585	1586	-	-	-	-	-	0.5	-	-	-	-	-	-	-	-	-	-
AIIo-cedrol	1589	1589	-	0.6	-	-	-	-	-	-	-	-	-	-	-	-	-	-
Viridiflorol	1593	1592	-	-	-	-	-	0.4	-	-	-	-	-	-	-	-	-	-
Carotol	1598	1594	-	-	-	-	-	0.4	-	-	-	-	-	-	-	-	-	-
Hinesol	1639	1640	-	-	-	-	-	0.3	-	-	-	-	-	-	-	-	-	-
Myomontanone	1647	NMR	-	-	-	-	-	-	-	-	-	-	-	-	-	0.6	-	-
Neo-intermedeol	1658	1658	-	-	-	-	-	-	-	-	-	-	-	-	-	0.9	-	-
Gymnomilrol	1658	1658	-	-	-	-	-	0.2	-	-	-	-	-	-	-	-	-	-
Bulnesol	1668	1670	-	-	-	-	-	0.5	-	-	-	-	-	-	-	-	-	-
*Z*-11-Hydroxyisodendrolasin	1678	NMR	-	-	-	-	-	0.2	-	-	-	-	-	-	-	0.6	-	-
Ngaione	1693	NMR	23.6	0.6	-	-	-	19.8	25.8	3.7	2.9	99.4	96.6	4.6	4.7	8.6	95.7	-
Epingaione	1695	NMR	-	-	-	-	-	0.3	-	-	-	-	1.4	-	-	-	-	-
Dehydroepingaione	1702	NMR	1.9	0.8	-	-	-	-	-	3.2	-	-	-	1.6	-	0.6	-	-
Dehydrongaione	1759	NMR	66.6	49.5	-	-	-	0.3	-	78.5	-	0.3	2.0	93.8	-	-	-	-
n.d.	1762	-	0.7	3.6	-	-	-	-	-	1.3	-	-	-	-	-	-	-	-
n.d.	1838	-	-	-	-	-	-	-	-	-	-	-	-	-	-	0.5	-	-

AI, arithmetic index; Pub. AI, published arithmetic index; * epimer deduced from the similarity of the mass spectrums. As previous studies only isolated the 1S enantiomer [16], it is correct to say that the enantiomer of 1-acetoxymyodesert-3-ene assigned in the current study is 1S; n.d., not determined.

### 2.2. Traditional Antibacterial Species

Unlike *E. deserti*, most other species of *Eremophila* have recorded or anecdotal traditional therapeutic uses [20]. The selection of species used in modern times is narrower, but there are nevertheless many that are used by following modern adaptions of traditional practices. Generally, the species that targeted topical infections have demonstrated in vitro antibacterial properties. Furthermore, there is a strong overlap between those species used by following smoke or fumigation modalities and those with antibacterial properties, suggesting a possible relationship. Hence, the headspace profiles of species with antimicrobial properties may include some of the antibacterial components or potentiators of other components.

Out of the taxa sampled for the current study, the species with multiple references of use in traditional therapeutic anti-infective applications include *E. alternifolia*, *E. duttonii*, *E. freelingii*, *E. latrobei*, *E. longifolia*, *E. mitchellii* and *E. neglecta*. Out of these seven, four have definite records of use in fumigation practice, but *E. alternifolia*, *E. duttonii* and *E. neglecta* are not known for such applications. However, fumigation of *E. alternifolia* was probably performed because it has a strong chemical similarity to *E. latrobei* and records of use in aromatherapeutic applications are also available [21].

#### 2.2.1. *Eremophila duttonii* and *E. neglecta*

Species with antimicrobial compounds in their headspace may also have fixed (non-volatile) antimicrobial compounds that required extraction into animal fats or out of leaf poultices (dermal extraction) in traditional use modalities, as practiced by traditional Australians [22] or Africans [23]. Furthermore, volatile organic compounds are known to exert synergistic or potentiating effects on fixed antimicrobial compounds [23,24,25]. The non-volatile antimicrobial compounds identified in *E. neglecta* [26,27], particularly 8-hydroxyserrulat-14-en-19-oic acid [27], are of the serrulatane class familiar to *E. duttonii* [28] or other species in *Eremophila* [29]. These non-volatile serrulatanes are known for antibacterial, antifungal and anti-biofilm effects [30]. In contrast, the volatile compounds from both species demonstrate only very modest antimicrobial effects. The essential oil of *E. neglecta* is predominantly monoterpenoid, with pinene isomers and sabinene as dominant components (Table 4), and the predominant volatile from *E. duttonii* is α-pinene. From the sesquiterpenes pool, *E. neglecta* produces farnesol and *E. duttonii* produces dehydrongaione.

Conversely, farnesol has been shown to promote biofilm formation [31]. Something that has not yet been considered is that the anti-biofilm effects of the serrulatanes are in place as a measure to antagonise the biofilm promoting effects of farnesol. It may therefore be necessary to look at the furanosesquiterpenes, such as dehydrongaione, to determine whether pro-biofilm effects are also enacted in vitro.

#### 2.2.2. *Eremophila freelingii*

Like *E. duttonii*, *E. freelingii* expresses an essential oil made up of common terpenes and a furanosesquiterpene. The hydrodistilled essential oil was comprised by α-pinene, spathulenol, myoporone and a tentatively identified β-ketol, which was similar to Redbank’s ketol (Table 4). The β-ketols were not isolated and assigned by NMR in the current study, so the structures are tentatively assigned by comparison with mass spectral data in an earlier paper [19]. Because the β-ketols are derivatives of myoporone to become oxidised myodesmone derivatives, it makes sense that they occur in *E. freelingii*, considering the presence of myoporone in the essential oil. The stereochemistry of the β-ketols was elaborated in a recent study by Australian and New Zealander chemists [32]. 

It was surprising that the specimens of *E. freelingii* that were sampled for the current study did not express freelingyne or freelingnite in the chemical profile. They were not in the essential oil (Table 4), nor in the solvent-extracted material (Table 5), which included four individuals from a population on the outskirts of Alice Springs, NT. The etymology of the name ‘freelingyne’ is evidently related to this species, from where it was first described [33], which is also the case for freelingnite [34]. However, these components were previously isolated from timber oil and might not be present in the leaves. Otherwise, the leaves also express long saturated carbon chains (n-alkanes or n-methylalkanes) (Table 5). The presence of bicyclogermacrene in solvent extracts, in contrast with spathulenol in essential oils, is not unusual. Although it is not clear how this occurs, material that is rich in bicyclogermacrene often yields essential oils with spathulenol [35], which is known to convert spontaneously, both in hydrodistillation and also at room temperature [36]. 

From the volatiles profile it is not obvious how smoke fumigation of *E. freelingii* could yield antimicrobial or antiseptic outcomes. However, in a previous study the heat derivative genifuranal was produced when hotter temperatures were used [37]. Genifuranal was first discovered in *E. longifolia* and realised to be responsible for the antimicrobial effects derived from fumigation rituals [38]. Other *Eremophila* used in smoke fumigation methodologies have not been tested this way. 

#### 2.2.3. *Eremophila alternifolia* and *E. latrobei*

Some of the β-ketols were also observed in the hydrodistilled essential oils from *E. alternifolia* (Table 6). Three specimens were collected for the current study from the plains around Broken Hill, NSW, one in 2012 (Ealt-170), one in 2013 (Ealt-261) and one in 2015 (Ealt-408). Some variation between specimens was evident, but only the relative amounts of components. This type of variation generally occurs as a response to extrinsic cues, such as amounts of rainfall or soil moisture retention, which is argued to influence chemical profiles in the genus *Prostanthera* [39,40] as well as *Eremophila*. 

In Australian species, phenoplasticity of volatiles caused by environmental cues is recognised by the increased expression of monoterpene components, which dilutes the sesquiterpenes and increases the yield of the essential oil. This is evident by examination of Ealt-408, which yielded more than 1% essential oil that was comprised by predominantly monoterpenoid components (>50%), compared with 10% and 25% monoterpenes in the other two specimens that yielded 0.6 and 0.7% essential oil, respectively (Ealt-170 and Ealt-261). The chemical profile of all specimens included common monoterpene components, such as α-fenchene, limonene and 1,8-cineol, and the rare sesquiterpene components myoporone and dehydromyoporone. 

The major β-ketol in Ealt-261 is tentatively identified as Carr’s ketol [32] (see Supplementary A3 for images), but this requires confirmation by spectroscopic analysis. This sesquiterpene is diluted when monoterpene expression is upregulated, with the main diluting component being fenchone. While there was only a small amount of fenchone in Ealt-408, a previous chemical characterisation of *E. alternifolia* described high yields (>4%) of fenchone dominated essential oils [41]. However, the high-yielding specimen under study by Barr [41] was collected from central Australia and represents a distinctly different chemotype to that of the current study. The central Australian people described *E. alternifolia* as their ‘number one’ medicine and included it in aromatherapeutic preparations, as previously mentioned. The Arrernte people also recognised some variants that were ‘stronger’ than others [42].

In horticulture, at least two varieties of *E. alternifolia* are recognised, i.e., *E. alternifolia* var *latifolia* and *E. alternifolia* var *alternifolia*. Nevertheless, from our chemical work, three chemotypes are evident—two in the current study and the fenchone type described by Barr [41]. Solvent-extracted material from a private garden of both varieties demonstrated strong chemical divergence between the two (Table 7). While *E. alternfiolia* var *alternifolia* was chemically consistent with the specimens we collected from Broken Hill, *E. alternifolia* var *latifolia* demonstrated a high relative abundance of myodesert-1-ene. Hence, this metabolite is now known from at least three taxa, with the others being *E. dalyana* [3], and the two specimens of *E. deserti* of the current study. Despite different monoterpenoid characters, the sesquiterpene components are similar between the two varieties. However, components with lower vapor pressures were not evident in the GC–MS spectrum beyond that of dehydromyoporone, conveying that essential oils are likely to be chemically like the gases from smoke fumigation practices that use higher temperatures. However, several unidentified components that were detected by GC–MS were extracted from *E. alternifolia* (Table 7), which conveys that the chemistry of hydrodistilled essential oils (Table 6) was different to the volatile components extracted into the solvent. 

The three garden specimens of *E. latrobei* represented the three subspecies—subsp. *filiformis*, subsp. *glabra* and subsp. *latrobei*. Some of the components extracted from garden specimens had retention indices as high as 2300–2450 (Table 7). This was the case for the two similar profiles from subsp. *filiformis* and subsp. *latrobei*. The profile from subsp. *glabra* did not have these larger components, but instead it was dominated by T-muurolol. This conveys at least two distinct chemical profiles from *E. latrobei*. 

The profile of monoterpenes and sesquiterpenes of garden specimens of *E. latrobei* was different by comparison with the specimens collected for hydrodistillation (Elat-269 and Elat-337) (Table 6). Furthermore, the essential oils from the two specimens of *E. latrobei* represented two further chemical profiles, in addition to those from the garden specimens, giving four in total. Specimen Elat-269 represents the grey leaf variety of subsp. *glabra*, which was collected from the conglomerate sandstone ranges of Mutawintji NP, on the border to South Australia. The profile was dominated by the sesquiterpene anymol, which is an epimer of the more common α-bisabolol. Anymol was first described in the wood essential oil of *Myoporum crassifolium* [43], which is from the sister genus to *Eremophila*. Specimen Elat-337 is determined as subsp. *filiformis*. This specimen also expressed a moderate amount of anymol but the profile included dehydrongaione and myoporone as major components. 

**Figure 4:**
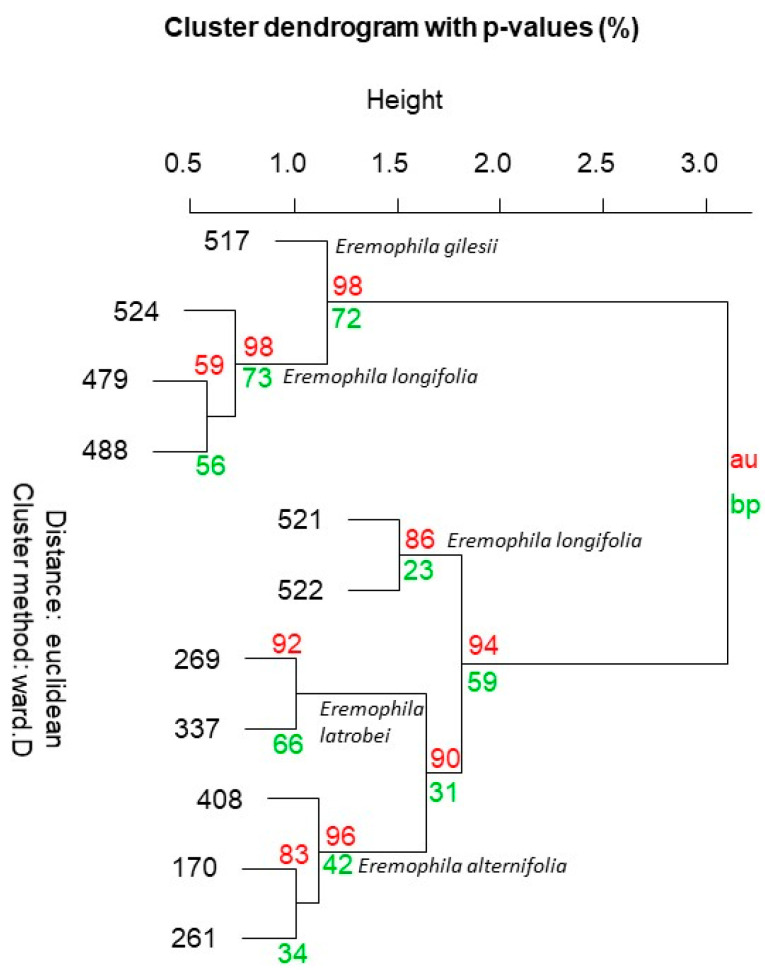
Dendrogram of essential oil components from *Eremophila longifolia*, *E. alternifolia* and *E. latrobei*.

**Figure 5:**
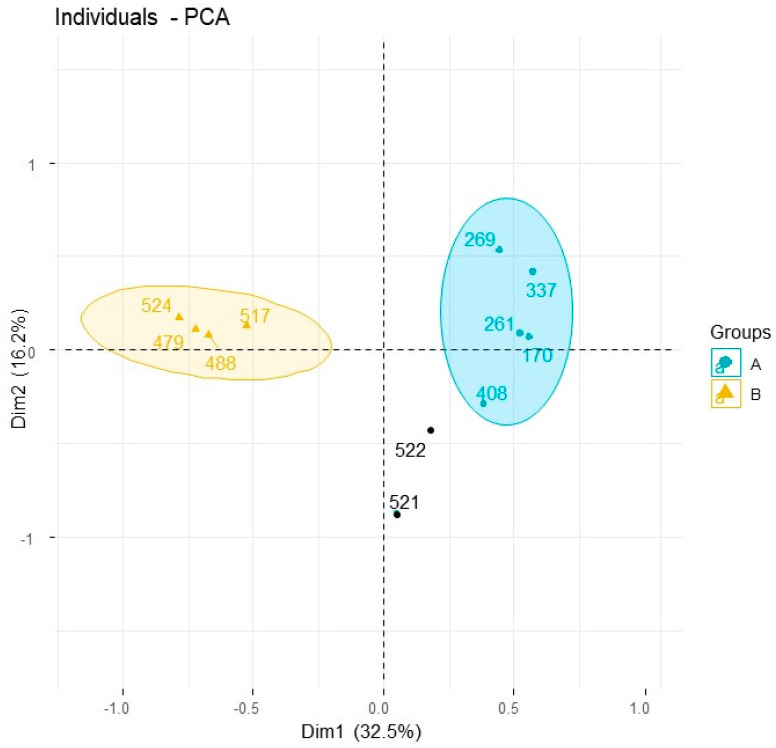
Principal component analysis of essential oil components from *Eremophila longifolia*, *E. alternifolia* and *E. latrobei*.

Multivariate analysis of hydrodistilled essential oil from *E. latrobei*, *E. alternifolia* and *E. longifolia* specimens demonstrated a close chemical agreement between *E. latrobei* and *E. alternifolia* in both a dendrogram (Figure 4), and PCA (Figure 5). The specimens of *E. longifolia* collected for the current study are chemically very distant from the two other species (Figure 4 and Figure 5).

#### 2.2.4. *Eremophila longifolia*

In a previous study of *E. longifolia* [2], one specimen with unusual leaf morphology, collected from the base of Kata-Tjuta, in central Australia, also expressed anymol in its profile, which was tentatively assigned as the epimer, α-bisabolol (type, J-4n), in that earlier study. However, only one specimen of *E. longifolia* demonstrated this profile. 

**Figure 6:**
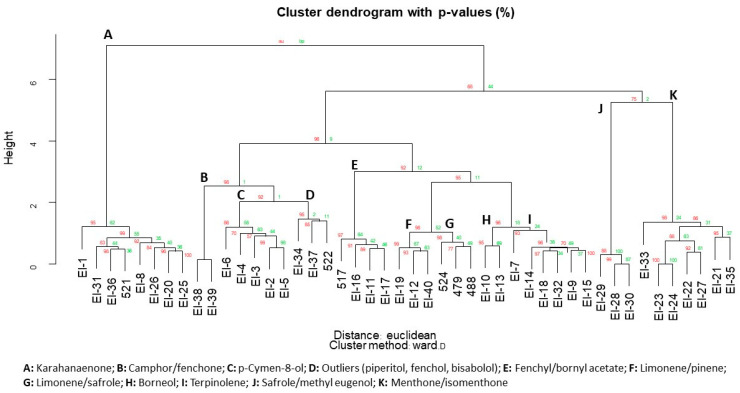
Dendrogram of essential oil components from *Eremophila longfilia* using data from the current study and a previous study.

The *E. longifolia* specimens of the current study, represent two additional intraspecific chemotypes. The chemical data from Table 6 were combined with the previously published dataset [2] to determine where these specimens are chemically placed. Multivariate analysis produced a dendrogram (Figure 6), which recognised 11 out of the 12 or 13 possible chemotypes. In addition to those described previously, a high-yielding chemotype that expressed piperitol as a major component of its essential oil was found in the grey ranges north of Thargomindah, SW Qld. The specimen bore a strong odour resemblance to the isomenthone type growing in Mutawintji NP, NSW, due south from its location. It is likely that this specimen is another of the diploids, extending the distribution to far north of Mutawintji NP. 

In travelling east from Mutawintji NP, one passes communities of lower essential oil-yielding diploids that express karahanaenone, which was also the case when traveling east from Thargomindah. However, the first ever specimens to be comprised by terpenes and phenylpropanoids were found in the same region and northwards, expressing moderate essential oil yields. Hence, the second new chemotype is the limonene/safrole type. This came as a surprise because previously only the diploid in far west Western Australia expressed safrole in its profile [2] and no other specimens from outside that region hitherto yielded a phenylpropanoid in its profile of volatiles. 

Full details of the other chemotypes in *E. longifolia* are provided in an earlier study [2]. Briefly, *E. longifolia* is characterised by several chemotypes that demonstrate both geographical patterns, and randomness. The karahanaenone type is often found as a single specimen in a population that is chemically different. However, on occasion this chemotype is also found as the dominant type in populous satellite communities. We have considered that the chemotype relates to a genotype that is created with sexual reproduction and propagated by sprouting of adventitious buds on the roots to create new trees. 

**Figure 7:**
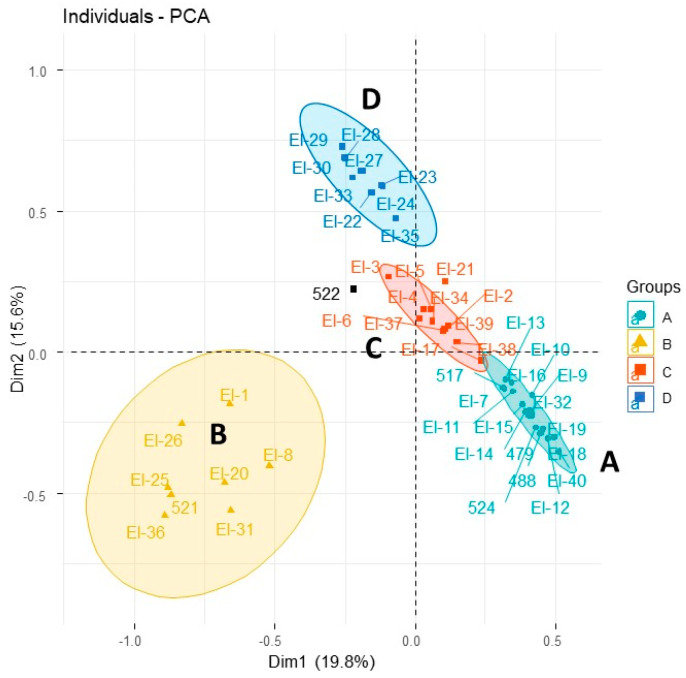
Principal component analysis of essential oil components from *Eremophila longifolia*.

In the current study, PCA was used to group all the chemotypes into four related groups (Figure 7). The karahanaenone specimens did not group with any other of the chemotypes (group B, Figure 7). However, group A and group C are differentiated by the relative amount of Limonene. Hence, group A represents chemotypes that include moderate to high amounts of limonene and group C represents reducing or no amounts of limonene, in moving along the diagonal distribution of specimens in each group (Figure 7). Lastly, group D includes the other diploids, excluding karahanaenone types. 

### 2.3. Taxonomic Misdeterminations

#### 2.3.1. *Eremophila mitchellii* and *E. sturtii*

The two species, *E. mitchellii* and *E. sturtii* have a history of misidentification. Serrulatic acids were isolated from *E. mitchellii* but misdetermined as *E. sturtii* [44]. Conversely, a new class of sesquiterpenes, the mitchellenes, were isolated from *E. sturtii* but misdermined as *E. mitchellii*, then named as mitchellenes rather than ‘sturtienes’ [45]. A revision to the phytochemistry of the two species was published later which increased the number of mitchellenes [4]. New mitchellenes maintain the same naming system.

**Figure 8:**
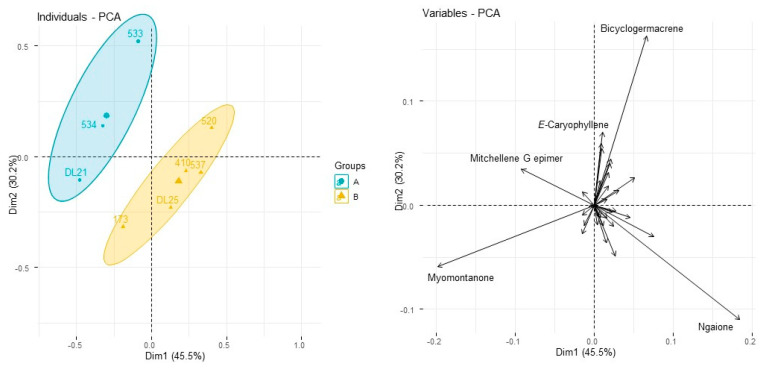
Principal component analysis including loadings plot of essential oil components from *Eremophila sturtii*.

A study of the chemical variation of hydrodistilled essential oils from eight specimens demonstrates that myomontanone is consistently present in the profile of volatiles from specimens widely distributed across NSW and south Qld (Table 8). However, from limited sampling, ngaione is expressed by some specimens but not others, with no clear geographical pattern. Mitchellene B is present in all hydrodistilled essential oils. Other mitchellenes, such as mitchellene G, and mitchellene isomers or epimers that have not been chemically assigned yet, were realised by examination of mass spectral data. Principal component analysis created two major groups (Figure 8) that are divided according to presence or absence of ngaione.

Leaves from four specimens of *E. mitchellii* were sampled to investigate possible chemical variation by comparison with the profiles reported by Beattie et al. [7]. A type of variation that is familiar to Australian species was evident, wherein the monoterpenoid components were absent from one specimen (Table 9). While most specimens expressed predominantly α-pinene and bicyclogermacrene, no α-pinene was expressed by the one specimen, Emi-181. Furthermore, timber essential oil (Emi-Wood) from a private collection received posthumously (Erich V. Lassak, 1934–2015) was fractionated and components assigned by NMR, revealing a chemical character identical to the specimen studied by Beattie et al. [7].

This has inspired a new view on phenoplasticity of volatiles in Australian species. Due to the perishable nature of leaves, accumulation of volatiles therein creates a chemical profile that records short term expression patterns. However, due to efficient storage of metabolites in timber, the metabolome therein is a record of long-term expression patterns, disguising the effects of phenoplasicity and improving metabolomic reproducibility. Because phenoplasticity of volatiles antagonises the taxonomic agreement in chemotaxonomic studies, it may be better to use timber, bark, or fragments of branches to create more precise chemical profiles within taxa. 

#### 2.3.2. *Eremophila arbuscular* and *E. oppositifolia*

The two species, *E. arbuscular* and *E. oppositifolia*, are commonly mis-determined as one for the other due to morphological similarities. In the current study, two specimens of *E. arbuscular* were harvested from Idalia National Park (NP), central Qld, several specimens of *E. oppositifolia* subsp. *rubra* and *E. oppositifolia* subsp. *oppositifolia* were harvested for comparison, from western NSW. 

Another specimen of *E. arbuscular* was harvested from a private garden on two separate occasions. The garden specimen bore the same distinctive odour of one of the wild specimens, which gave a furanosesquiterpene dominated essential oil profile (Table 4 and Table 10) when hydrodistilled. However, all three specimens were chemically different. The two wild specimens were collected based on a difference in the perception of aroma when the leaves were crushed and smelled, and the growth habit. 

In Idalia NP, the hills are populated with *E. arbuscular*, which grows into thin and tall trees (up to 10 meters) when in the ranges, but on flat soils it develops into a shorter and broader shrub. The data in Table 4 correspond to specimen Earb-486, which was collected from the ranges and Earb-487, which was from the flat plains below the same ridge. These specimens demonstrated strikingly different chemical profiles. Earb-486 expressed > 50% of a new metabolite which was characterised by NMR and assigned as *Z*-11-hydroxyisodendrolasin (see Supplementary A2 for NMR spectral assignments). In hydrodistillation, the hydroxy group is eliminated to afford *E*-11(12)-dehydroisodendrolasin (Supplementary A2), which was also assigned by NMR. Solvent extraction of fresh leaves and GC–MS analysis proved that the heat derivative is a product of hydrodistillation. Earb-486 also expressed the known metabolite 9-hydroxydendrolasin [46], which was reported in *E. rotundifolia* by Ghisalberti [47]. In contrast, Earb-487 from the flat soils expressed ~50% myomontanone, which is another furanosesquiterpene, but none of the dendrolasin derivatives were detected.

The garden specimen was sampled twice, once in 2015 (Earb-A, Table 10) and again in 2019 (Earb-B, Table 11). This specimen consistently expressed 9-hydroxydendrolasin, which is the metabolite responsible for the characteristic odour. However, it also expressed freelingnite, which was first described in *E. freelingii*. Freelingnite dominated the profile of volatiles, comprising 36–86% according to GC–MS, but was only present in the *E. arbuscular* in cultivation, and was not detected in the two wild specimens. This emphasises the strong chemical variability of the species. 

Surprisingly, subspecies of *E. oppositifolia*, i.e., subsp. *rubra* (EopR) and subsp. *oppositifolia* (EopO), bore no chemical resemblance to *E. arbuscular*. The volatiles profile of solvent-extracted material in Table 12 conveys that specimens of *E. oppositifolia* (EopO-538-B; EopR-535; EopR-539), are generally chemically different. All subspecies express oppositifolic acid (5-acetoxymethyltetradeca-trans-2,trans-4,trans-6-trienoic acid) [48] in the solvent extracts of leaves, which can be detected by GC–MS. However, the hydrodistilled essential oil from two specimens (Table 4; EopR-535-B, EopO-538A) produced much lower amounts of oppositifolic acid, because of the lower vapour pressure of this compound. The difference in specimens can be partly explained by differences of hydrodistillation time, with specimen EopO-538A having a shorter distillation time, which failed to drive the oppositifolic acid into the essential oil. 

**Table 4:** Chemistry of essential oils from *Eremophila neglecta* (Ene), *E. duttonii* (Edut), *E. foliosissima* (Efo), *E. platycalyx* (Epla), *E. gilesii* (Egi), *E. youngii* (Eyo), *E. freelingii* (Efre), *E. arbuscular* (Earb), *E. oppositifolia* subsp. *rubra* (EopR), and *E. oppositifolia* subsp. *oppositifolia* (EopO).

Species Code (See Table 1 and Table 2)			Ene	Edut	Epla-53	Efo-57	Epla-61	Epla-62	Egi-341	Eyo-345	Efre-346	Earb-486	Earb-487	EopR-535B	EopO-538A
**Yield *w*/*w* % Fresh Leaves**			0.3	0.4	0.2	0.1	0.1	0.1	0.1	2.8	0.3	0.3	0.1	0.2	0.2
**Compound**	**AI**	**Pub AI**													
1,3,5,7-Cyclooctatetraene	898	900	-	-	-	-	-	-	-	-	-	-	8.8	-	-
α-Thujene	928	924	1.7	-	-	0.3	-	2.2	-	-	-	-	-	-	-
α-Pinene	933	932	17.1	74.6	21.3	7.1	15.6	27.0	13.7	1.7	27.3	2.7	10.1	-	-
Camphene	946	946	-	1.2	13.1	-	0.7	26.2	-	-	-	-	-	-	-
3,5-Dimethyl-4-heptanone	966	973	-	-	-	-	-	-	-	-	-	-	8.1	-	-
Sabinene	970	969	34.4	-	-	0.5	-	-	-	-	-	-	-	-	-
β-Pinene	975	974	1.3	4.0	1.8	12.9	5.4	2.0	-	6.6	3.8	-	0.6	-	-
Butanoic acid, butyl ester	990	990	-	-	-	-	-	-	-	-	-	-	-	-	2.2
Myrcene	992	988	-	-	-	2.4	-	-	12.2	1.6	-	-	1.2	-	-
α-Phellandrene	1005	1002	1.4	-	-	-	-	-	3.2	-	-	-	-	-	-
α-Terpinene	1019	1014	4.4	-	-	-	-	-	-	1.2	-	-	-	-	-
*p*-Cymene	1026	1022	-	-	-	-	-	-	-	3.5	-	-	-	-	-
Limonene	1028	1024	-	0.9	7.0	1.7	0.6	7.7	-	1.7	-	0.9	2.4	-	-
β-Phellandrene	1032	1029	8.0	-	-	-	2.4	8.3	46.3	-	-	-	-	-	-
1,8-Cineole	1034	1026	-	-	-	-	-	-	-	73.1	-	-	-	-	-
β-Ocimene	1048	1044	-	-	-	0.3	-	-	0.6	-	-	1.0	-	-	-
γ-Terpinene	1060	1054	7.0	-	-	-	-	-	-	2.1	-	-	-	-	-
α-Terpinolene	1090	1086	1.7	-	-	-	-	-	-	-	-	-	-	-	-
Linalool	1098	1095	-	-	-	-	-	-	-	-	-	1.1	-	-	-
3-Methyl-3-butenyl-methyl butanoate	1111	1112	-	-	-	-	-	-	-	-	-	1.1	-	-	-
Terpinen-4-ol	1180	1174	4.7	-	-	-	-	-	-	6.1	-	-	-	-	-
Butanoic acid, 1-methylhexyl ester	1216	1210	-	-	-	-	-	-	-	-	-	-	-	-	7.9
n.d.	1240	-	-	-	-	-	-	-	-	-	-	-	0.6	-	-
n.d.	1279	-	-	-	-	-	-	-	-	-	-	-	2.0	-	-
Bornyl acetate	1294	1284	-	-	3.4	-	0.9	-	-	-	-	-	-	-	-
Geranyl acetate	1366	1362	-	-	-	-	-	3.2	-	-	-	-	-	-	-
β-Patchoulene	1383	1379	-	-	-	-	-	3.3	-	-	-	-	-	-	-
β-Elemene	1396	1389	-	-	-	1.5	1.1	-	1.3	-	-	-	-	-	-
β-Duprezianene	1418	1421	-	-	-	-	-	-	-	-	-	-	0.6	-	-
*E*-Caryophyllene	1425	1417	-	-	-	-	3.5	-	0.5	-	-	-	-	0.8	20.7
9-Epi-*E*-caryophyllene	1462	1464	-	-	-	0.4	-	-	-	-	-	-	-	-	-
1-Amorpha-4,7(11)-diene	1480	1479	-	-	-	0.3	-	-	-	-	-	-	-	-	-
*Z*-β-Guaiene	1485	1492	-	-	-	5.2	-	-	1.3	-	-	-	-	-	-
β-Selinene	1490	1489	-	-	-	0.4	-	-	0.5	-	-	-	-	-	-
Bicyclogermacrene	1497	1500	3.6	0.7	4.0	2.3	7.9	9.9	2.3	-	7.2	-	4.3	3.4	-
Eremophilene	1503	1502	-	-	-	1.5	-	-	0.6	-	-	-	-	-	-
δ-Cadinene	1522	1522	1.8	-	-	0.8	-	-	0.5	-	-	-	-	1.4	-
α-Cadinene	1529	1537	-	-	-	0.4	3.9	-	1.3	-	-	-	-	0.5	-
Elemol	1554	1548	-	-	-	11.0	-	-	-	-	8.2	-	-	-	-
Spathulenol	1579	1577	-	0.7	-	-	1.8	-	0.5	1.0	21.3	-	1.3	0.6	-
Caryophyllene oxide	1584	1582	2.6	-	-	-	-	-	-	-	-	-	-	0.9	29.4
Globulol	1585	1590	-	-	-	2.0	4.4	4.1	-	-	-	-	-	-	-
Cubeban-11-ol	1595	1595	-	-	-	-	-	-	0.7	-	-	-	0.6	1.5	-
Ledol	1596	1610	-	-	-	6.8	1.8	-	-	-	-	-	0.8	-	-
*E*-11(12)-Dehydroisodendrolasin	1597	NMR	-	-	-	-	-	-	-	-	-	10.8	-	1.2	-
Humulene epoxide II	1611	1608	-	-	-	-	-	-	-	-	-	-	0.5	1.4	-
Guaiol	1612	1600	-	-	-	-	4.6	-	-	-	-	-	-	3.1	-
10-Epi-7-eudesmol	1621	1622	-	-	3.1	1.0	-	-	-	-	-	-	1.5	1.0	-
γ-Eudesmol	1638	1630	-	-	2.3	0.4	0.6	-	0.8	-	-	-	-	0.5	-
*t*-Muurolol	1645	1644	-	-	-	-	-	-	-	-	-	-	-	42.9	-
Myomontanone	1647	NMR	-	-	-	-	-	-	1.3	-	-	-	49.2	-	-
Eudesmol isomer	1649	-	-	-	-	3.9	2.2	-	-	-	-	-	-	-	-
β-Eudesmol	1653	1649	-	-	23.1	1.4	4.1	3.3	3.5	-	-	-	-	-	-
α-Eudesmol	1657	1652	-	-	2.1	24.9	6.8	-	4.3	-	-	-	4.7	4.1	-
n.d.	1677		-	-	-	-	-	-	-	-	-	-	-	1.4	-
*Z*-11-Hydroxyisodendrolasin	1678	NMR	-	-	-	-	-	-	-	-	-	50.4	-	4.8	-
Anymol	1686	NMR	-	-	3.0	-	-	-	-	-	-	-	-	-	-
Epignaione	1694	NMR	-	-	-	-	-	-	-	1.3	-	-	-	-	-
n.d.	1700	-	-	-	3.7	-	-	-	-	-	-	-	-	-	-
n.d.	1715	-	-	-	4.2	-	-	-	-	-	-	-	-	-	-
Redbank’s Ketol	1721	*	-	-	-	-	-	-	-	-	10.2	-	0.7	0.6	-
Farnesol	1724	1722	10.3	-	4.6	-	26.0	2.8	3.3	-	-	3.8	0.6	0.6	-
9-Hydroxydendrolasin	1741	NMR	-	-	-	-	-	-	-	-	-	28.1	-	0.6	-
Dehydrongaione	1752	NMR	-	12.3	-	-	2.1	-	-	-	-	-	-	3.8	-
10R-Hydroxydihydro-α-humulene acetate	1755	NMR	-	-	-	-	-	-	-	-	-	-	-	-	39.7
n.d.	1760	-	-	-	-	7.3	-	-	-	-	-	-	-	-	-
Myoporone	1842	NMR	-	-	-	-	-	-	-	-	22.0	-	-	0.6	-
Phytol	1914	1915	-	5.7	-	-	-	-	-	-	-	-	-	-	-
Oppositifolic acid	2149	NMR **	-	-	-	-	-	-	-	-	-	-	-	24.1	-

AI, arithmetic index; Pub. AI, published arithmetic index; * tentatively assigned by mass spectral data. For more information, see Supplementary A3 or the following citation [32]; n.d., not determined. ** Known under another name as 5-acetoxymethyltetradeca-trans-2,trans-4,trans-6-trienoic acid [48].

**Table 5:** Chemistry of solvent extract volatiles from *Eremophila freelingii*.

			*Eremophila freelingii*—DCM Extract			
Species Code (See Table 1 and Table 2)			338-A	338-B	338-C	338-D
Compound ID	AI	Pub. AI				
α-Pinene	934	932	9.2	12.7	17.6	4.3
Germacrene D	1485	1484	-	-	-	2.8
Bicyclogermacrene	1500	1500	4.5	-	9.2	10.8
n.d.	-	-	1.7	-	-	-
Elemol	1554	1548	5.0	-	6.7	-
Myoporone	1836	NMR	23.2	17.3	37.2	54.2
Dehydromyoporone	1901	NMR	-	-	4.2	7.1
n.d.	-	-	6.0	4.1	5.2	6.7
2-Methyltetradecane	1461	1462 **	32.7	-	-	-
n.d.	-	-	4.6	3.9	5.9	5.1
n.d.	-	-	3.8	2.9	4.8	3.8
n.d.	-	-	5.4	3.9	5.2	5.2
Octacosane	1800	1800	-	5.9	-	-
n.d.-alkane	-	-	3.9	-	1.9	-
Nonacosane	1900	1900	-	49.3	2.1	-

AI, arithmetic index; Pub. AI, published arithmetic index; * no data found; n.d., not determined. Value found on Nist Chemistry Webbook.

**Table 6:** Chemistry of essential oils from *Eremophila longifolia* (Elo), *E. alternfiolia* (Ealt) and *E. latrobei* (Elat).

Species Code (See Table 1 and Table 2)			Elo-517	Elo-521	Elo-522	Elo-524	Elo-479	Elo-488	Ealt-170	Ealt-261	Ealt-408	Elat-269	Elat-337
**Yield *w*/*w* % Fresh Leaves**			0.7	0.8	3.2	0.9	0.4	0.1	0.6	0.7	1.2	0.4	0.5
**Compund Common Name**	**AI**	**Pub AI**	**-**	**-**	**-**	**-**	**-**	**-**	**-**	**-**	**-**	**-**	**-**
α-Thujene	924	924	-	-	-	-	-	-	-	0.5	2.5	-	-
α-Pinene	932	932	-	-	-	0.9	5.6	2.0	-	1.5	6.6	-	-
α-Fenchene	945	945	2.0	-	-	-	-	-	2.3	7.0	23.2	1.3	-
Sabinene	970	969	-	-	-	-	6.5	11.4	-	-	-	-	-
β-Pinene	975	974	-	-	-	-	-	-	-	1.7	2.9	-	-
δ-Carene	998	1001	-	-	1.1	-	1.6	3.7	-	-	-	-	-
α-Phellandrene	1007	1002	-	-	2.8	-	0.8	0.8	-	0.8	2.9	-	-
α-Terpinene	1016	1014	-	-	1.1	0.7	1.4	2.0	-	-	1.5	-	-
p-Cymene	1024	1022	-	-	-	-	-	-	1.0	0.4	0.8	-	-
β-Phellandrene	1026	1025	-	-	4.9	-	-	-	-	3.4	-	-	-
Limonene	1028	1024	38.5	3.7	-	69.4	62.4	58.5	0.8	-	12.7	-	-
1,8-Cineole	1031	1026	-	-	-	-	-	-	1.1	7.8	19.5	-	-
γ-Terpinene	1059	1054	-	-	-	-	1.3	2.3	-	0.4	1.7	-	-
α-Terpinolene	1087	1086	-	-	-	1.8	8.7	15.3	-	-	-	-	-
Fenchone	1087	1083	1.3	-	-	-	-	-	-	-	4.9	-	-
*E*-Mentha-2,8-dien-1-ol	1119	1119	-	-	3.7	-	-	-	-	-	-	-	-
Geijerene	1141	1138	-	-	1.9	-	-	-	-	-	-	-	0.3
Karahanaenone	1154	1154	-	80.9	0.5	-	-	-	-	-	3.1	-	-
Isomenthone	1163	1158	-	-	26.4	-	-	-	-	-	-	-	-
Borneol	1165	1165	2.7	-	-	-	-	-	-	-	-	-	-
Terpinen-4-ol	1179	1174	0.4	4.7	-	-	1.3	3.4	0.9	0.8	2.2	-	-
α-Terpineol	1190	1186	1.5	1.1	11.3	-	-	-	-	-	1.1	-	-
*Z*-Piperitol	1196	1195	-	-	42.1	-	-	-	-	-	-	-	-
*E*-Piperitol	1206	1207	-	-	2.2	-	-	-	-	-	-	-	-
Fenchyl acetate	1219	1218	10.0	-	-	1.6	-	-	-	-	-	-	-
Nerol	1227	1227	-	-	-	-	-	-	2.9	-	-	-	-
Asearidole	1237	1234	-	-	0.4	-	-	-	-	-	-	-	-
Piperitone	1252	1249	-	-	0.5	-	-	-	-	-	-	-	-
2-(1*E*)-Propenyl-phenol	1266	1264	-	-	-	-	3.5	-	-	-	-	-	-
Bornyl acetate	1285	1287	43.0	-	-	5.3	-	-	-	-	-	1.0	-
Safrole	1288	1287	-	-	-	20.4	5.6	-	-	-	-	-	-
3-Thujanol acetate	1297	1295	-	-	-	-	-	-	-	-	-	1.8	-
p-Vinyguaiacol	1311	1309	-	4.8	0.7	-	-	-	-	-	-	-	-
Dictamnol	1429	1428	-	-	-	-	-	-	-	-	-	-	0.7
Alloaromadendrene	1440	1439	-	-	-	-	-	-	0.4	-	-	-	-
β-Macrocarpene	1496	1499	-	-	-	-	-	-	-	-	0.5	-	-
β-Germacrene	1501	1508	-	-	-	-	-	0.7	-	-	-	-	-
Bicyclogermacrene	1501	1500	-	-	-	-	1.4	-	-	0.5	-	-	-
δ-Cadinene	1523	1522	-	-	-	-	-	-	0.4	0.5	0.5	-	0.2
Kessane	1535	1529	-	-	-	-	-	-	-	-	-	1.0	-
n.d.	1549	-	-	-	-	-	-	-	-	-	-	3.6	3.1
Maaliol	1568	1566	-	-	-	-	-	-	0.5	-	-	-	0.3
Spathulenol	1579	1577	-	-	-	-	-	-	8.4	0.6	0.4	-	1.7
Cedrol	1591	1600	-	-	-	-	-	-	1.4	-	0.5	3.0	-
Rosifoliol	1603	1600	-	-	-	-	-	-	0.5	-	-	3.4	-
n.d.	1614	-	-	-	-	-	-	-	-	-	-	2.4	0.2
10-Epi-γ-eudesmol	1621	1622	-	-	-	-	-	-	-	-	-	2.0	1.4
Eudesmol	1624	1622	-	0.9	-	-	-	-	1.6	-	0.7	-	-
γ-Eudesmol	1632	1630	-	-	-	-	-	-	-	-	-	1.2	0.3
Hinesol	1642	1640	-	-	-	-	-	-	0.6	0.4	0.6	2.5	1.4
Myomontanone	1647	NMR	-	-	-	-	-	-	5.9	3.4	1.0	1.9	2.3
Isomyodesmone	1650	*	-	-	-	-	-	-	0.7	-	-	4.5	3.7
Carr’s Ketol	1658	*	-	-	-	-	-	-	4.5	39.0	0.8	1.9	7.3
Bulnesol	1668	1670	-	-	-	-	-	-	-	-	-	1.5	0.4
n.d.	1677	-	-	-	-	-	-	-	2.8	-	0.8	2.7	2.6
Kindon’s Ketol	1681	*	-	-	-	-	-	-	-	1.1	-	-	2.2
Anymol	1686	NMR	-	-	-	-	-	-	-	-	-	63.5	19.4
Epignaione	1697	NMR	-	-	-	-	-	-	-	-	-	-	26.8
n.d.	1726	-	-	-	-	-	-	-	0.4	-	-	-	5.9
Carney’s Ketol	1734	*	-	-	-	-	-	-	-	4.7	-	-	0.2
Dehydrongaione	1752	NMR	-	-	-	-	-	-	-	-	-	-	0.4
Myoporone	1836	NMR	-	3.3	-	-	-	-	19.4	22.7	5.0	-	17.4
Dehydromyoporone	1901	NMR	-	-	-	-	-	-	43.2	1.8	3.3	-	0.9

*Eremophila longifolia* (Elo), *E. alternfiolia* (Ealt) and *E. latrobei* (Elat); AI, arithmetic index; Pub. AI, published arithmetic index; * tentatively assigned by mass spectral data. For more information, see Supplementary A3 or the following citation [32]; n.d., not determined.

**Table 7:** Solvent extract volatiles from *Eremophila alternifolia* (Ealt) and *E. latrobei* (Elat).

Species Code (See Table 1 and Table 2)			EaltMm	Ealt-A	Ealt-B	Ealt-C	EaltL-A	EaltL-B	EaltL-C	ElatF	ElatG	ElatL
**Common Name**	**AI**	**Pub. AI**	-	-	-	-	-	-	-	-	-	-
α-Thujene	929	924	2.8	3.7	2.6	1.6	-	-	-	-	-	-
α-Pinene	934	932	6.7	10.0	7.3	5.5	2.6	1.9	1.8	-	0.7	0.4
α-Fenchene	945	945	-	-	12.0	9.7	14.1	6.9	6.4	6.3	0.5	7.4
Sabinene	970	969	4.6	8.9	5.3	4.4	0.3	0.5	-	-	-	-
β-Pinene	975	974	1.6	-	-	-	-	-	-	-	-	-
α-Phellandrene	1007	1002	5.5	-	1.4	-	1.5	1.0	-	-	-	-
p-Cymene	1026	1022	0.5	0.7	0.3	0.7	-	-	-	-	-	-
β-Phellandrene	1032	1029	11.5	11.9	8.9	7.5	7.1	9.3	8.5	-	-	-
1,8-Cineole	1034	1026	10.2	35.1	15.6	21.2	0.4	0.7	1.0	-	-	-
γ-Terpinene	1059	1054	0.2	-	0.3	0.2	0.2	-	-	-	-	-
*Z*-Sabinene hydrate	1065	1065	0.3	1.0	0.7	1.2	0.3	-	-	-	-	-
α-Terpinolene	1087	1086	0.2	-	-	-	2.8	2.0	-	-	-	-
*E*-Sabinene hydrate	1097	1098	0.1	0.4	0.3	0.3	-	-	-	-	-	-
Myodesert-1-ene	1139	NMR	-	-	-	-	21.5	46.2	64.4	-	-	-
Terpinen-4-ol	1180	1174	-	-	0.3	0.4	0.8	1.2	1.8	-	-	-
α-Terpineol	1190	1186	-	0.4	0.2	0.3	-	-	-	-	-	-
Endo-fenchyl acetate	1219	1218	-	-	-	-	-	-	-	0.7	-	1.1
Nerol	1230	1232	-	-	0.8	1.1	-	-	-	-	-	-
Piperitone	1253	1249	-	-	0.5	0.7	-	-	-	-	-	-
Methoxymyodesert-3-ene	1282	NMR	-	-	-	-	-	-	-	1.1	1.4	1.4
p-Vinylguaiacol	1313	1309	0.3	1.8	1.0	2.3	-	0.8	-	-	-	-
*cis,cis*-Nepetalactol	1335	-	-	-	0.2	-	-	-	-	-	-	-
*cis,cis*-Nepetalactone	1395	1391	-	-	-	0.4	-	-	-	-	-	-
*E*-Caryophyllene	1425	1417	-	-	-	-	-	-	-	3.9	0.5	-
β-Santalene	1455	1457	-	-	-	-	-	-	-	2.3	0.8	-
(1S)-1-Acetoxymyodesert-3-ene *	1460	NMR	0.2	-	-	-	0.3	0.6	-	-	-	-
(1S)-1-Acetoxymyodesert-3-ene epimer *	1471	-	0.2	-	0.5	-	0.4	1.1	-	-	-	-
Germacrene D	1485	1484	-	-	-	-	-	-	-	1.2	-	-
Bicydogermlacrene	1500	1500	1.2	0.7	2.6	0.9	2.7	1.9	-	0.7	0.6	1.8
δ-Cadinene	1524	1522	-	-	0.2	-	-	-	-	-	-	-
Elemol	1548	1548	-	-	-	-	-	-	-	-	2.1	-
n.d.	1563	-	-	2.1	1.2	3.2	-	0.6	-	0.7	2.1	0.3
n.d.	1575	-	0.8	1.3	2.7	2.3	0.3	1.0	-	-	-	-
Caryophyllene oxide	1583	1583	-	-	-	-	-	-	-	0.4	-	-
Cedrol	1591	1600	-	-	-	-	1.3	2.7	5.2	-	0.7	-
Rosifoliol	1603	1600	-	1.6	0.5	2.0	-	-	1.0	-	-	-
Humulene epoxide II	1611	1608	-	-	-	-	-	-	-	5.6	-	4.8
10-Epi-7-eudesmol	1621	1622	-	-	0.2	0.8	-	-	-	-	-	-
Muurola-4,10(14)-dien-1-β-ol	1628	1630	-	-	-	-	-	-	-	-	3.2	2.2
*t*-Muurolol	1645	1644	-	-	-	-	-	-	-	1.0	35.5	0.6
Myomontanone	1647	1646	-	1.4	0.2	-	1.4	-	-	-	-	-
Isomyodesmone	1650	1649	-	-	-	-	-	-	-	-	3.7	2.5
Myodesmone	1651	-	-	1.2	1.7	-	6.4	3.4	-	-	-	-
Bulnesol	1668	1670	0.4	0.9	5.5	10.3	-	-	-	-	0.4	-
Ketol (Kindon, Perillup or Carr’s ketol) **	1681	**	-	-	1.4	-	3.1	1.2	-	-	-	-
Ngaione	1682	NMR	-	-	0.2	0.7	-	-	-	-	0.8	-
Dehydroepingaione	1702	NMR	-	-	-	-	-	-	-	-	0.5	-
Ketol (like Carney’s ketol) **	1721	**	0.2	-	1.3	-	0.5	-	-	-	-	-
Diisopropylnaphthalene	1724	1716	4.4	-	0.9	-	3.7	-	-	-	-	-
Ketol (like Carney’s ketol) **	1734	**	-	-	-	-	-	-	-	-	0.4	-
n.d.	1746	-	-	-	-	-	-	-	-	-	0.5	-
n.d.	1788	-	-	-	-	0.2	-	-	0.9	-	1.0	-
n.d.	1802	-	-	0.4	-	2.5	-	-	-	-	-	-
n.d.	1811	-	-	-	-	-	-	-	-	-	0.4	-
n.d.	1815	-	0.8	-	-	-	-	-	-	-	-	0.6
n.d.	1819	-	0.4	-	-	-	-	-	-	-	0.6	-
Myoporone	1836	NMR	0.3	1.5	4.8	1.0	15.2	6.8	0.8	-	2.1	-
n.d.	1846	-	0.7	-	0.7	0.4	0.5	-	-	-	-	-
n.d.	1852	-	-	-	-	-	-	-	-	-	5.7	-
n.d.	1864	-	0.3	0.5	0.2	0.3	-	-	-	-	1.2	-
n.d.	1868	-	-	-	-	-	-	-	-	-	1.2	-
n.d.	1872	-	-	-	0.4	0.4	-	-	-	-	0.5	-
n.d.	1882	-	-	-	-	-	-	-	-	-	0.6	-
n.d.	1890	-	-	-	-	-	-	-	-	-	4.4	-
Dehydromyoporone	1901	NMR	12.7	0.6	8.4	0.6	8.3	2.8	-	-	1.4	-
n.d.	1913	-	-	-	-	-	-	-	-	-	8.3	-
n.d.	1919	-	0.6	0.8	0.4	1.2	-	-	-	-	0.9	-
n.d.	1927	-	-	-	1.3	2.4	-	-	-	-	-	-
n.d.	1948	-	0.2	-	-	-	-	-	-	-	-	-
n-Hexadecanoic acid	1964	1960	0.6	-	0.9	0.3	0.6	1.3	0.7	-	-	-
n.d.	1958	-	0.3	-	-	-	-	-	-	-	-	-
n.d.	1962	-	0.4	-	-	-	-	-	-	-	-	-
n.d.	1974	-	-	-	-	-	-	-	-	-	1.0	-
Freelingnite	1985	NMR	10.6	-	-	-	-	-	-	-	-	-
n.d.	2017	-	-	8.0	-	0.4	-	-	0.8	-	1.6	-
n.d.	2058	-	9.5	-	-	-	-	-	-	-	-	-
n.d.	2072	-	-	-	-	-	-	-	-	-	0.6	-
n.d.	2078	-	-	1.2	-	0.8	-	-	-	-	0.9	-
n.d.	2083	-	0.2	-	0.3	0.9	-	-	-	-	1.0	0.6
n.d.	2089	-	2.6	1.0	1.4	3.6	0.3	-	-	-	-	0.7
Phytol	2100	2100	3.2	1.0	1.0	2.5	1.8	2.4	4.7	1.9	6.2	5.2
n.d.	2124	-	1.6	-	1.4	0.9	1.5	3.0	2.1	0.6	1.2	2.7
n.d.	2190	-	-	-	-	-	-	-	-	0.4	-	0.6
n.d.	2258	-	0.3	-	-	-	-	-	-	2.4	-	-
n.d.	2336	-	-	-	-	-	-	-	-	41.7	-	27.5
n.d.	2346	-	-	-	-	-	-	-	-	1.7	-	-
n.d.	2349	-	-	-	-	-	-	-	-	6.5	-	2.4
n.d.	2362	-	-	-	-	-	-	-	-	3.4	-	-
n.d.	2398	-	-	-	-	-	-	-	-	15.5	-	-
n.d.	2471	-	-	0.8	-	-	-	-	-	1.0	-	32.1

AI, arithmetic index; Pub. AI, published arithmetic index; n.d., not determined. * epimer deduced from mass spectral similarity. As previous studies only isolated the 1S enantiomer [16] it is correct to say that the enantiomer of the current study is 1S; ** for information on possible structures see the following citation [32].

**Table 8:** Essential oils from *Eremophila sturtii* leaves.

Specimen Code (See Table 1 and Table 2)			533	534	537	DL-21 **	173	DL-25 **	410	520
**Yield *w*/*w* Wet Leaf**			0.51	0.42	0.21	0.39	0.41	0.42	0.25	0.26
**Compound Name**	**AI**	**Pub. AI**								
α-Pinene	933	932	-	-	-	0.9	-	1.9	0.7	0.7
Unknown	1327	nf	-	-	0.9	1.7	-	0.8	2.2	1.1
*δ*-Elemene	1337	1338	1.0	-	0.3	-	-	-	-	0.6
*E*-Caryophyllene	1421	1417	3.1	1.5		-	-	0.2	0.9	1.5
Aromadendrene	1440	1439	1.4	0.6	0.7	-	-	-	0.5	0.9
α-Humulene	1455	1452	0.8	-	-	-	-	0.5	0.3	0.4
9-Epi-*E*-caryophyllene	1469	1464	4.2	2.0	-	-	-	0.2	1.1	2.1
*t*-Muurolene	1484	1479	1.4	0.7	6.1	1.3	-	0.6	2.3	0.8
Bicyclogermacrene	1497	1500	26.1	4.7	8.0	0.9	-	0.4	6.0	14.2
δ-Cadinene	1523	1524	0.5	-	-	-	-	-	-	0.3
Cedranoxide, 8,14- (isomer)	1529	-	5.3	7.5	10.2	11.5	11.2	17.3	17.6	11.8
Cedranoxide, 8,14-	1545	1541	3.4	5.2	5.9	6.9	7.1	10.6	10.6	6.9
Unknown	1565	-	0.4	0.8	0.7	1.0	1.4	1.8	1.4	1.1
Spathulenol	1578	1577	10.4	5.5	6.0	1.7	4.5	3.8	3.5	7.1
Caryophyllene oxide	1585	1582	-	1.5	1.1	-	1.0	0.5	-	1.6
Globulol	1593	1590	2.4	0.9	0.8	-	-	-	-	0.9
Cedrol	1603	1600	0.5	1.4	1.5	-	0.6	3.7	4.1	1.5
Unknown	1607	-	-	1.5	1.0	2.6	1.5	0.6	0.3	1.7
γ-Eudesmol	1629	1630	-	-	0.3	-	1.1	3.2	3.0	7.1
Myomontanone	1647	NMR	22.6	38.1	13.6	52.8	49.8	18.9	13.1	5.0
α-Eudesmol	1653	1652	2.0	-	3.0	-	-	0.8	0.6	1.0
7-Epi-α-eudesmol	1666	1662	-	0.6	0.5	-	-	1.1	0.2	0.5
Mitchellene isomer 1	1676	-	-	0.8	0.9	0.9	-	0.4	2.0	0.3
Mitchellene isomer 2	1680	-	-	1.1	0.9	1.0	-	-	0.7	0.2
Ngaione	1688	NMR	-	-	29.4	-	11.3	11.2	12.9	15.5
Mitchellene G epimer	1689	*	1.3	5.0	-	6.4	-	-	-	-
Mitchellene G	1695	NMR	3.9	6.3	3.5	4.3	4.5	8.2	7.1	5.2
*cis*-Nuciferol	1711	-	-	1.4	0.9	-	-	1.6	0.3	1.0
*trans*-Nuciferol	1725	1724	-	1.8	1.0	1.4	-	1.5	1.0	-
Dehydrongaione	1752	NMR	-	-	-	-	-	-	-	0.8
Myoporone	1836	NMR	-	0.8	-	-	0.7	-	0.2	-
Mitchellene B	2083	NMR ***	2.7	8.5	1.6	2.0	2.0	3.1	4.3	2.3

AI, arithmetic index; Pub. AI, published arithmetic index; n.d., not determined; NMR, determined by nuclear magnetic resonance spectroscopy; * no information found; ** Collector’s reference D. Lyddiard 21 and D. Lyddiard 25. *** Matched to published spectra [45].

**Table 9:** Chemistry of essential oils from *Eremophila mitchellii*.

Species Code (See Table 1 and Table 2)			93	181	436	541	Emi-Wood
**Yield g/g Wet Leaves**			0.2	0.13	0.21	0.11	1.5
**Compound Name**	**AI**	**Pub. AI**					
α-Pinene	933	934	40.1	-	38.3	52	-
β-Pinene	977	980	-	-	-	1.1	-
α-Phellandrene	1005	1007	1.9	-	1.8	7.8	-
p-Cymene	1024	1024	-	-		1.3	-
Limonene	1028	1026	1.5	-	1.4	3.2	-
Terpinolene	1088	1088	0.9	-	0.9	1.9	-
δ-Elemene	1337	1335	1.2	1.7	1.2	-	-
β-Patchoulene	1382	1379	-	0.8	0.4	1.3	-
β-Elemene	1392	1389	1.1	0.6	1.1		-
α-Gurjunene	1410	1409	-	0.7	0.6	1	-
Caryophyllene	1420	1417	1.2	1.1	1.1	1.7	-
10-Epi-γ-eudesmol	1428	1422	-	0.6	-	0.7	-
Aromadendrene	1440	1439	2.4	4.8	2.3	5.1	-
Alloaromadendrene	1462	1460	-	0.7	-	0.6	-
γ-Gurjunene	1487	1475	-	0.5	-	-	-
β-Selinene	1489	1489	-	0.7	-	-	-
Bicyclogermacrene	1497	1500	31.3	46.8	29.8	13.7	-
δ-Cadinene	1523	1522	1.7	-	1.6	-	-
Germacrene B	1561	1559	-	0.6	-	-	-
n.d.	1569	-	-	1.2	0.6	-	-
Spathulenol	1578	1577	6.6	14.2	6.3	3.3	-
Globulol	1585	1590	2.6	6.8	2.5	3.1	-
Viridiflorol	1593	1592	1.6	4.2	1.5	1.5	-
Guaiol	1612	1600	-	1.6	-	-	2.5
10-Epi-γ-eudesmol	1623	1622	-	3.9	-	-	-
α-Muurolol	1639	1640	-	1.1	6.7	-	-
*t*-Muurolol	1654	1644	5.8	1.6	0.4	-	-
α-Eudesmol	1657	1652	-	-	-	-	2.7
Eremophilone	1740	1736 *	-	-	-	-	50.6
Santalcamphor	1760	NMR *	-	-	-	-	29.1
8-Hydroxy-1,11-eremophiladien-9-one	1764	NMR *	-	-	-	-	2.8
9-Hydroxy-7(11),9-eremophiladien-8-one	1849	NMR *	-	-	-	-	11.8
n.d.	2107	-	-	3.8	-	-	-

AI, arithmetic index; Pub. AI, published arithmetic index; n.d., not determined; NMR, determined by nuclear magnetic resonance spectroscopy. * Spectra compared to published values [7].

**Table 10:** Chemistry of solvent extract volatiles from *Eremophila platycalyx* (Eplat), *E. arbuscular* (Earb), *E. goodwinnii* (Ego), *E. bowmannii* (Ebo), *E. arachnoides* (Ear), *E. dalyana* (Edal), *E. spectabilis* (Esp), *E. oppositifolia* (Eop) and *E. purpurascens* (Epur).

Species Code (See Table 1 and Table 2)			Eplat	Earb-A	Ego	Ebo	EboL	EboB	EarT	Edal	Edal2	EspB	EopO	Epur
Common Name	AI	Pub AI												
α-Thujene	929	924	-	-	-	-	-	-	-	-	-	-	-	3.5
α-Pinene	934	932	1.1	3.9	7.8	1.1	3.0	9.4	-	-	-	20.1	-	13.4
Sabinene	970	969	-	-	2.0	-	-	-	-	-	-	0.9	-	10.8
β-Pinene	975	974	-	-	2.9	2.2	6.8	12.3	-	-	-	10.0	-	-
Myrcene	992	988	-	-	5.4	-	-	0.9	-	-	-	9.4	-	-
p-Cymene	1026	1022	-	-	-	-	-	-	-	-	-	-	-	0.9
Limonene	1028	1024	2.8	-	-	-	-	-	-	-	-	1.4	-	14.6
1,8-Cineole	1034	1026	-	-	-	-	-	-	-	-	-	-	-	50.2
Myodesert-1-ene	1138	NMR	-	-	-	-	-	-	-	18.7	83.2	-	-	-
β-*Cis*-ocimene	1049	1032	0.3	0.8	-	-	-	-	-	-	-	-	-	-
γ-Terpinene	1059	1054	-	-	-	-	-	-	-	-	-	-	-	1.3
Terpinen-4-ol	1180	1174	-	-	-	-	-	-	-	-	-	-	-	0.6
n.d.	1238	-	-	-	-	-	-	-	6.6	-	-	-	-	-
Dihydronepetalactone	1276	-	-	-	-	-	2.3	-	23.1	2.2	-	-	-	-
n.d.	1283	-	-	-	-	-	-	-	11.0	-	-	-	-	-
cis,trans-Iridodial-1	1291	-	-	-	-	-	-	-	-	2.7	1.2	-	-	-
cis,trans-Iridodial-2	1296	-	-	-	-	-	-	-	-	10.3	1.6	-	-	-
p-Vinylguaicol	1311	1309	-	3.9	-	-	-	-	14.2	-	-	-	0.8	-
trans,trans-Iridodial	1314	-	-	-	-	-	-	-	-	13.8	1.0	-	-	-
n.d.	1319	-	-	-	-	-	-	-	16.4	-	-	-	-	-
cis,cis-Nepetalactol	1335	-	-	-	-	-	-	-	-	20.5	-	-	-	-
Cyclohexene,3-butyl-	1370	-	-	-	-	-	-	-	-	1.0	-	-	-	-
α-Copaene	1374	1374	-	-	-	0.8	-	2.3	-	-	-	-	-	-
cis,cis-Nepetalactone	1394	1394	-	-	-	-	-	-	-	7.5	7.7	-	-	-
n.d.	1406	-	-	-	-	-	-	-	9.4	-	-	-	-	-
n.d.	1414	-	-	-	-	-	-	-	7.3	-	-	-	-	-
n.d.	1419	-	-	-	-	0.5	-	1.6	-	-	-	1.1	-	-
*E*-Cinnamic acid	1433	1435	-	14.8	-	-	-	-	0.7	-	-	-	31.0	-
1-Acetoxymyodesert-3-ene	1457	NMR	-	-	-	-	-	-	-	6.8	2.8	-	-	-
Veratraldehyde	1478	-	-	-	-	-	-	-	-	9.7	0.8	-	-	-
β-Copaene	1480	-	-	-	2.6	8.1	3.5	11.2	-	-	-	-	-	-
Germacrene D	1485	1484	-	-	-	8.3	-	-	-	-	-	7.1	-	-
n.d.	1496	-	-	-	-	3.3	11.1	9.5	-	-	-	-	-	-
Germacrene A	1511	1508	0.3	-	-	-	-	-	2.6	-	-	-	-	-
δ-Cadinene	1524	1522	-	-	-	0.4	-	-	-	-	-	-	-	-
n.d.	1575	-	0.4	-	-	6.7	-	1.4	-	-	-	-	-	-
Spathulenol	1579	1577	-	-	-	-	-	-	0.4	-	-	-	-	-
Globulol	1592	1590	-	-	-	-	-	-	-	-	-	-	-	0.6
Benzaldehyde, 3,4,5-trimethoxy-	1601	-	-	-	-	-	-	-	-	3.2	-	-	-	-
10-Epi-7-eudesmol	1621	1622	-	-	-	-	4.6	8.8	-	-	-	-	-	-
*t*-Muurolol	1645	1644	-	-	-	-	-	-	-	-	-	3.6	-	-
α-Eudesmol	1657	1652	-	-	-	-	-	11.1	0.3	-	-	2.0	-	-
β-Eudesmol	1665	1649	-	-	-	-	-	-	-	-	-	6.8	-	-
n.d.	1683	-	-	-	-	-	7.1	3.0	0.3	-	-	-	-	-
9-Hydroxydendrolasin	1740	NMR	-	19.0	-	-	-	-	-	-	-	-	-	-
n.d.	1778	-	-	-	-	0.9	-	1.5	-	-	-	-	-	-
n.d.	1787	-	-	-	-	-	-	5.5	-	-	-	-	-	-
n.d.	1825	-	-	-	-	-	-	-	-	-	-	9.2	-	-
Myoporone	1836	NMR	-	-	-	-	5.8	-	0.4	-	-	-	-	-
n.d.	1865	-	-	-	-	-	-	-	-	-	-	4.6	-	-
n-Hexadecanoic acid	1964	1960	-	1.0	-	1.1	-	-	0.8	-	-	-	0.7	-
Hexadecanoic acid	1995	1994	-	-	-	-	8.8	-	-	-	-	-	-	-
Freelingnite	1987	NMR	-	36.5	-	-	-	-	-	-	-	-	-	-
n.d.	2006	-	-	-	-	30.1	-	-	-	-	-	-	-	-
n.d.	2017	-	-	-	-	3.6	-	-	-	1.2	-	-	-	-
n.d.	2032	-	-	-	-	20.5	-	-	-	-	-	-	-	-
n.d.	2051	-	-	-	-	-	-	-	-	-	-	-	21.6	-
n.d.	2089	-	-	-	-	1.8	4.4	-	-	-	-	5.7	-	-
n.d.	2100	-	1.0	1.1	13.5	5.8	37.3	19.8	1.5	-	-	11.1	1.9	0.6
n.d.	2124	-	-	2.5	5.4	2.5	4.4	-	2.3	1.1	-	4.2	5.4	-
Oppositifolic acid	2149	NMR **	0.3	-	-	-	-	-	-	-	-	-	13.0	-
n.d.	2165	-	-	10.0	-	-	-	-	-	-	-	-	-	-
n.d.	2186	-	14.9	-	-	-	-	-	-	-	-	-	-	-
n.d.	2203	-	14.6	-	-	-	-	-	-	-	-	-	-	-
n.d.	2281	-	-	-	-	-	-	-	-	-	-	-	21.7	-
n.d.	2287	-	11.8	-	-	-	-	-	-	-	-	-	-	-
n.d.	2409	-	-	-	-	-	-	-	0.6	-	-	-	-	-
n.d.	2416	-	-	-	21.1	-	-	-	0.4	-	-	-	-	-
n.d.	2428	-	-	-	35.2	-	-	-	-	-	-	-	-	-
n.d.	2475	-	25.8	-	-	-	-	-	-	-	-	-	-	-
n.d.	2517	-	-	3.4	-	-	-	-	-	-	-	-	-	-
n.d.	2531	-	23.4	-	-	-	-	-	-	-	-	-	-	-

AI, arithmetic index; Pub. AI, published arithmetic index; ** Published as 5-acetoxymethyltetradeca-trans-2,trans-4,trans-6-trienoic acid [48].

**Table 11:** Chemistry of solvent extract volatiles; *Eremophila pterocarpa* (Epte), *E. veronica* (Ever), *E. arbuscular* (Earb), *E. laanii* (Elaa), *E. hillii* (Ehil), *E. purpurascens* (Epur), *E. paisleyi* (Epai), *E. santalina* (Esan), *E. weldii* (Ewel), *E. recurva* (Erec), and *E. pustulata* (Epus).

Species Code (See Table 1 and Table 2)			*Epte*	*Ever*	*Earb-B*	*Elaa*	*Ehil*	*Epur2*	*Epai*	*Esan*	*Ewel*	*Erec*	*Epus*
Common Name	AI	Pub. AI											
α-Thujene	929	924	-	-	-	-	-	6.6	-	-	-	-	-
α-Pinene	934	932	-	5.3	6.2	4.2	-	20.2	-	0.9	67.9	-	3.8
Sabinene	976	969	-	-	-	-	-	15.0	-	-	-	-	76.5
β-Pinene	981	974	-	2.0	-	22.5	-	-	-	6.2	-	-	-
α-Phellandrene	1010	1002	-	7.6	-	-	-	-	-	-	-	-	-
β-Phellandrene	1026	1025	-	17.5	-	-	-	16.0	-	-	9.5	-	19.7
1,8-Cineole	1031	1024	-	-	-	-	-	42.2	-	-	-	-	-
Methoxymyodesert-3-ene	1289	NMR	-	-	-	-	-	-	-	91.3	-	-	-
2-Methoxy-4-vinylphenol	1322	1315	-	-	-	-	31.4	-	-	-	6.4	-	-
cis,cis-Nepetalactone	1395	1391	-	-	-	-	-	-	-	1.6	-	-	-
Alloaromadendrene	1454	1458	-	-	-	-	18.8	-	-	-	-	3.5	-
Germacrene D	1500	1488	-	-	-	-	-	-	-	-	-	17.1	-
Bicyclogermacrene	1512	1500	-	9.1	-	-	-	-	-	-	-	7.6	-
n.d.	1529	-	-	-	-	-	-	-	-	-	-	10.1	-
n.d.	1533	-	-	-	-	-	-	-	-	-	-	6.1	-
Elemol	1553	1548	-	-	-	-	6.5	-	80.3	-	-	-	-
Germacrene D-4-ol	1574	1576	-	-	-	-	-	-	-	-	-	26.9	-
Spathulenol	1577	1577	-	-	-	-	-	-	-	-	3.9	-	-
n.d. furanosesquiterpene	1592	-	-	-	-	41.5	-	-	-	-	-	-	-
γ-Eudesmol	1627	1630	-	-	-	-	-	-	4.5	-	-	-	-
Eremoacetal	1630	NMR	-	33.3	-	-	-	-	-	-	-	14.1	-
*t*-Muurolol	1635	1640	-	-	-	-	-	-	-	-	3.9	-	-
Ngaione	1639	NMR	4.1	-	-	4.3	-	-	-	-	-	-	-
n.d. furanosesquiterpene	1654	-	-	-	-	13.1	-	-	-	-	-	-	-
Dehydrongaione	1677	NMR	8.5	2.7	-	11.3	-	-	-	-	-	-	-
Ngaiol ketol *	1699	**	-	17.8	-	3.1	-	-	-	-	-	-	-
n.d.	1708	-	-	-	-	-	-	-	4.1	-	3.6	-	-
9-Hydroxydendrolasin	1740	NMR	-	-	8.2	-	-	-	4.1	-	4.8	-	-
n.d. furanosesquiterpene	1771	-	4.1	-	-	-	-	-	7.0	-	-	-	-
n.d. furanosesquiterpene	1814	-	-	-	-	-	-	-	-	-	-	5.6	-
Myoporone	1840	NMR	-	4.7	-	-	-	-	-	-	-	9.0	-
Dehymyoporone	1901	NMR	11.1	-	-	-	-	-	-	-	-	-	-
Phytol	1928	1930	-	-	-	-	36.7	-	-	-	-	-	-
n.d. furanosesquiterpene	1912	-	3.6	-	-	-	-	-	-	-	-	-	-
n.d. furanosesquiterpene	1932	-	49.1	-	-	-	-	-	-	-	-	-	-
n.d. furanosesquiterpene	1935	-	10.0	-	-	-	-	-	-	-	-	-	-
n.d. furanosesquiterpene	1944	-	9.5	-	-	-	6.6	-	-	-	-	-	-
Freelingnite	1987	NMR	-	-	85.6	-	-	-	-	-	-	-	-

AI, arithmetic index; Pub. AI, published arithmetic index; * likely to be 4-hydroxyngaione [28]; **, no data found; n.d., not determined; NMR, determined by nuclear magnetic resonance spectroscopy.

**Table 12:** Solvent extract volatiles from *Eremophila mitchellii* (Emi), *E. gilesii* (Egi), *E. oppositifolia* (Eop) subsp. *rubra* (EopR) and subsp. *oppositifolia* (EopR).

Species Code (See Table 1 and Table 2)			Emi-436	Egi-518	EopR-540	EopO-538 B	EopR-535	EopR-539
Common Name	AI	Pub AI						
α-Thujene	928	924	-	-	-	1.0	-	-
α-pinene	933	932	28.6	9.2	-	-	1.5	-
2E-Heptenal	941	947	-	0.6	0.9	0.6	1.6	2.8
Camphene	946	946	-	-	-	0.6	-	1.4
β-Pinene	975	974	-	0.5	-	-	-	-
Myrcene	992	988	-	9.2	-	-	-	-
Butyl butanoate	994	993	-	-	-	5.5	-	-
n-Decane	997	1000	-	-	-	1.5	-	-
α-Phellandrene	1005	1002	1.7	0.6	-	0.6	-	-
α-Terpinene	1019	1014	-	-	1.3	-	1.2	0.8
β-Phellandrene	1025	1025	0.9	-	-	-	-	-
p-Cymene	1026	1022	-	0.5	-	-	-	-
Limonene	1028	1024	-	1.5	-	-	-	-
β-Ocimene	1048	1044	-	0.6	-	0.8	-	-
n-Undecane	1097	1100	-	-	-	1.0	-	-
Karahanaenone	1154	1154	-	2.4	-	-	-	-
Isomenthone	1162	1158	-	0.7	-	-	-	-
Hexyl butyrate	1190	1191	-	-	-	4.8	-	-
p-Vinylguaiacol	1313	1309	4.9	-	-	-	-	-
δ-Elemene	1337	1335	1.0	-	-	-	-	-
α-Copaene	1375	1374	-	-	-	-	1.2	-
β-Elemene	1396	1389	-	1.6	-	-	-	-
β-Duprezianene	1418	1421	-	0.6	0.8	-	-	-
*E*-Caryophyllene	1425	1417	-	-	-	1.3	1.4	-
1-Amorpha-4,7(ll)-diene	1480	1479	-	1.7	-	-	-	-
*Z*-β-Guaiene	1485	1492	-	0.5	-	-	-	-
Bicydogermacrene	1502	1500	18.8	4.4	-	-	1.5	-
Germacrene A	1511	1508	-	1.7	-	-	-	-
δ-Cadinene	1522	1522	-	2.3	-	0.7	3.1	-
8,14-Cedranoxide	1544	1541	-	-	-	0.7	-	-
n.d.	1568	-	-	0.8	-	-	-	-
Spathulenol	1579	1577	3.5	0.7	-	0.7	-	-
n.d.	1584	-	-	1.2	-	-	-	-
Humulene epoxide II	1611	1608	-	-	-	1.2	-	-
*t*-Muurolol	1645	1644	-	-	-	-	1.3	1.1
β-Eudesmol	1653	1649	1.6	-	-	-	-	-
*Z*-11-Hydroxyisodendrolasin	1678	NMR	-	-	-	2.6	-	-
Epignaione	1694	NMR	-	-	-	1.0	-	-
9-Hydroxydendrolasin	1741	NMR	-	-	-	0.7	-	-
10R-Hydroxydihydro-α-humulene acetate	1755	NMR	-	-	-	3.1	-	-
8-Cedren-13-ol acetate	1788	1788	-	-	1.1	-	1.0	0.5
n.d.	1895	-	-	-	2.3	-	-	0.6
n.d.	2100	-	5.4	-	-	-	-	-
Oppositifolic acid	2149	NMR **	-	-	69.2	69.8	84.6	56.2
n.d.	2260	-	-	-	-	-	-	0.7
n.d.	2269	-	-	-	-	-	-	2.2
n.d.	2270	-	-	11.7	-	-	-	
n.d. chain 1	2272	-	-	-	10.0	-	-	0.7
n.d.	2318	-	15.6	-	-	-	-	-
n.d. chain 2	2359	-	-	-	12.7	-	-	30.7
n.d. isomer 2	2412	-	-	46.8	-	-	-	-
n.d.	2449	-	6.7	-	-	-	-	-
n.d.	2471	-	11.2	-	-	-	-	-

AI, arithmetic index; Pub. AI, published arithmetic index; ** Previously published under another name as 5-acetoxymethyltetradeca-trans-2,trans-4,trans-6-trienoic acid [48].

However, this pure fraction of volatiles in Table 4 tentatively demonstrates a pronounced chemical difference between the two subspecies. Due to a small sampling size, it is not known whether this relationship is robust. Nevertheless, EopR (subsp. *rubra*) yielded *t*-muurolol, which was confirmed by NMR. Solvent-extracted specimens also had *t*-muurolol in their chemical profile (Table 12). In contrast, EopO (subsp. *oppositifolia*) is characterised by caryophyllene, caryophyllene oxide and a new metabolite that we assigned as 10-hydroxydihydro-α-humulene acetate. The NMR spectra for this new compound (Supplementary A2) was identical to the spectra provided by Mosaddik and Waterman [49], but the determined structures differ by the position of the acetate moiety. Mosaddik and Waterman [49] reported three HMBC correlations from the acetate position to the quaternary carbon or attached methyl carbon, which were not observed by us. Hence, the reason for the similarity of ^1^H and ^13^C NMR spectra is not known. 

Simionatto et al. previously described the isolation of the corresponding non-acetylated structure, i.e., 10-hydroxydihydro-α-humulene, from *Zanthoxylium hiemale* [50]. The absolute configuration of the secondary alcohol at C-10 was determined as (*R*) based on Horeau’s method. In the current study the enantiomer of 10-hydroxydihydro-α-humulene acetate was not tested.

### 2.4. Miscellaneous Eremophila by Endemic Location

Several other species were sampled only 1–3 times for the current study. Table 4, Table 10, Table 11 and Table 12 contain data for these individuals. Table 4 includes data for essential oils, whereas Table 10, Table 11 and Table 12 include data for solvent extracts of leaves. Data in Table 11 were produced in a separate study and are included here serendipitously. These data were produced using a shorter chromatographic method, so the compounds with lower vapour pressures were not detected. The botanical material used to produce the data in Table 11 was sourced from the Australian Arid Lands Botanic Garden in Port Augusta, but the endemic locations of most species are restricted, with general locations listed in Table 1. More details of endemic locations of species can be garnished from the Australasian Virtual Herbarium website [51].

#### 2.4.1. Central and South Australian *Eremophila*

In traveling north from Port Augusta into the Northern Territory to meet Alice Springs, the vegetative communities change moderately. In particular, the endemic distribution of several species is restricted to South Australia, extending only several hundred kilometres north of Port Augusta and Adelaide. This is true for *E. santalina* (Esan), a species with a slight morphological resemblance to *E. deserti*, and according to the current chemical study, a strong chemical relationship. This species produced >90% methoxymyodesert-3-ene in its volatiles profile (Table 11) and yielded at >1% g.g^−1^ of wet leaf weight, which is like *E. deserti*.

In contrast, *E. weldii* (Ewel) expressed nearly 70% α-pinene, which is a chemical feature familiar to other species of *Eremophila*. Furthermore, 9-hydroxydendrolasin was tentatively identified as a minor component in *E. weldii*, as well as *E. paisleyi* (Epai). This furanosesquiterpene was characterised as a major component in hydrodistilled essential oil from *E. arbuscular* as mentioned earlier and reiterated in Table 11 as a minor component due to the high yield of freelingnite. An unidentified furanosesquiterpene was also detected in the profile from *E. hillii*, which also included alloaromadendrene, which is unusual for species of *Eremophila*, and a phenol (2-methoxy,4-vinyl-phenol), which is generally associated with species that produce lignans. This was not investigated in the current study.

However, it is of interest that *E. paisleyi* expressed > 80% elemol in its volatiles profile. This is because it was previously speculated that elemol was associated with inhibition of *Sarcoptes scabiei* by using extracts from *E. dalyana* [3]. These are two out of three species known for the treatment of scabies itch mite in traditional Australian medicine [20,21]. The third is *E. duttonii*, but this species is widely distributed, and the geographical specificity of therapeutic use has not been clearly elucidated. The specimen of *E. duttonii* of the current study did not express elemol (Table 4).

A species that was sampled from the plains surrounding Uluru (NT), *E. gilesii*, was examined as an essential oil (Egi-341; Table 4) and as a solvent extract (Egi-518; Table 12). The essential oil is predominantly made of α-pinene, myrcene, and limonene, but one specimen also expressed traces of karahanaenone. The solvent-extracted specimen in Table 12 demonstrates unidentified larger mass compounds in the chromatogram that were not studied further in the current study. 

#### 2.4.2. *Eremophila* of the Murchison District, Western Australia

The Murchison District of Western Australia is potentially the location of the ancestral stock of Australian *Eremophila*. It was put forward by Barlow that post glaciation aridity more than 75K years prior caused a significant die back of Australian species, restricting the *Eremophila* genus to the Murchison District, from where it developed polyploidy and increased adaptability to recolonise the lands of the continent [9]. It was later speculated that Mutawintji NP of New South Wales may have also been an ancestral stock, since most of the tetraploid *E. longifolia* of Australia follow terpenoid biosynthesis, which is a character of the diploids of Mutawintji NP, but not the Murchison diploids, which express only phenylpropanoids [2]. 

A species in the Murchison District of Western Australia that is very similar to *E. gilesii* is *E. foliosissima*, which was collected from the Sandstone airstrip. *Eremophila foliosissima* produced an essential oil (Efo-57, Table 4) with a different profile of volatiles compared to *E. gilesii*, comprised by the monoterpenoid pinene isomers (α/β) and the sesquiterpenes, elemol and eudesmol isomers. 

Three further essential oils in Table 4 were produced from *E. platycalyx* (Epla-53, Epla-61, and Epla-62), which were sampled from the Murchison District of Western Australia and identified by Robert Chinnock (authority on *Eremophila* [1]) in email correspondence. It was communicated that *E. platycalyx* is morphologically variable and may include variants that are not yet described. 

The monoterpenes in the hydrodistilled essential oils from *E. platycalyx* include the pinene isomers, camphene, limonene and β-phellandrene (Table 4). Furthermore, like the sympatric species *E. foliosissima*, *E. platycalyx* also expresses the eudesmol isomers. Lastly, farnesol is also evident in the essential oil profile, which as previously mentioned is a promoter of biofilm formation in bacteria. *Eremophila platycalyx*, was also studied by solvent extraction (Epla, Table 10) from a private garden specimen. The analysis of the solvent extract (Epla, Table 10) detected > 90% low vapour pressure components that are not driven into the essential oil in hydrodistillation. These components were not isolated or identified in the current study. 

A species that occurs in the Murchison District but is also widely distributed across Australia is *E. youngii* (Eyo-345). This species gives a high-yielding monoterpenoid essential oil with 1,8-cineole as a major component. However, since this species has such a wide distribution it should be examined for cytogeographical and chemotypic variation, like *E. longifolia*, because it occurs in the Murchison District. 

Two of the species from the Murchison District expressed ngaione, dehydrongaione and significant relative abundances of unidentified furanosesquiterpenes that require isolation and elucidation in a future study. These two species were *E. laanii* (Elaa) and *E. pterocarpa* (Epte). As a side note, the two non-volatile furofuran lignans sesamin and aptosimon were also isolated from leaves of *E. pterocarpa*, and assigned against published spectra [52,53]. Then, another two species expressed germacrene D, i.e., *E. spectabilis* subsp. *brevis* (EspB) and *E. recurva* (Erec) (Table 10 and Table 11, respectively). However, *E. recurva* also expressed the uncommon sesquiterpene germacrene-D-4-ol, as well as eremoacetal. Eremoacetal was identified in only one other species, which is also endemic to Western Australia but restricted to southern parts, proximate to Kalgoorlie. 

#### 2.4.3. South-Western Australian *Eremophila* (Kalgoorlie Region)

The Kalgoorlie region of Western Australia consists of volcanic and sedimentary rocks and their derived soils that make up the Norseman-Wiluna Belt, a greenstone belt of the Archaean period from between 2.6 and 2.9 billion of years ago. These soils were devoid of *Eremophila* until recolonised by tetraploids after the extensive die-back, which is postulated to have occurred approximately 75K years ago as previously mentioned. In the current study, four of the species are endemic to this location, which include *E. arachnoides* subsp. *tenera*, *E. purpurascens*, *E. veronica* and *E. pustulata*. 

*Eremophila veronica* (Ever) is the other species that expressed eremoacetal in its chemical profile (Table 11), which yielded >1% g.g^−1^ according to qNMR. This species also expressed a tentatively identified ketol, which we have called ‘ngaiol’, assigned by its mass spectrum only, which could be 4-hydroxyngaione as identified in *E. duttonii* in an earlier study [28]. This component requires proper structural assignment. However, previous studies that have produced it by synthesis noted that it is unstable and degrades very quickly [54], which is consistent with the outcome of the current study. 

Like *E. youngii*, *E. purpurascens* (Epur) yielded a monoterpenoid essential oil comprised by common components, with the major component 1,8-cineole at >50% (Table 10), followed by α-pinene, sabinene and β-phellandrene. This outcome was confirmed by a second analysis of material from a private garden (Epur2), with 1,8-cineole at >42% (Table 11). Another monoterpenoid essential oil was derived from *E. pustulata* (Epus; Table 11) but with sabinene as the major component at a relatively concentration of >76%. In contrast, *E. arachnoides* subsp. *tenera* (EarT; Table 10) yielded an unusual essential oil with several unidentified components, and an iridoid dihydronepetalactone, tentatively assigned by mass spectral data. This latter species represents a promising candidate for the identification of previously undescribed compounds.

#### 2.4.4. Central Lowlands Region

The central lowlands region contains Mutawintji NP of New South Wales, and the ‘Grey Ranges’ north of Thargomindah, Queensland. This region provides habitat for several of the species previously mentioned in the current study, including *E. dalyana*, *E. sturtii*, *E. mitchellii*, *E. arbuscular*, *E. oppositifolia*, *E. alternifolia* and *E. latrobei*, just to name a few. Several other species occurring in this region are also distributed widely across the continent, such as *E. longifolia*. 

Two additional species were sampled from a private garden, which include three variants of *E. bowmannii* (Ebo), and one specimen of *E. goodwinii* (Ego) (Table 10), which is endemic to both central Australian and Queensland disjunct locations. Three variants of *E. bowmannii* were sampled from a private garden, which were *E. bowmannii* (Ebo), *E. bowmannii* subsp. *latifolia* (EboL) and *E. bowmannii* subsp. *bowmannii* (EboB). While common components such as the cadinene (α/β/δ) and pinene (α/β) isomers were detected (Table 10), a significant number of unidentified constituents were detected in all three subspecies, but they were distinguished clearly by signatory components. Ebo included unidentified components with RI values from 2006 to 2032, EboL included myoporone and EboB included α-eudesmol (Table 10). These subspecies require further research for identification of unidentified components and to explore the chemical differences at the subspecies level. 

The one specimen of *E. goodwinii* (Ego) also expressed several unidentified components and is yet another species of the current study that is a source of previously undescribed natural products. However, these unidentified components will not be present in essential oils, due to low vapour pressures. A mere hydrodistilled essential oil from this species will yield a monoterpenoid composition dominated by pinene isomers (α/β), sabinene and myrcene and traces of β-copaene. A similar scenario is expected for *E. bowmannii* and subspecies. 

## 3. Materials and Methods

### 3.1. Specimen Collection

The locations of field specimens of all taxa and names are summarised in Table 1 (for solvent-extracted material) and Table 2 (for hydrodistilled material). All analyses were performed on leaves, except for a single wood essential oil of *E. mitchellii* (noted in text). Field specimens were collected in the years ranging from 2012 to 2019 and vouchers were lodged at the N.C.W Beadle Herbarium at the University of New England, Armidale, NSW Australia. Voucher references are N.J. Sadgrove XXX, where XXX refers to the authors collection number (i.e., 533 = N.J. Sadgrove 533). In the current study, only the numbers are used throughout the text and in the tables.

### 3.2. Production of Essential Oils

Essential oils were produced using hydrodistillation. Approximately 600 g of fresh leaf was removed from the twig then cut into 0.5 mm fragments and placed into a 5 L round-bottom flask with 2.5 L of deionised distilled water (ddH_2_O). Leaves were heated for 3–4 h using continuous cohobation of hydrosol [55]. Afterwards the steam/oil mix was collected from the 1000 mL separating funnel after separating from the hydrosol. Essential oils were stored away from light at 4 °C until used.

### 3.3. Chromatography Methods and Compound Identification (GC–MS and NMR Analysis)

In several cases hydrodistilled essential oils were studied. Prior to GC–MS analysis the essential oils were dried to remove hydrosol emulsions using anhydrous sodium sulphate (Na_2_SO_4_) powder (0.5 g in 10 mL essential oil) for at least 24 h. Afterwards, essential oils were dissolved in dichloromethane (CH_2_Cl_2_) at a ratio of 1:1000. If a single leaf extraction was performed, a small fragment of leaf, approximately 100-500 mg, was extracted with CH_2_Cl_2_ for analysis by GC–MS.

Analyses were performed using a HP 6890 gas chromatograph coupled with a HP 5973 mass spectrometer detector. An autosampler unit (HP 7673–100 positions) was used to perform the 1 μL injections. Separation was accomplished with a HP-5MS column (30 m by 0.25 mm, i.d., 0.25 μm phase thickness). Operating conditions were as follows: injector: split ratio 25:1; temperature: 250 °C; carrier gas: helium, 1.0 mL/min, constant flow; column temperature, 60 °C (no hold), 5 °C per minute then @ 280 °C hold for 4 min. A second method was used for solvent extracts, 50 °C (no hold), 5 °C per minute then @ 280 °C hold for 20 min. MS was acquired at 70 eV using a mass scan range of 45–400 *m*/*z*. Quantification was based on the peak areas on the chromatogram generated by MS data (total ion chromatogram). Integration parameters were set to exclude peak areas less than 0.5%.

**Figure 9:**
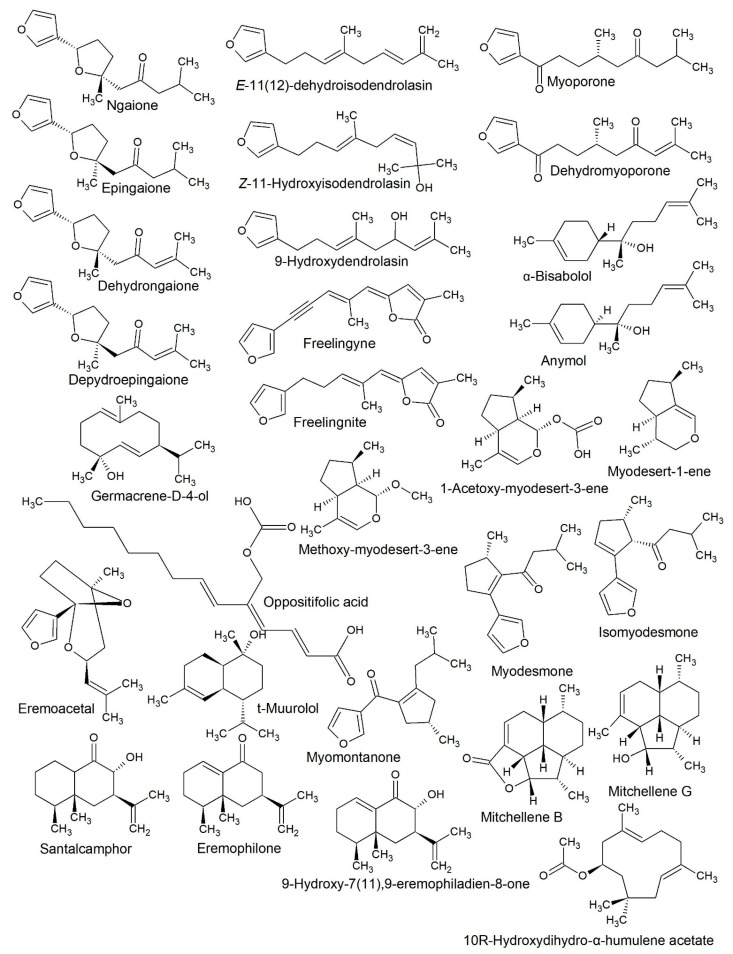
Uncommon or new sesquiterpenes isolated from species of Eremophila and assigned in the current study.

Most identification of volatile organic compounds was performed by comparison of mass spectra with the NIST electronic library database [56] and confirmed using calculated retention indices relative to n-alkanes, then compared to published values. A second library in the form of a book by Adams [57] was used to resolve any further discrepancies with identification. Most samples were injected once only, accept in cases of unusual or unexpected chemical profiles. Several of the components were identified by isolation (10% ethyl acetate, 90% hexane, normal phase column chromatography using silica gel) and comparison of respective ^13^C NMR spectra to published values, using a 500 MHz Bruker Avance (Germany) spectrometer in *d*-chloroform. These were: anymol [58], *t*-muurolol [59], freelingnite [60], ngaione [13], epingaione and dehydrongaione and dehydroepingaione [15], myoporone and dehydromyoporone [18], myodesmone and isomyodesmone [61], myomontanone [62], eremoacetal [63], methoxymyodesert-3-ene [12], 1S-acetoxymyodesert-3-ene [16], myodesert-1-ene [3], mitchellenes G and B [4], 9-hydroxydendrolasin (listed as 6-hydroxydendrolasin in literature) [46], oppositifolic acid (as 5-acetoxymethyltetradeca-trans-2,trans-4,trans-6-trienoic acid) [48], germacrene-D-4-ol (listed as 1,6-germacradien-5-ol) [64], eremophilone and santalcamphor and 9-hydroxy-7(11),9-eremophiladien-8-one [7]. These components (Figure 9) were authenticated by matching ^1^H or ^13^C NMR spectra to published values (citations given in brackets). See Supplementary A2 and B for NMR spectra; see Supplementary A1 and A4 for MS data; for images of compounds, see Supplementary A3 for more structures or Figure 9 for the most relevant to the current study. 

### 3.4. Multivariate Analysis

Some of the multivariate analysis (Section 2.4) included a previously published dataset for *E. longifolia*, which was combined with the current dataset for the same species, and an updated dataset previously available from Supplementary files of *E. sturtii*. Details of the locations of collection from the former datasets are available from the Supplementary files of Sadgrove and Jones [2] and Sadgrove et al. [4].

Percentage compositions of the essential oil components were used as input data to perform a hierarchical clustering analysis (HCAbp) with bootstrap resampling in the software R 3.4.1 (R Foundation for Statistical Computing, Vienna, Austria), using the pvclust [65] package, the ward’s clustering algorithm and Euclidean distance. Prior to multivariate analyses, raw data were scaled by the arcsine method in accordance with previous reports for data expressed as percentages [66,67]. Two types of support values were plotted in the HCAbp using 10,000 replicates: traditional bootstrapping (bp) and approximately unbiased (au) *p*-values.

A principal component analysis (PCA) was performed using the prcomp function and the factoextra package in the software R. Prior to PCA, raw data were scaled by the arcsine method in accordance with previous reports for data expressed as percentages [66,67].

## 4. Conclusions

Many species of *Eremophila* will yield common essential oil components in hydrodistillation, but the low vapour pressure ingredients detected in GC–MS analysis can only be recognised by solvent extraction and direct injection and would otherwise not be recognised if studied as an essential oil. Low vapour pressure volatiles are lipophilic and easily isolated using routine flash chromatography over silica (normal phase). *Eremophila* is evidently a source of high-yielding rare metabolites that could be utilised in industry as synthetic precursors or for cosmetics.

In the current study, it is demonstrated that the chemical geography of individual species is an important factor in understanding chemical diversity. However, it is just as common to see apparently random phenoplasticity. Furthermore, there is tentative evidence that variants or subspecies may also predict specific chemical profiles in some species, but this requires larger datasets for confirmation.

## Figures and Tables

**Table 1:** Species of *Eremophila* that were studied by solvent extraction of leaves and direct injection into the GC–MS.

Species	Abr.	Location	Species	Abr.	Location
E. pterocarpa *	Epte	Murchison, WA	*E. goodwinnii*	Ego	Private Garden—Inverell, NSW
E. veronica *	Ever	Kalgoorlie, WA	*E. spectabilis* subsp. *brevis*	EspB	Private Garden—Inverell, NSW
E. laanii *	Elaa	Murchison, WA	*E. purpurascens*	Epur2	Private Garden—Inverell, NSW
E. hillii *	Ehil	SA-WA border, Great Australian Bight	*E. bowmannii*	Ebo	Private Garden—Inverell, NSW
E. purpurascens *	Epur	Kalgoorlie, WA	*E. bowmannii* subsp. *bowmannii*	EboB	Private Garden—Inverell, NSW
E. paisleyi *	Epai	Alice Springs – Port Augusta	*E. bowmannii* subsp. *latifolia*	EboL	Private Garden—Inverell, NSW
E. santalina *	Esan	Port Augusta, SA	*E. arachnoides* subsp. *tenera*	EarT	Private Garden—Inverell, NSW
E. weldii *	Ewel	SA-WA border, Great Australian Bight	*E. dalyana*	Edal	Private Garden—Inverell, NSW
E. recurva *	Erec	Gascoyne Junction, near Murchison, WA	*E. dalyana*	Edal2	Private Garden—Inverell, NSW
E. pustulata *	Epus	Kalgoorlie, WA	*E. arbuscular*	Earb	Private Garden—Inverell, NSW
*E. platycalyx*	Epla	Private Garden—Inverell, NSW	*E. arbuscular*	Earb2	Private Garden—Inverell, NSW
*E. oppositifolia* subsp. *oppositifolia*	EopO	Private Garden—Inverell, NSW	*E. freelingii*	Efre-338A	Alice Springs, NT
*E. oppositifolia* subsp. *rubra*	EopR-535	Broken Hill to Wiilcannia, NSW	*E. freelingii*	Efre-338B	Alice Springs, NT
*E. oppositifolia* subsp. *rubra*	EopR-538B	Broken Hill to Wiilcannia, NSW	*E. freelingii*	Efre-338C	Alice Springs, NT
*E. oppositifolia* subsp. *rubra*	EopR-539	Broken Hill to Wiilcannia, NSW	*E. freelingii*	Efre-338D	Alice Springs, NT
*E. oppositifolia* subsp. *rubra*	EopR-540	Broken Hill to Wiilcannia, NSW	*E. alternifolia* X *Myoporum montanum*	EaltMm	Private Garden—Inverell
*E. gilesii*	Egil-518	SW Qld	*E. alternifolia*	Ealt-A	Private Garden—Inverell
*E. latrobei* subsp. *latrobei*	ElatL	Private Garden—Inverell, NSW	*E. alternifolia*	Ealt-B	Private Garden—Inverell
*E. latrobei* subsp. *glabra*	ElatG	Private Garden—Inverell, NSW	*E. alternifolia*	Ealt-C	Private Garden—Inverell
*E. latrobei* subsp. *filiform*	ElatF	Private Garden—Inverell, NSW	*E. alternifolia* subsp. *latifolia*	EaltL-A	Private Garden—Inverell
*E. deserti*	Ede	Private Garden—Inverell, NSW	*E. alternifolia* subsp. *latifolia*	EaltL-B	Private Garden—Inverell
*E. mitchellii*	Emi-436	SE Qld	*E. alternifolia* subsp. *latifolia*	EaltL-C	Private Garden—Inverell

* Cultivated at Australian Arid Lands Botanic Garden of Port Augusta, South Australia. Location indicates endemic location.

**Table 2:** Species of *Eremophila* that were studied by hydrodistillation and chemical analysis of essential oils. All were from leaves except for Emi-Wood Oil.

Species	Collector No.	Location	Species	Collector No.	Location
*E. gilesii* (Egi)	341	Alice Springs, NT	*E. alternifolia* (Ealt)	170	Broken Hill, NSW
*E. platycalyx* (Epla)	53	Sandstone, WA		261	Broken Hill, NSW
	61	Mt Magnet, WA		408	Broken Hill, NSW
	62	Mt Magnet, WA	*E. longifolia* (Elo)	517	Cunnamulla, Qld
*E. deserti* (Ede)	105	Emerald, Qld		521	Cunnamulla, Qld
	470	Blackall, Qld			
	471	Blackall, Qld		522	Grey Ranges, Thargomindah, Qld
	494A	Miles, Qld		524	Cunnamulla, Qld
	494B	Miles, Qld		479	Blackall, Qld
	494C	Miles, Qld		488	Blackall, Qld
	507B	Goondawindi, Qld	*E. neglecta* (Ene)	Private Land	SA border to NT
	509-May *	Moonie, SW Qld	*E. arbuscular* (Earb)	487	Idalia, NP, Qld
	510-Oct *	Moonie, SW Qld		486	Idalia, NP, Qld
	519A	Cunnamulla, Qld	*E. oppositifolia* subsp. *rubra* (EopR) and subsp. oppositifolia (EopO)	EopR-535B	Wilcannia, NSW
	519B	Cunnamulla, Qld		EopO-538A	Broken Hill, NSW
	519C	Cunnamulla, Qld	*E. sturtii* (Est)	533	Cobar, NSW
	519E	Cunnamulla, Qld		534	Cobar, NSW
	536A	Broken Hill, NSW		537	Wilcannia, NSW
	536B	Broken Hill, NSW		DL-21 **	Broken Hill, NSW
	536C	Broken Hill, NSW		173	Mutawintji NP, NSW
*E. mitchellii* (Emi)	93	North Star, NSW		DL-25 **	Mutawintji NP, NSW
				410	Mutawintji NP, NSW
	181	Warren, NSW		520	Cunnamulla, Qld
	436	Collarenebri, NSW	*E. latrobei* (Elat)	269	Mutawintji NP, NSW
	541	Wilcannia, NSW		337	Coober Pedy, NT
	Emi-Wood EO	Private Collection	*E. youngii* (Eyo)	345	Alice Springs, NT
*E. foliosissima* (Efo)	57	Sandstone, WA	*E. freelingii* (Efre)	346	Alice Springs, NT
*E. duttonii* (Edut)	DL-27 **	Mutawintji NP NSW	**-	-	-

* Same specimen at different times of the year. ** Collector reference is D. Lyddiard 21 or D. Lyddiard 25.

## Supplementary Materials

The following are available online at https://www.mdpi.com/article/10.3390/plants10040785/s1, Supplementary files A1–A4 and Supplementary B. Supplementary files include mass spectral data (Supplementary A1 and A4), ^13^C and ^1^H NMR tables (Supplementary A2), ^13^C and ^1^H NMR images (Supplementary B), and images of all rare compounds mentioned throughout the text (Supplementary A3).
