# Supplementary material for: The Diversity of Volatile Compounds in Australia’s Semi-Desert Genus Eremophila (Scrophulariaceae)
Source: Plants (Basel). 2021 Apr 16;10(4):785. doi: 10.3390/plants10040785 (PMC8073941; doi:10.3390/plants10040785)

Supplementary Files A: The diversity of volatile compounds in Australia's semi-desert genus *Eremophila* (Scrophulariaceae)

Nicholas J. Sadgrove [1,*], Guillermo F. Paddilla-González[1], Alison Green[1], Moses K. Langat[1], Eduard Mas-Claret[1], Dane Lyddiard[2], Julian Klepp[2], Sarah V.A.-M. Legendre[2], Ben W. Greatrex[2], Graham L. Jones[2], Iskandar M. Ramli[2], Olga Leuner[3], and Eloy Fernandez-Cusimamani[3,*]

[1] Jodrell Science Laboratory, Royal Botanic Gardens Kew, Richmond TW9 3DS, UK; f.padilla@kew.org (G.F.P.-G.), m.langat@kew.org (M.K.L.)
[2] School of Science and Technology and School of Rural Medicine, University of New England, Armidale NSW 2351, Australia; bgreatre@une.edu.au (B.W.G.), gjones2@une.edu.au (G.L.J.)
[3] Department of Crop Sciences and Agroforestry, Faculty of Tropical AgriSciences, Czech University of Life Sciences Prague, Kamýcká 129, 16500 Prague, Czech Republic
* Correspondence: n.sadgrove@kew.org (N.J.S.); eloy@ftz.czu.cz (E.F.-C.); Tel.: +44-7857569823 (N.J.S.);
+420-224382183 (E.F.-C.)

Document Structure

Supplementary File A1 – EI Mass spectral data (Table and images)

Supplementary File A2 – Full NMR shift values (tables only)

Supplementary File A3 – Structures (images)

Supplementary File A4 – EI mass spectral data (images) of selected unknown compounds

Supplementary Files B – 1H and 13C NMR images of rare or new compounds from species of *Eremophila* (Scrophulariaceae)

Supplementary Files – A1; EI Mass spectral data (Table and images)

Table A1-1 - Mass spectral data for electron impact ionisation of rare compounds

Name	AI	RT	Electron Impact Mass Spectrum of compounds, determined using GCMS
Myodesert-1-ene	1141	9.39	m/z(rel.int.): 152 (M^+, 76), 137 (100), 122 (19), 110 (46), 95 (60), 81(70), 67(50), 53 (15), 41(22)
Methoxymyodesert-3-ene	1284	13.1	m/z(rel.int.): 182 (M^+, 80), 151 (27), 135 (67), 108 (38), 98 (76), 85(100), 55(26) 41 (23)
Cis, Cis – Nepetalactone	1394	17.91	m/z(rel.int.): 214 (M^+, 1), 166 (98), 123 (100), 109 (49), 95 (65), 81 (75), 69(63), 67 (39), 41 (34)
1-Acetoxymyodesert-3-ene	1459	17.52	m/z(rel.int.): 210 (M^+, 32), 168 (29), 150 (52), 135 (83), 122 (64), 108 (100), 43 (59)
Z-11,12-Dehydroisodendrolasin	1597	20.8	m/z(rel.int.): 200 (M^+, 9), 135 (24), 119 (19), 107 (51), 93 (86), 81 (100), 55(94), 41(60)
Eremoacetal	1630	13.63	m/z(rel.int.): 248 (M^+, 8), 121 (59), 95 (100)
Myomontanone	1646	21.9	m/z(rel.int.): 232 (M^+, 18), 217 (85), 203 (60), 189 (42), 161 (100), 147 (15), 95 (94) 39 (15)
Isomyodesmone	1649	21.97	m/z(rel.int.): 232 (M^+, 38), 175 (100)
Myodesmone	1650	21.98	m/z(rel.int.): 232 (M^+, 52), 175 (100), 161 (8), 147 (9), 105 (9), 91 (9)
Anymol	1668	22.76	m/z(rel.int.): 222 (M^+, 1), 204 (35), 189 (9), 175 (4), 161 (22), 147 (9), 134 (13), 119 (81) 109 (72), 93 (100), 79 (33), 69 (67), 55 (23), 41(44)
Z-11-Hydroxydendrolasin	1677	22.6	m/z(rel.int.): 216 (M^+, 14), 201 (27), 161 (17), 135 (71), 119 (32), 107 (72), 93(100), 81 (90), 55 (41), 43 (39)
Ngaione	1687	22.82	m/z(rel.int.): 250 (M^+, 8), 151 (100), 110 (26), 85 (80), 57 (44), 43 (47)
Epignaione	1694	22.98	m/z(rel.int.): 250 (M^+,7), 150 (47), 110 (100), 85 (29), 57 (26), 43 (29)
Mitchellene G	1695	22.98	m/z(rel.int.) 220 (M^+,43), 162 (100)
Eremophilone	1740	23.94	m/z(rel.int.): 218 (M^+, 64), 203 (29), 176 (100), 161 (58), 147 (40), 133 (74), 119 (58), 107 (83), 91 (79), 79 (75) 41 (40)
9-Hydroxydendrolasin	1741	23.97	m/z(rel.int.): 216 (M^+, 5), 150 (11), 135 (37), 107 (48), 93 (81), 81 (100), 65(22), 53(84), 41 (79)
Dehydrongaione	1742	24.2	m/z(rel.int.): 248 (M^+, 5), 151 (55), 83 (100), 55 (14), 43 (23)
10-Hydroxydihydro-α-humulene acetate	1755	24.04	m/z(rel.int.): 204 (M^+, 46), 189 (37), 161 (41), 147 (30), 121 (61), 109 (44), 93 (100), 80 (33), 67 (33), 43 (73)
Santalcamphor	1760	24.37	m/z(rel.int.): 236 (M^+, 29), 207 (100), 123 (40), 109 (52), 95 (35), 69 (57), 55 (31), 41 (31)
Myoporone	1837	25.97	m/z(rel.int.): 250 (M^+, 5), 150 (32), 110 (53), 95 (100), 85 (20), 57 (27), 41 (11)
9-Hydroxy-7(11), 9-eremophiladien-8-one	1849	26.21	m/z(rel.int.): 234 (M^+, 62), 219 (50), 201 (26), 191 (54), 177 (43), 163 (100), 153 (96), 137 (42), 91 (40), 41(33)
Dehydromyoporone	1901	27.26	m/z(rel.int.): 248 (M^+, 1), 95 (40), 83 (100), 55 (16), 39 (8)
Freelingnite	1987	30.93	m/z(rel.int.): 246 (M^+, 5), 149 (10), 97 (38), 81 (100), 53 (13), 41 (11)
Mitchellene B	2083	32.73	m/z(rel.int.): 232 (M^+, 47), 217 (11), 188 (100), 173 (31), 131 (62), 123 (94), 105 (57), 91 (61), 77 (42), 55 (26) 41 (21)
Oppositifolic acid	2149	34.07	m/z(rel.int.): 260 (M^+, 6), 200 (20), 185 (30), 157 (29), 145 (41), 131 (49), 105 (51), 91 (79), 79 (47), 43 (100)

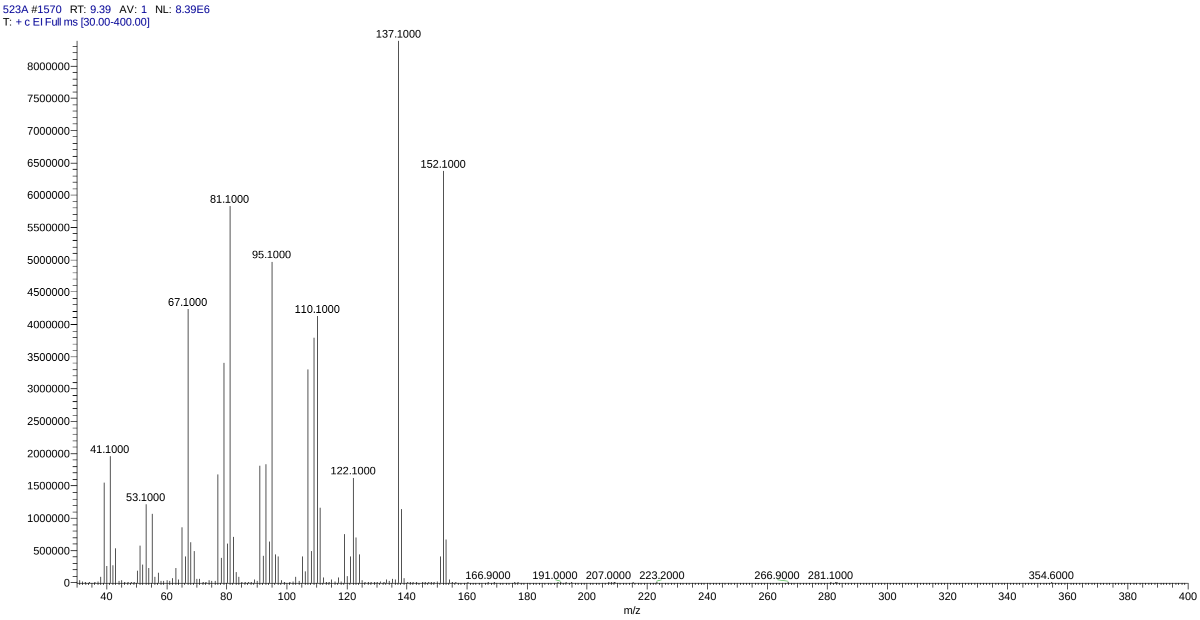

Figure A1-1 - Electron impact mass spectrum of ***Myodesert-1-ene****; Arithmetic index = 1141, DB5 column.*

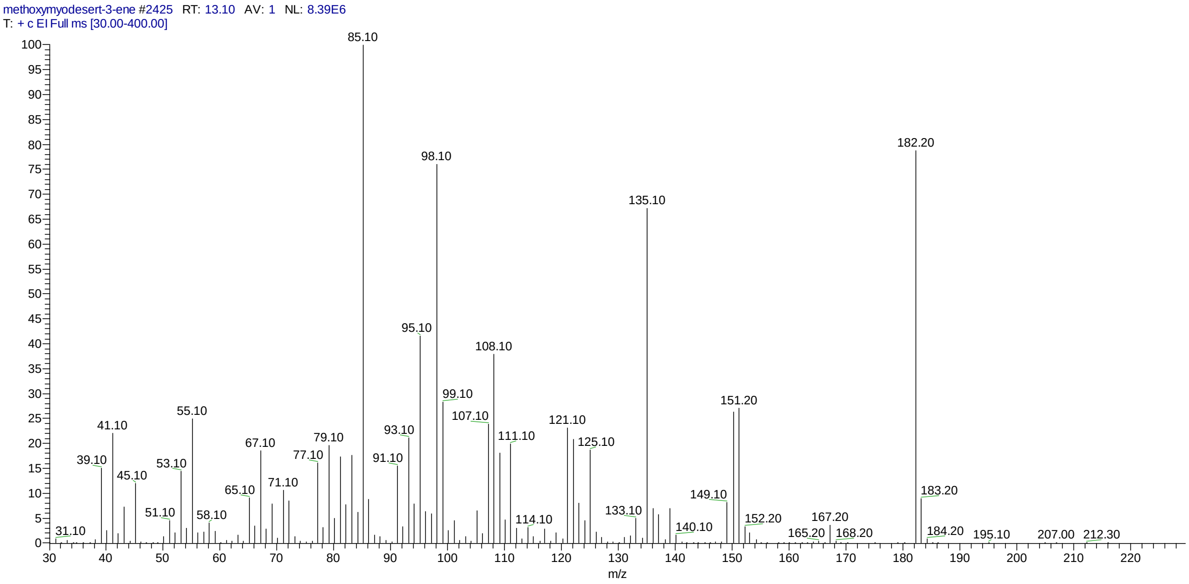

Figure A1-2 - Electron impact mass spectrum of ***Methoxymyodesert-3-ene****; Arithmetic index = 1284, DB5 column.*

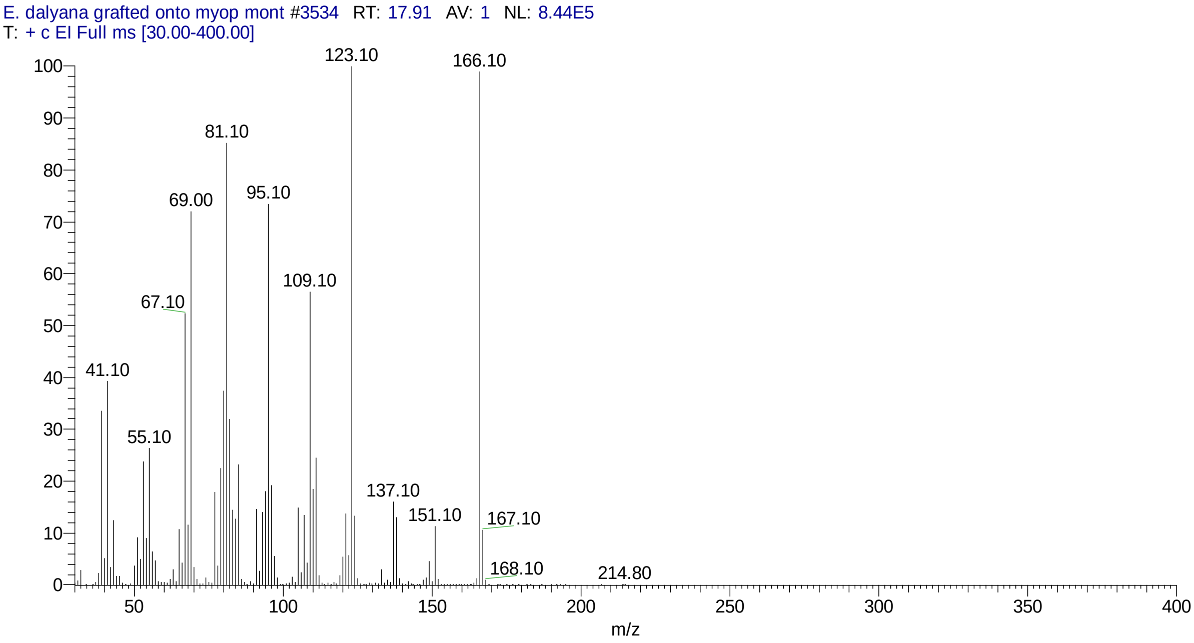

Figure A1-3 - Electron impact mass spectrum of ***Cis,Cis-Nepetalactone;*** *Arithmetic index = 1394, DB5 column.*

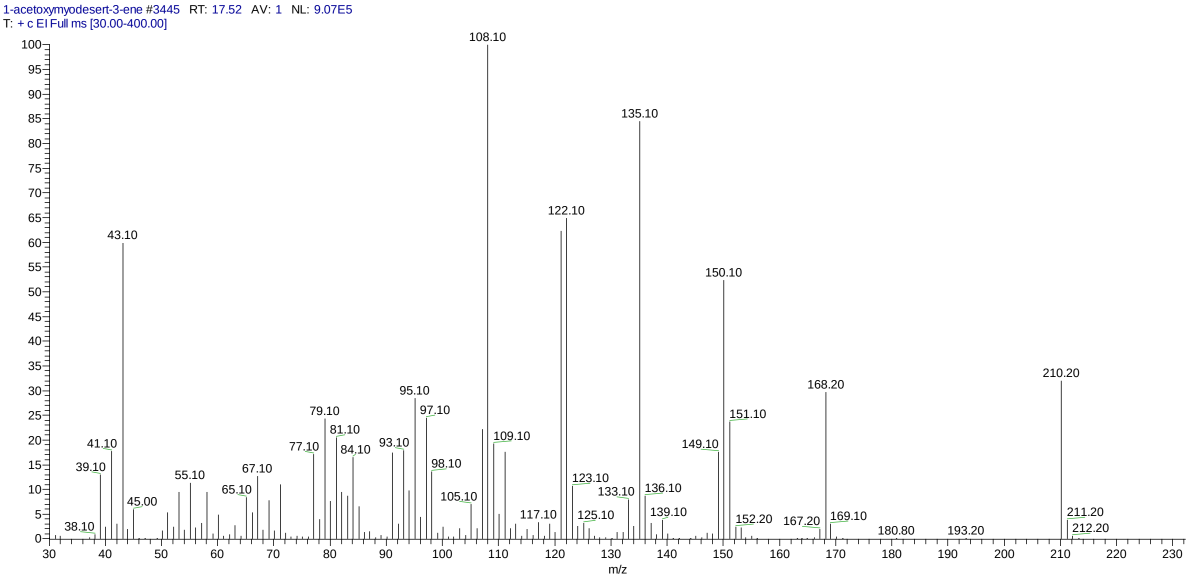

Figure A1-4 – Electron impact mass spectrum of ***1-Acetoxymyodesert-3-ene****; Arithmetic index = 1459, DB5 column.*

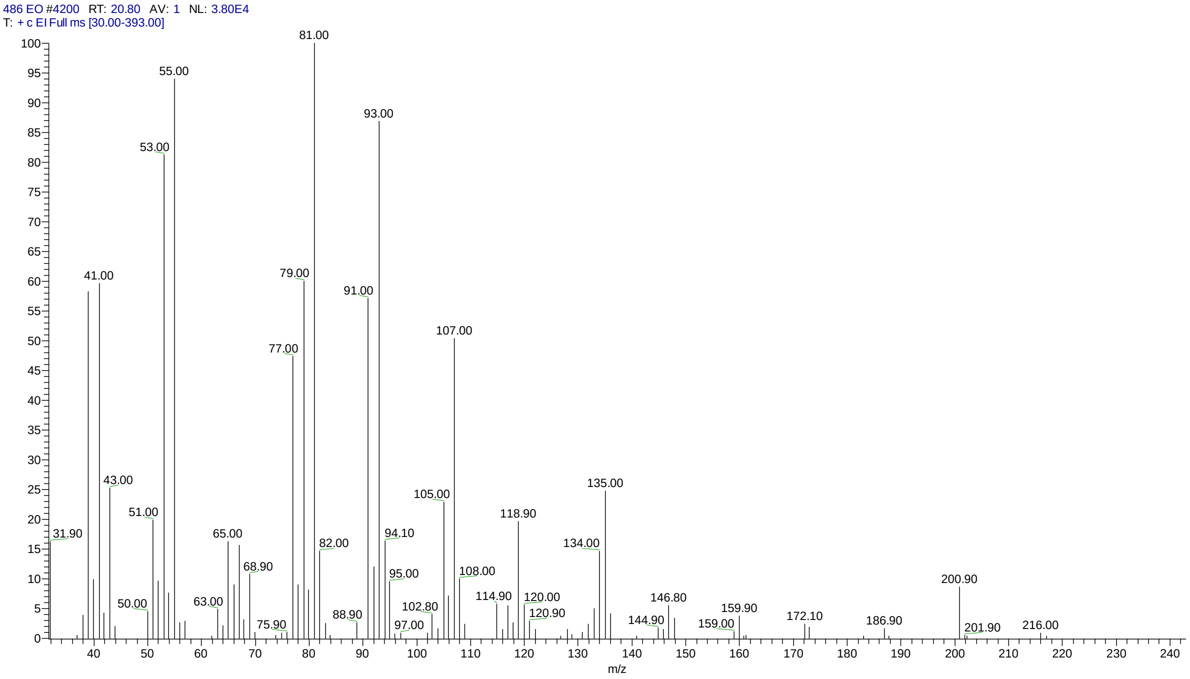

*Figure A1-5 - Electron impact mass spectrum of **Z-11,12-Dehydroisodendrolasin**; Arithmetic index = 1597, DB5 column.*

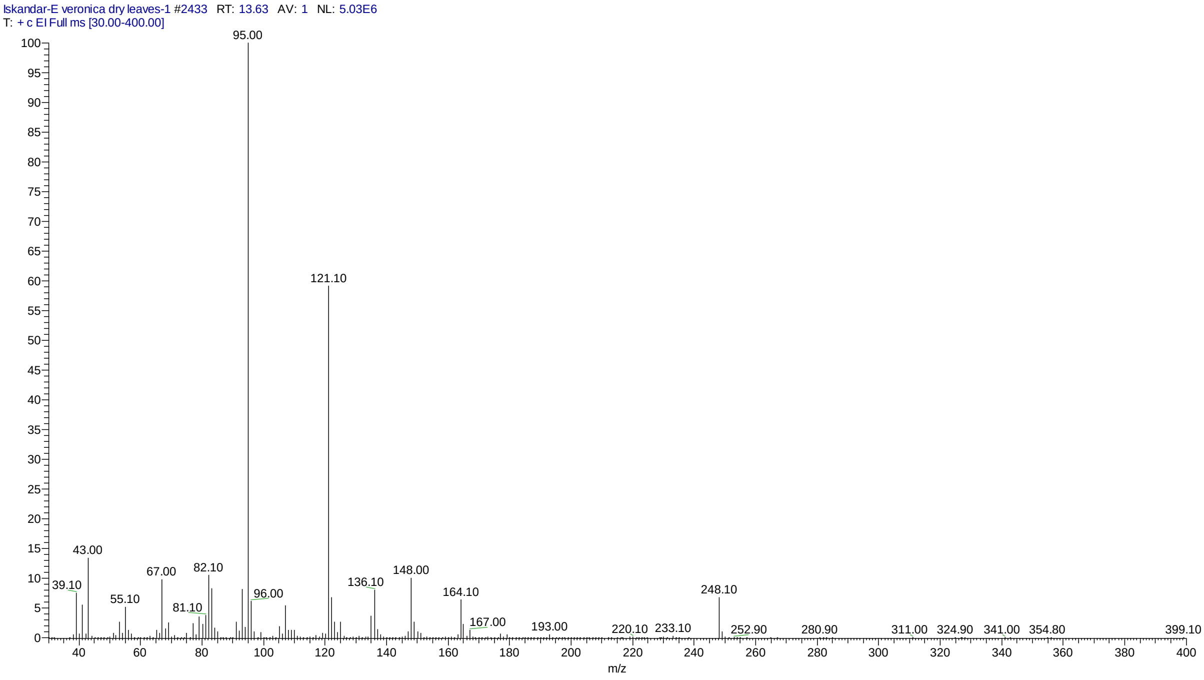

*Figure A1-6 - Electron impact mass spectrum of **Eremoacetal;** Arithmetic index = 1630, DB5 column.*

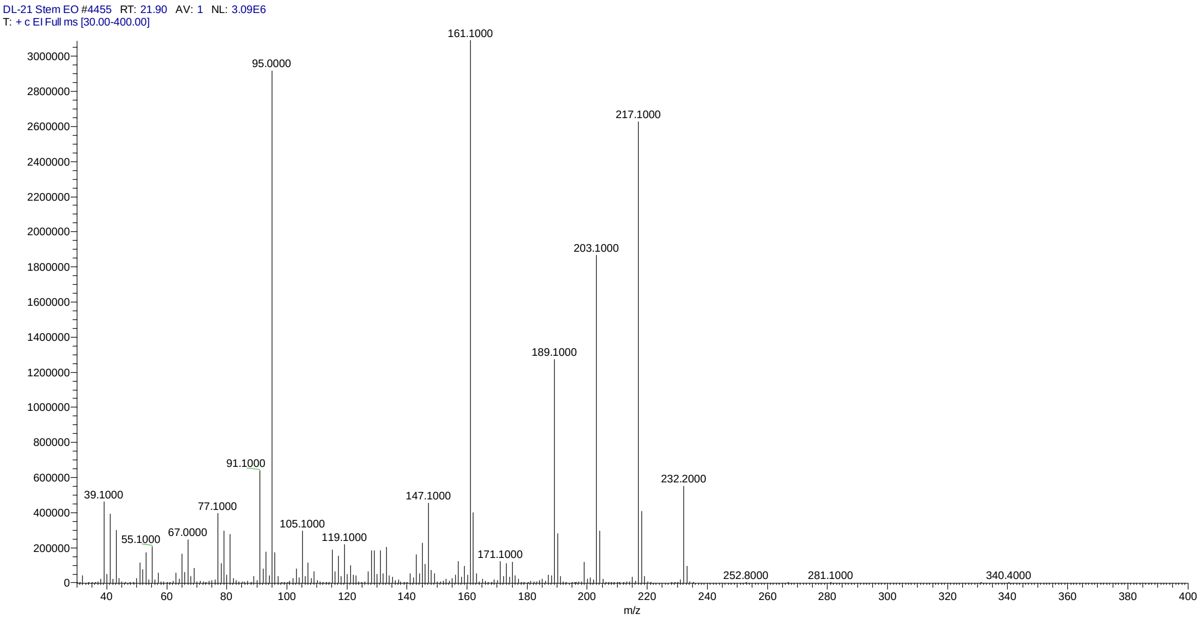

*Figure A1-7 - Electron impact mass spectrum of **Myomontanone**; Arithmetic index = 1646, DB5 column.*

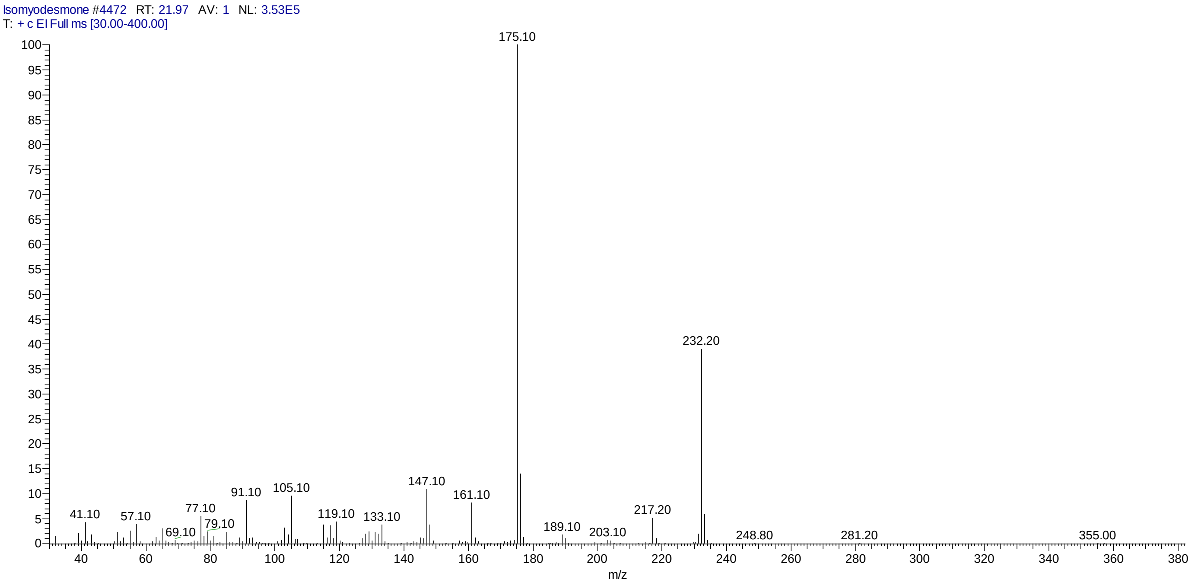

*Figure A1-8 - Electron impact mass spectrum of **Isomyodesmone**; Arithmetic index = 1649, DB5 column.*

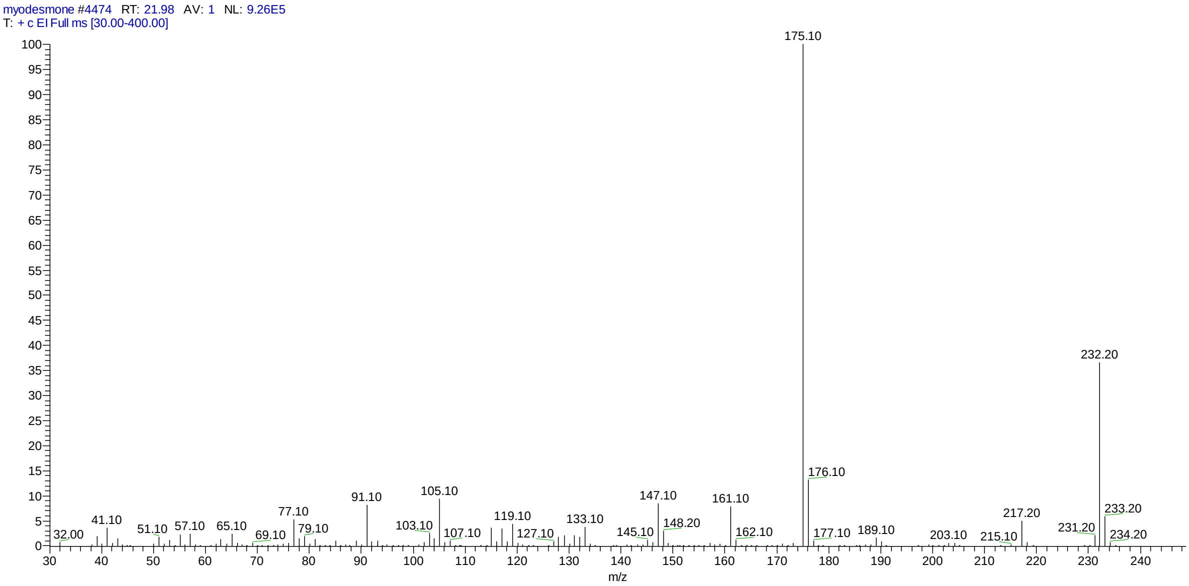

Figure A1-9 - Electron impact mass spectrum of ***Myodesmone****; Arithmetic index = 1650, DB5 column.*

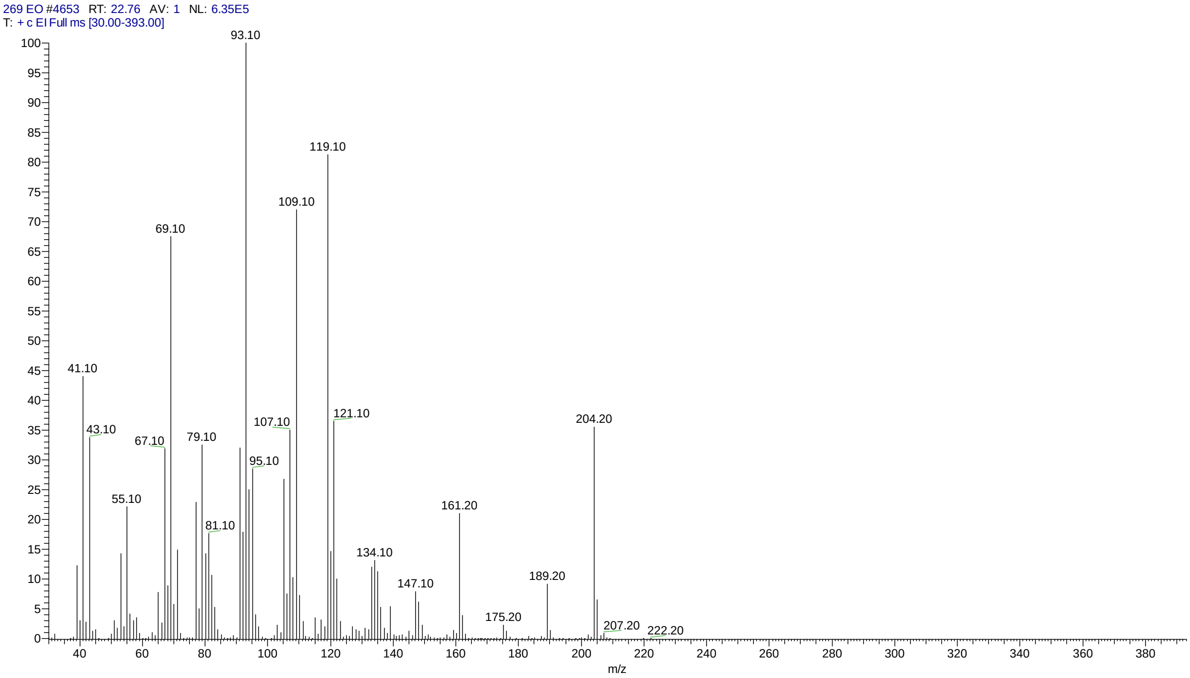

Figure A1-10 - Electron impact mass spectrum of ***Anymol****; Arithmetic index = 1668, DB5 column.*

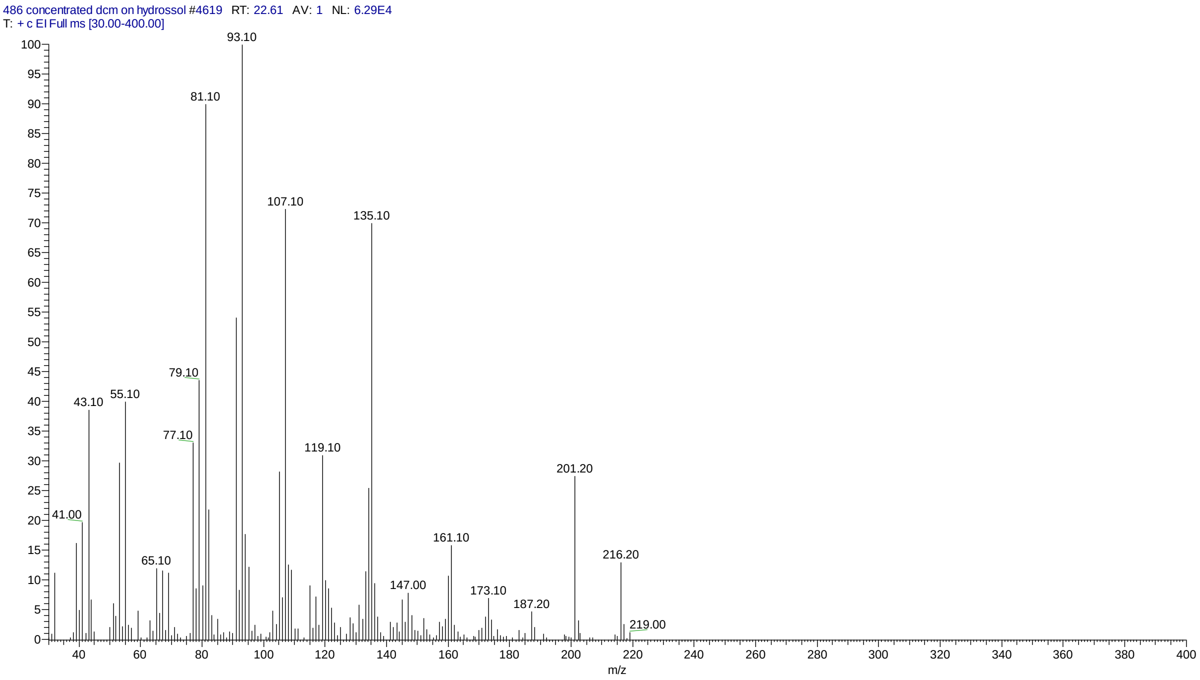

*Figure A1-11 - Electron impact mass spectrum of **Z-11-Hydroxydendrolasin**; Arithmetic index = 1677, DB5 column.*

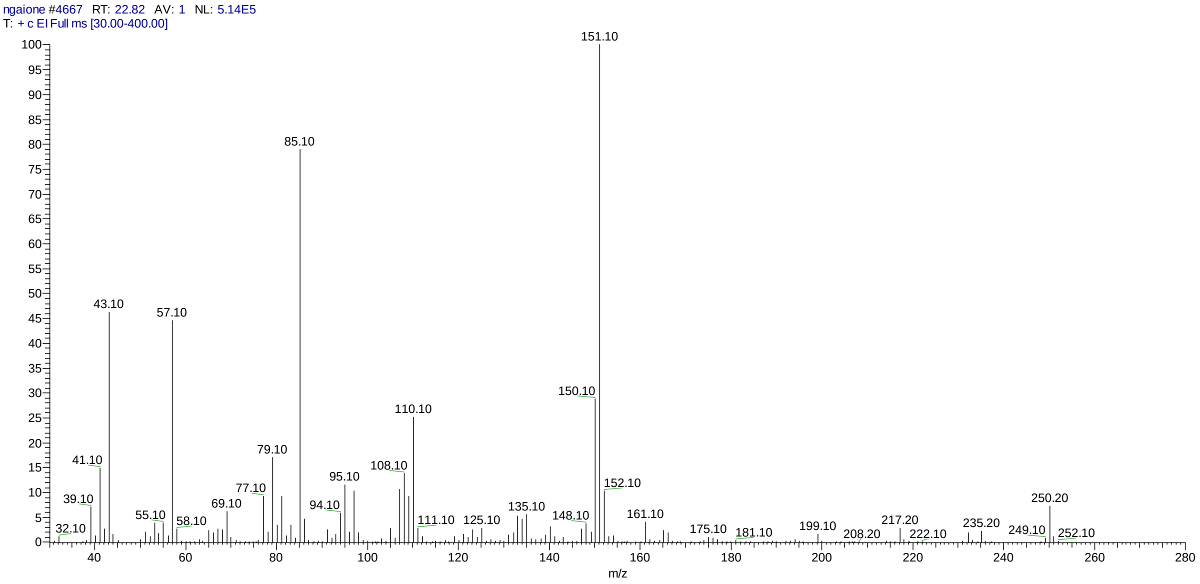

*Figure A1-12 - Electron impact mass spectrum of **Ngaione**; Arithmetic index = 1687, DB5 column.*

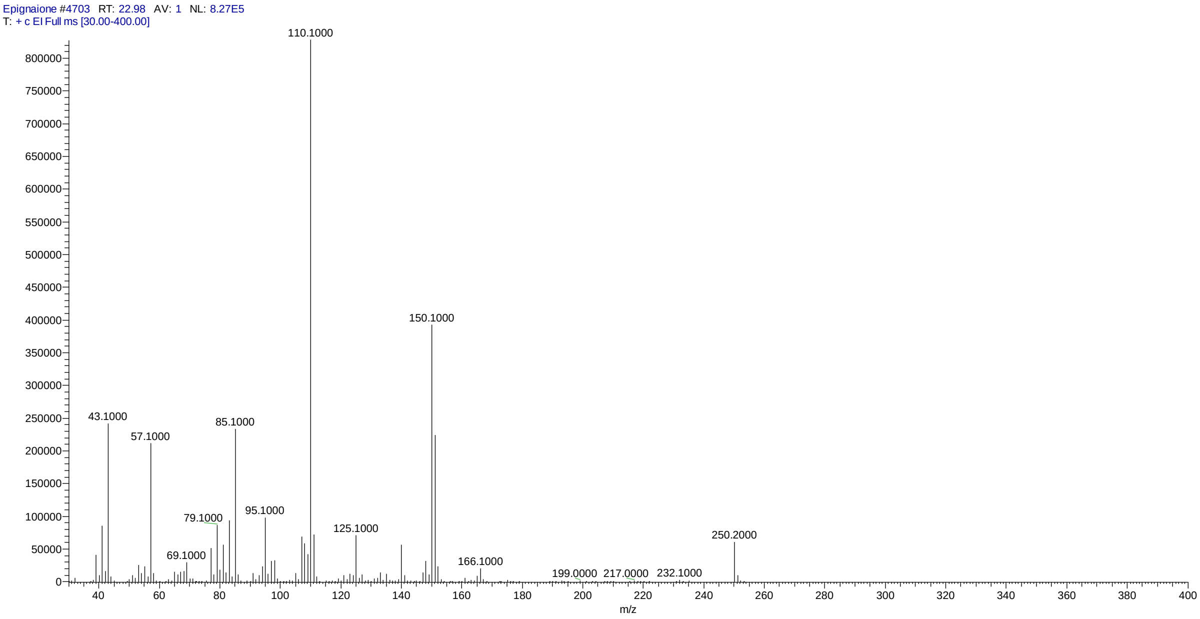

*Figure A1-13 - Electron impact mass spectrum of **Epingaione**; Arithmetic index = 1694, DB5 column.*

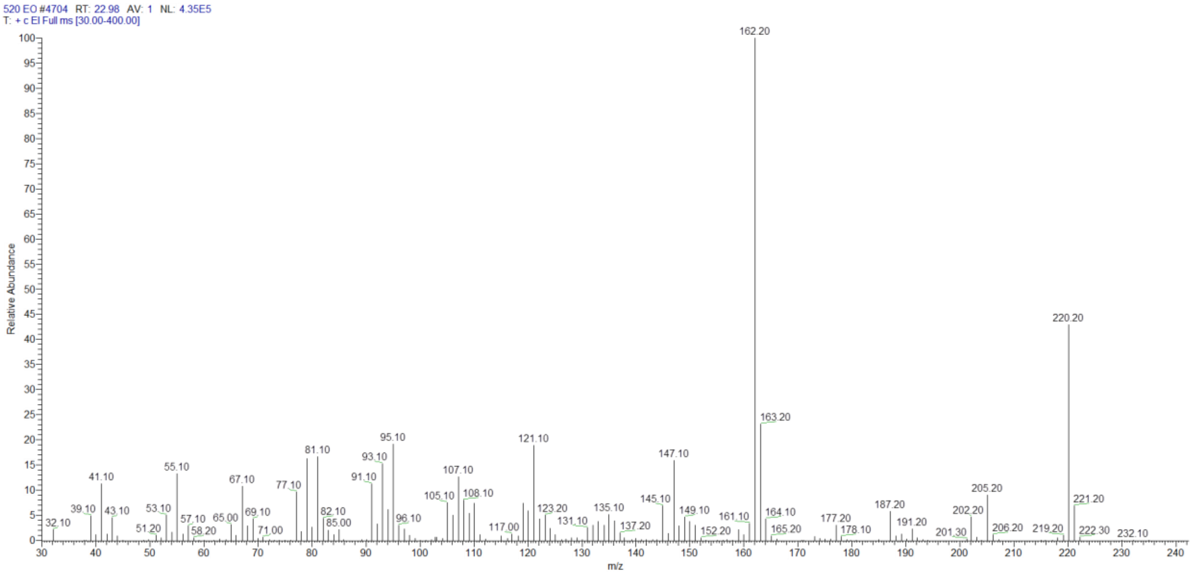

*Figure A1-14 - Electron impact mass spectrum of **Mitchellene G**; Arithmetic index = 1695, DB5 column.*

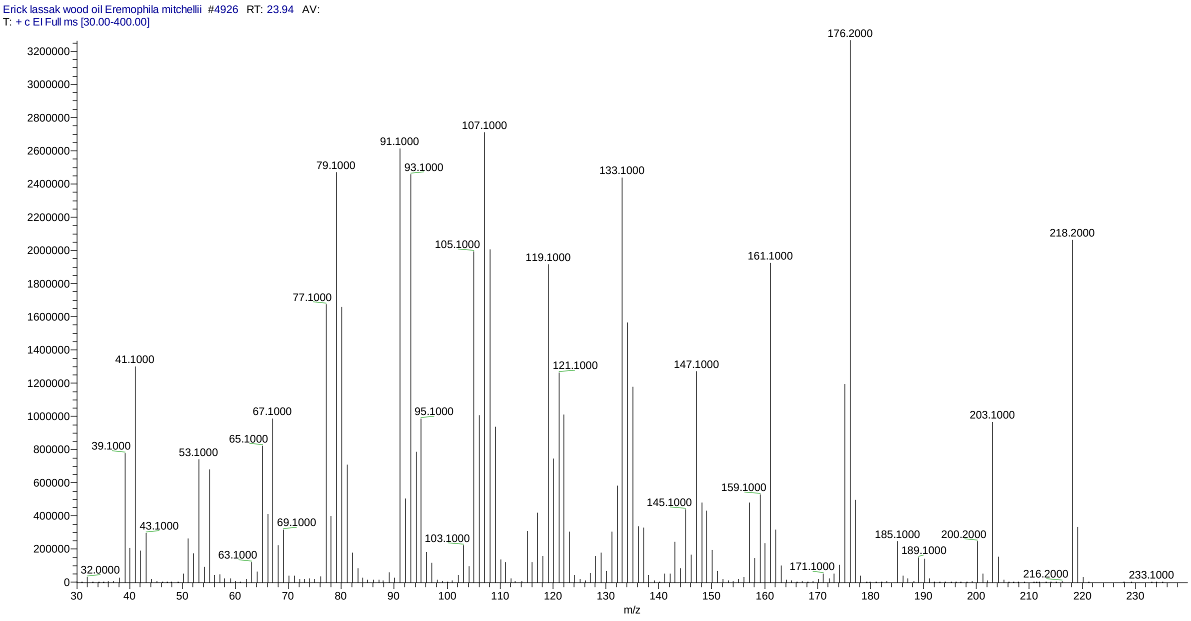

*Figure A1-15 - Electron impact mass spectrum of **Eremophilone;** Arithmetic index = 1740, DB5 column.*

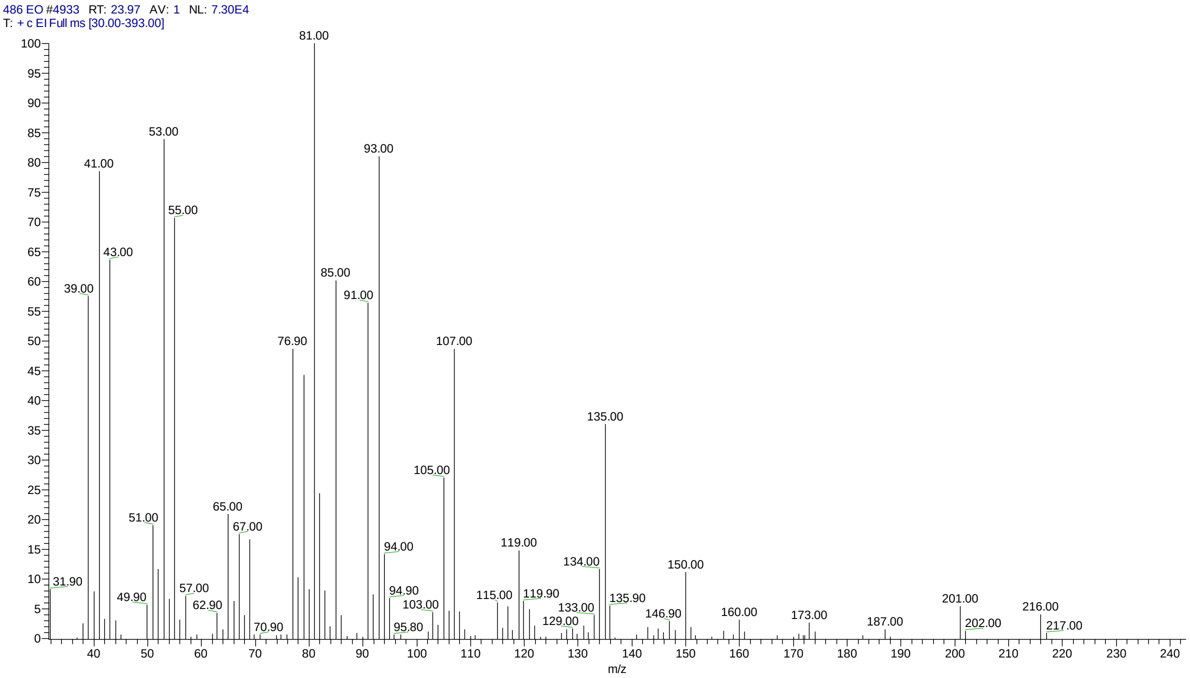

*Figure A1-16 - Electron impact mass spectrum of **9-Hydroxydendrolasin**; Arithmetic index = 1741, DB5 column.*

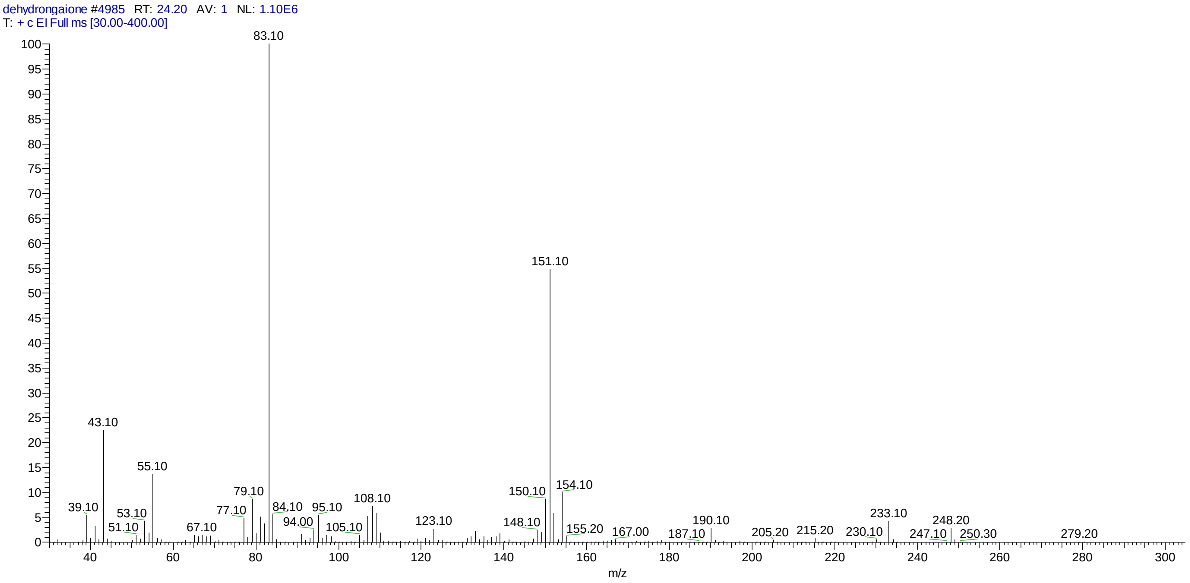

Figure A1-17 - Electron impact mass spectrum of ***Dehydrongaione****; Arithmetic index = 1742, DB5 column.*

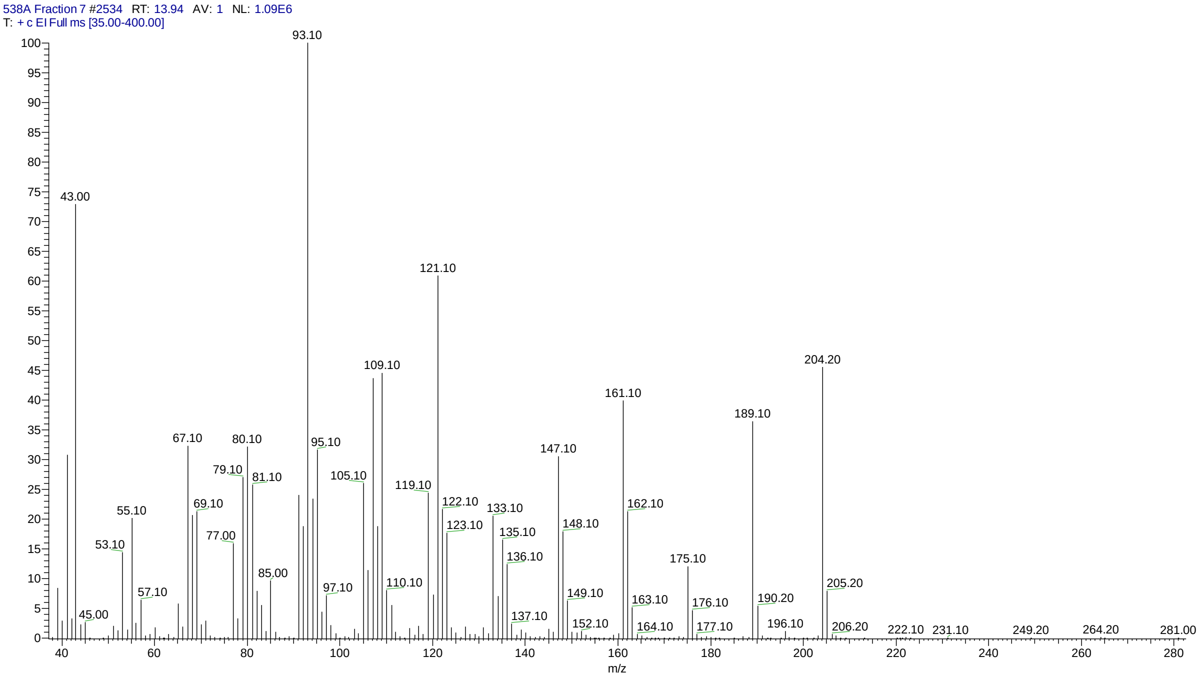

Figure A1-18 - Electron impact mass spectrum of ***10-Hydroxydihydro-α-humulene acetate;*** *Arithmetic index = 1755, DB5 column.*

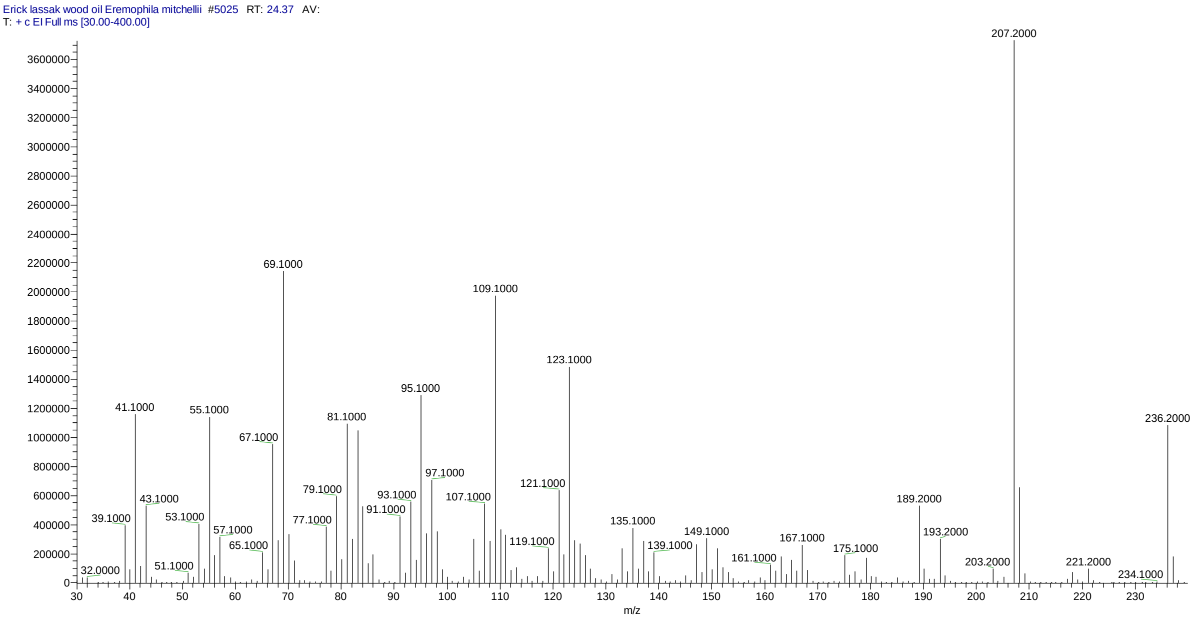

*Figure A1-19 - Electron impact mass spectrum of **Santalcamphor;** Arithmetic index = 1760, DB5 column.*

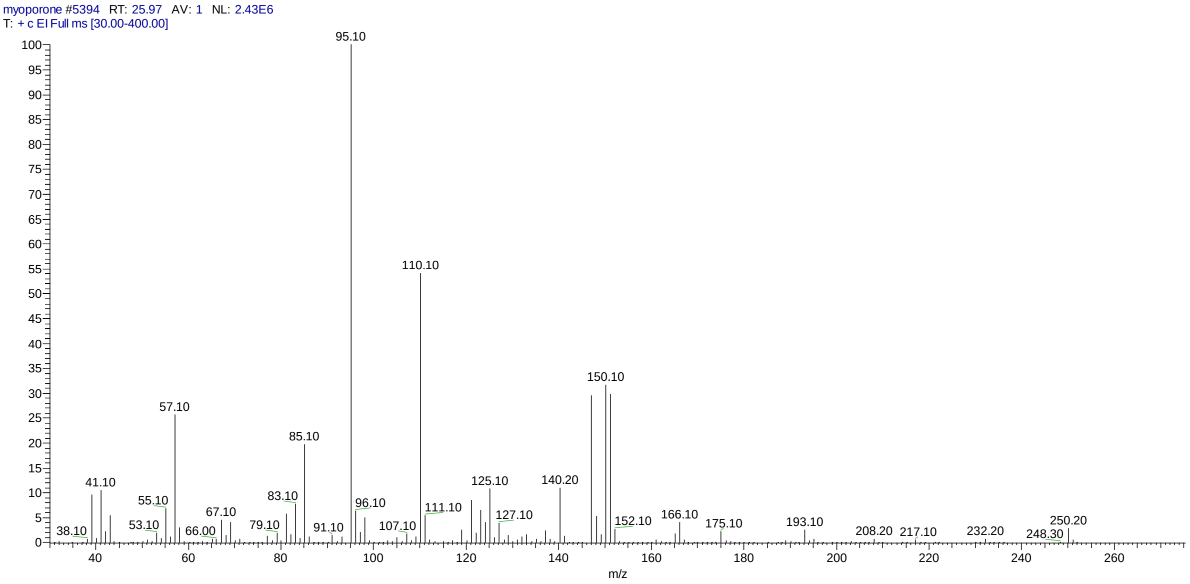

*Figure A1-20 - Electron impact mass spectrum of **Myoporone**; Arithmetic index = 1837, DB5 column.*

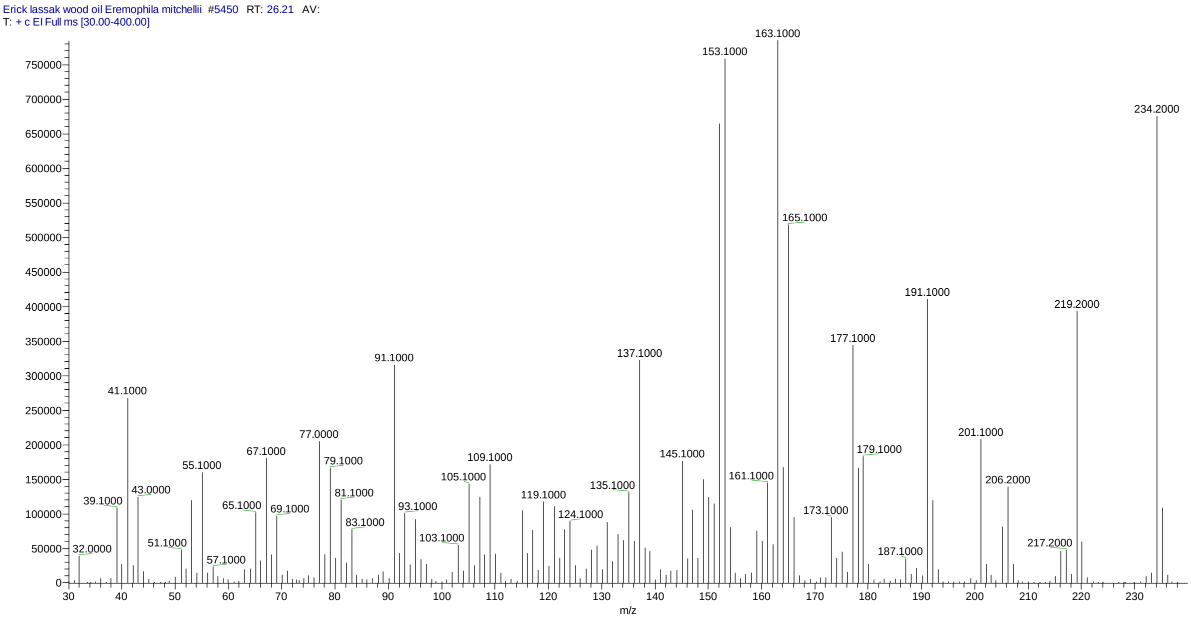

*Figure A1-21 - Electron impact mass spectrum of **9-hydroxy-7(11), 9-eremophiladien-8-one;** Arithmetic index = 1849, DB5 column.*

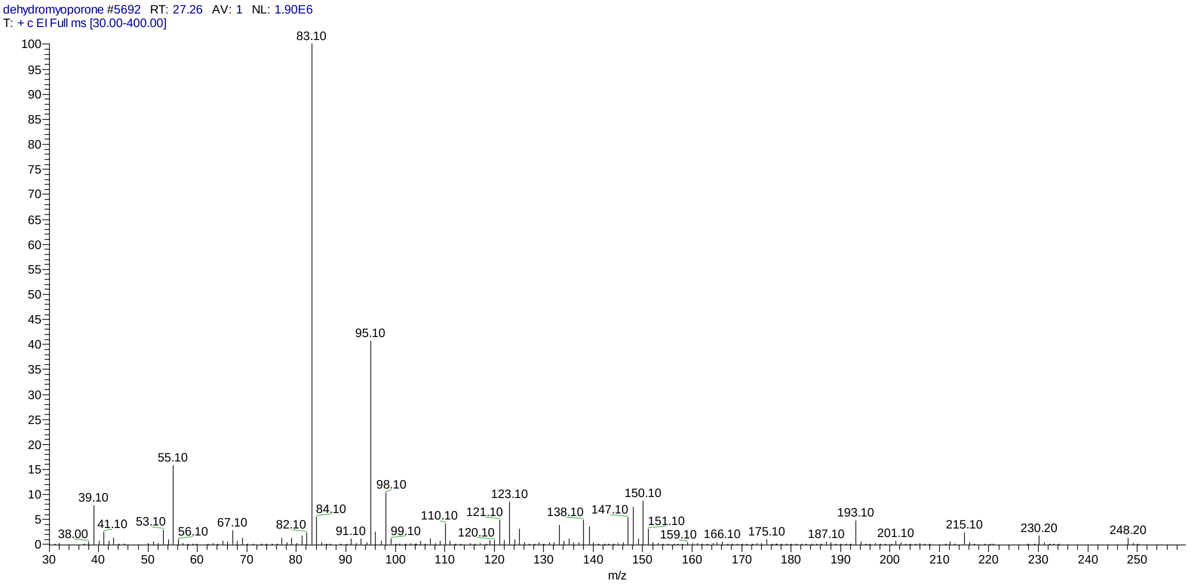

*Figure A1-22 - Electron impact mass spectrum of **Dehydromyoporone**; Arithmetic index = 1901, DB5 column.*

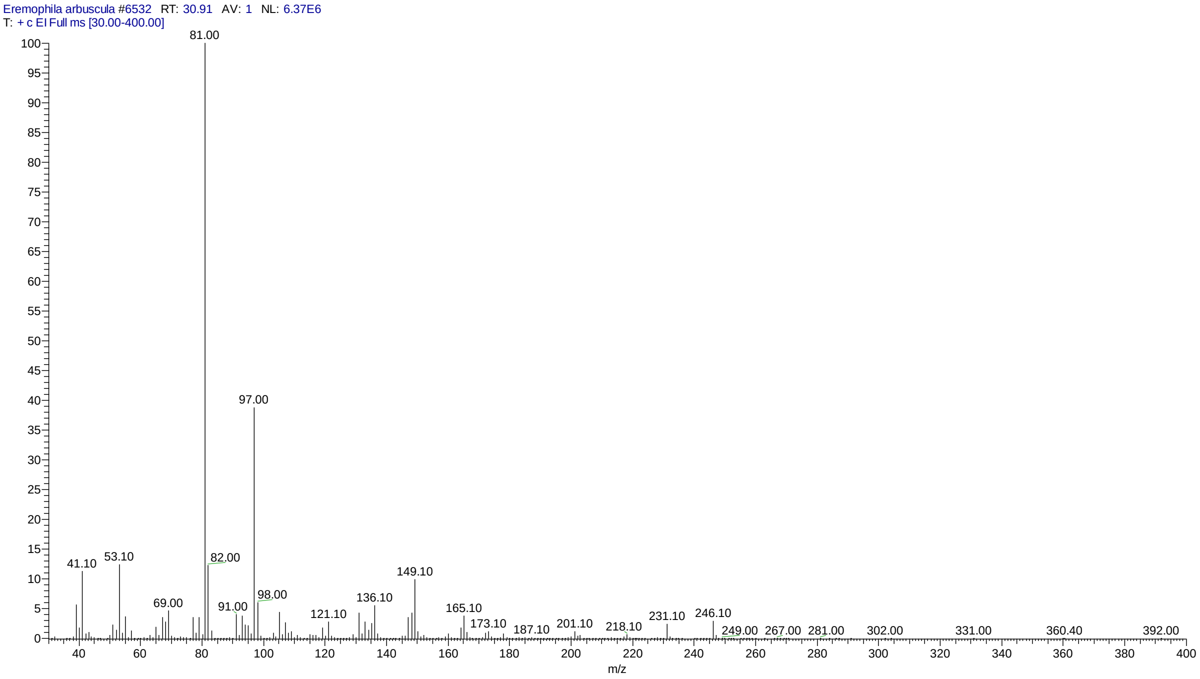

*Figure A1-23 - Electron impact mass spectrum of **Freelingnite**; Arithmetic index = 1987, DB5 column.*

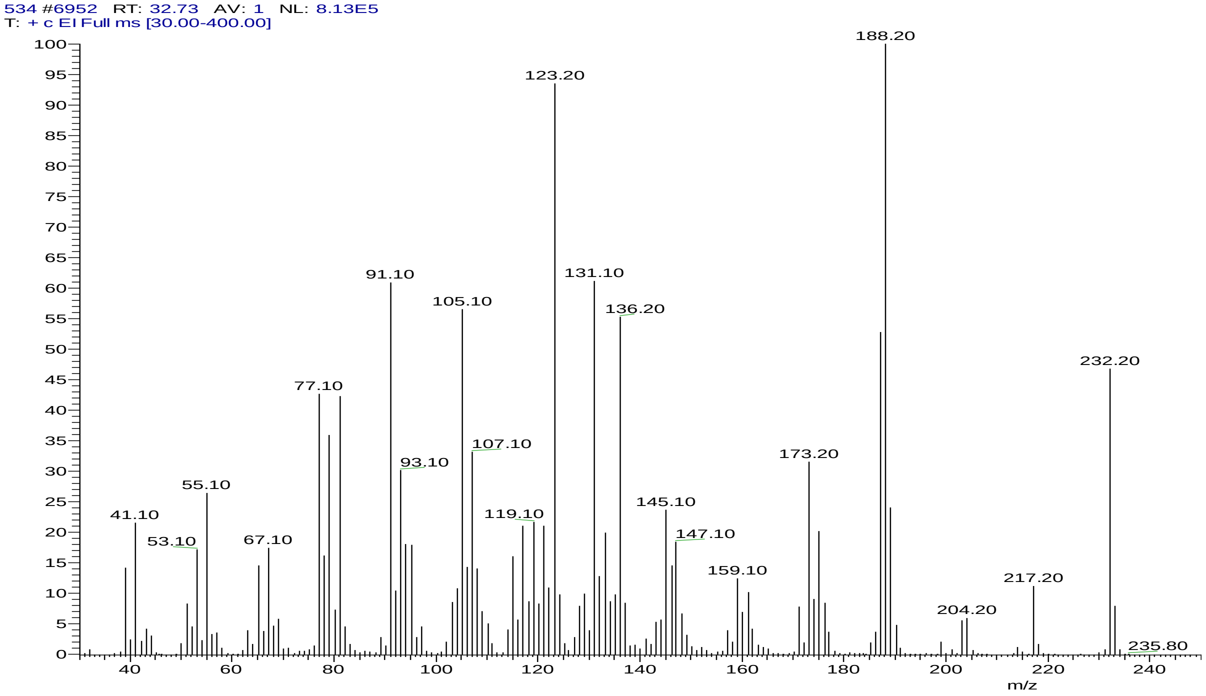

*Figure A1-24 - Electron impact mass spectrum of **Mitchellene B;** Arithmetic index = 2083, DB5 column.*

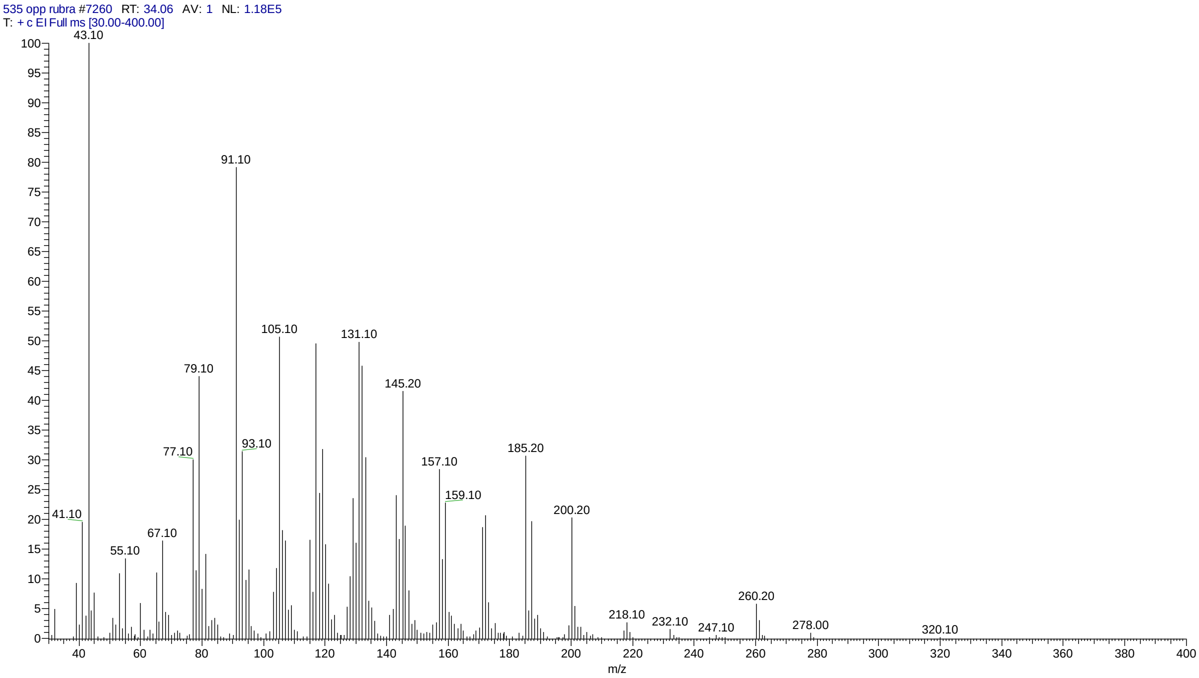

*Figure A1-25 - Electron impact mass spectrum of **Oppositifolic acid**; Arithmetic index = 2149, DB5 column.*

Supplementary Files – A2; full NMR shift values (tables only)

Table A2-2 – 13C NMR spectra (ppm) for rare compounds from the genus *Eremophila*

Name	AI	RT	13C NMR chemical shifts (ppm) for isolated compounds
Methoxymyodesert-3-ene	1284	13.1	134.3, 99.3, 113.8, 55.5, 43.9, 29.5, 32.9, 35.6, 38.0, 16.3, 16.5
1-Acetoxymyodesert-3-ene	1459	17.52	169.8, 132.7, 114.3, 89.7, 42.7, 38.9, 36.6, 32.3, 30.7, 21.6, 17.2, 15.1
Z-11,12-Dehydroisodendrolasin	1597	20.8	142.7, 142.3, 139.0, 134.8, 128.9, 125.0, 114.5, 111.2, 43.1, 28.7, 25.1, 18.9, 16.4
Myomontanone	1646	21.9	189.4, 153.0, 147.5, 143.7, 136.4, 128.6, 109.6, 45.6, 43.7, 39.4, 31.6, 21.2, 27.4, 22.8, 22.6
Myodesmone	1650	21.98	204.9, 143.0, 142.9, 142.5, 136.8, 121.4, 110.3, 51.2, 42.9, 36.3, 31.3, 24.9, 23.0, 22.8, 20.2
Anymol	1668	22.76	134.0, 131.8, 124.7, 120.9, 74.5, 43.5, 39.5, 31.2, 26.2, 25.8, 24.1, 24.1, 23.5, 22.4, 17.8
Z-11-Hydroxyisodendrolasin	1677	22.6	142.7, 139.5, 139.0, 134.6, 125.3, 125.0, 124.8, 111.2, 70.8, 29.9, 28.6, 25.1, 16.2, 42.4
Ngaione	1687	22.82	209.5, 143.4, 139.3, 127.3, 109.0, 81.9, 72.7, 54.4, 53.8, 37.2, 33.2, 26.8, 24.5, 22.7
Epingaione	1694	22.98	209.7, 143.5, 139.4, 127.6, 108.8, 81.9, 73.7, 53.8, 53.8, 37.4, 33.6, 28.1, 24.6, 22.7, 22.7
Mitchellene G	1695	22.98	133.3, 126.0, 75.4, 48.4, 47.3, 45.1, 40.4, 37.9, 35.3, 30.8, 29.0, 24.0, 20.9, 19.3, 11.3
Eremophilone	1740	23.94	203.8, 147.7, 144.4, 135.3, 110.1, 43.3, 41.5, 39.2, 39.0, 36.0, 26.6, 25.7, 24.9, 20.7, 16.1
9-Hydroxydendrolasin	1741	23.97	142.9, 139.0, 135.1, 132.6, 128.0, 127.6, 124.8, 111.1, 65.9, 48.3, 28.6, 25.9, 24.9, 18.3, 16.3
Dehydrongaione	1742	24.2	199.2, 154.9, 143.2, 139.2, 127.3, 125.5, 108.9, 82.2, 72.7, 55.5, 36.9, 33.3, 27.7, 27.0, 20.7
10-Hydroxydihydro-α-humulene acetate	1755	24.04	170.9, 134.3, 131.9, 127.7, 124.5, 72.6, 46.3, 39.5, 42.8, 39.0, 33.7, 31.0, 27.1, 25.3, 21.8
Santalcamphor	1760	24.37	211.7, 145.1, 112.2, 76.6, 54.1, 48.1, 41.2, 40.0, 33.8, 22.3, 21.4, 20.9, 19.5, 15.4
Myoporone	1837	25.97	210.5, 195.1, 147.2, 144.3, 127.8, 108.8, 52.6, 50.7, 38.3, 31.2, 28.9, 24.6, 22.7, 22.7, 19.9
9-Hydroxy-7(11), 9-eremophiladien-8-one	1849	26.21	185.6, 146.6, 142.6, 137.3, 125.8, 43.0, 40.7, 39.8, 30.7, 25.7, 23.1, 22.9,16.3,15.7
Dehydromyoporone	1901	27.26	200.7, 195.2, 155.4, 147.2, 144.3, 127.8, 124.2, 108.8, 51.6, 38.4, 31.4, 29.6, 27.8, 20.9, 20.0
Freelingnite	1987	30.93	174.3, 148.8, 142.8, 139.0, 130.2, 130.0, 128.5, 124.7, 111.1, 80.0, 43.6, 28.5, 24.8, 16.8, 10.7
Mitchellene B	2083	32.73	171.9, 137.2, 128.4, 83.8, 46.2, 44.6, 42.9, 42.1, 35.3, 33.0, 29.5, 28.7, 23.3, 18.9, 11.6
Oppositifolic acid	2149	34.07	171.9, 170.9, 141.7, 141.5, 136.7, 131.0, 129.6, 122.1, 58.5, 33.5, 32.0, 29.3, 29.3, 29.2, 22.8
4-Hydroxyngaione i.e., ngaiol			207.5, 143.4, 139.3, 127.1, 109.1, 72.8, 81.9, 55.4, 58.7, 41.5, 37.2, 33.0, 32.6, 32.6, 26.9
Mitchellene F			136.6, 128.3, 76.7, 68.6, 48.6, 45.8, 44.7, 35.3, 37.9, 40.4, 29.1, 30.4, 19.3, 20.8, 11.3,
t-Muurolol			133.6, 125.0, 72.5, 46.2, 44.1, 34.8, 34.6, 31.4, 29.4, 26.8, 23.7, 21.7, 21.1, 19.5, 15.5

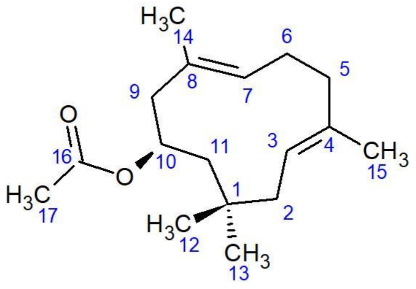

10-hydroxydihydro-α-humulene acetate

*Table A2-3 - NMR data for **10-hydroxydihydro-α-humulene acetate***

-	13C δ	Appearance	Hydrogens	Multiplicity	1H δ	J (Hz)	HMBC
1	33.67	Thin	-	-	-	-	2, 11, 12, 13
11	42.79	Broad	2H	d	1.8	15.2	3, 12, 13
-	-	-	-	ddd	1.35	0.6, 8.03, 15.2	-
10	72.63	Broad	1H	dt	4.48	0.6, 8.98	4, 12, 14, 16
9	46.35	Broad	2H	dd	2.09	10.6, 12.1	-
-	-	-	-	dd	1.96	10.6, 12.2	-
8	131.89	Thin	-	-	-	-	14
7	127.67	Thin	1H	dd	4.93	5.37, 9.61	14
6	25.3	Thin	2H	ddd	2.3	4.7, 10.5, 13.2	-
-	-	-	-	n.d.	2.09	-	-
5	39.52	Thin	2H	ddd	2.17	0.95, 6.5, 11.6	11
-	-	-	-	ddd	2.04	5.6, 6.5, 11.6	-
4	134.31	Thin	-	-	-	-	15
3	124.51	Thin	1H	dd	4.86	3.6, 8.1	15
2	39.05	Broad	2H	n.d.	2.03	-	2, 8, 11, 15
-	-	-	-	brd	1.78	14.9	-
12	30.97	Broad	3H	S	0.96	-	2, 11, 13
13	27.12	Broad	3H	S	0.86	-	2, 11, 12
14	18.03	Thin/Broad	3H	S	1.69	-	3
15	16.07	Thin	3H	S	1.48	-	10
17	21.75	Thin	3H	S	2.01	-	3, 17
16	170.9	Thin	-	-	-	-	16

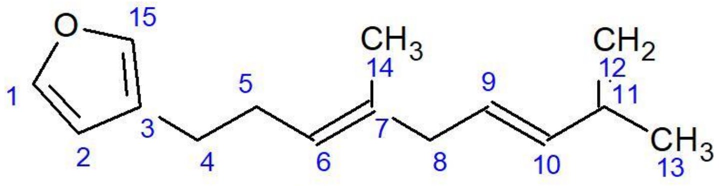

11,12-dehydroisodendrolasin

*Table A2-4 - NMR data for **11,12-dehydroisodendrolasin***

-	13C (ppm)	Proton	Multi	J (Hz)	1H (ppm)	COSY	HMBC
1	142.72	1H	t	1.6	7.34	2	-
2	111.24	1H	m	-	6.28	1	-
3	125.06	-	-	-	-	-	-
4	25.13	2H	dd	7.2, 7.9	2.46	5	-
5	28.72	2H	q	7.5	2.26	4, 6	-
6	124.91	1H	tq	1.3, 7.1	5.21	5	7, 8
7	134.75	-	-	-	-	-	14
8	43.08	2H	d	7.0	2.76	9	6, 7, 9, 10
9	128.90	1H	dt	7.0, 15.6	5.62	8, 10	-
10	134.15	1H	brd	15.6	6.13	9	8, 13
11	142.26	-	-	-	-	-	13
12	114.76	2H	s	-	4.88	-	13
13	18.87	3H	t	1.0	1.84	-	10, 12
14	16.36	3H	s	-	1.53	-	5, 6
15	139.00	1H	dt	1.0, 2.5	7.21	-	4

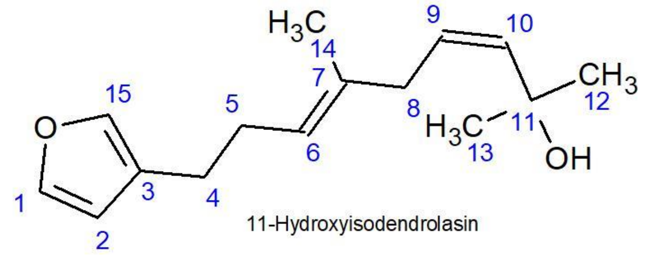

11-Hydroxyisodendrolasin

*Table A2-5 - NMR data for **11-Hydroxyisodendrolasin***

-	13C (ppm)	Proton	Multi	J (Hz)	1H (ppm)	COSY	HMBC
1	142.71	1H	t	1.7	7.34	2	2, 15
2	111.24	1H	dd	0.8, 1.7	6.37	1	4, 15
3	125.02	-	-	-	-	-	2
4	25.09	2H	t	7.6	2.46	5	5
5	28.64	2H	q	7.2	2.25	4, 6	4
6	124.83	1H	tq	1.3, 7.2	5.18	5	14
7	134.65	-	-	-	-	-	5, 8, 14
8	42.46	2H	d	5.6	2.67	9	6, 14
9	125.37	1H	t	5.6	5.59	-	12, 13
10	139.49	1H	s	-	5.6	-	8, 12, 13
11	70.83	-	-	-	-	-	12, 13,
12	29.98	3H	s	-	1.31	-	10, 13
13	29.98	3H	s	-	1.31	-	10, 12
14	16.24	3H	s	-	1.56	-	6, 8
15	139.02	1H	dt	0.8, 2.4	7.21	-	1, 2, 4

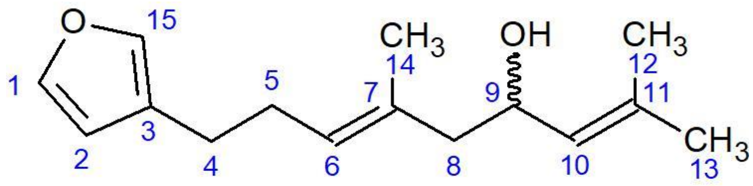

*Table A2-6- NMR data for **9-Hydroxydendrolasin***

-	13C	Proton	Multi	J (Hz)	1H
1	142.87	1H	t	1.1	7.34
2	111.07	1H	brS	-	6.27
3	124.8	-	-	-	-
4	24.95	2H	dd	7.4,	2.48
5	28.6	2H	dt	7.4	2.29
6	128.02	1H	t	6.9	5.27
7	132.55	-	-	-	-
8	48.28	2H	d	6.7	2.13
9	65.94	1H	dd	6.8, 8.1	4.40
10	127.59	1H	td	1.1, 8.4	5.14
11	135.07	-	-	-	-
12	25.88	3H	d	1.2	1.72
13	18.32	3H	d	1.3	1.68
14	16.34	3H	brS	-	1.63
15	139.02	1H	brS	-	7.21

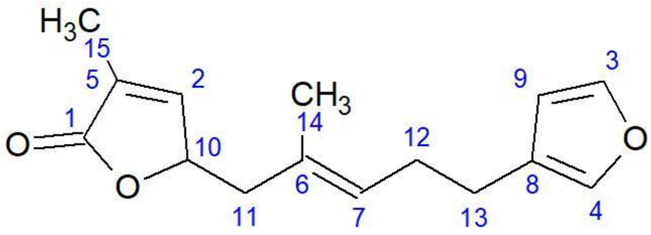

*Table A2-7 - NMR data for **freelingnite***

	13C	Hydro No.	1H ppm	Multi	J (Hz)	Key Cosy	Key HMBC
1	174.26	-	-	-	-	-	2, 15
2	148.81	1H	6.94	brt	1.5	10, 15 (long range)	15
3	142.78	1H	7.33	t	1.5	9	-
4	138.97	1H	7.20	brS	-	13	-
5	130.22	-	-	-	-	-	15
6	129.96	-	-	-	-	-	2, 11, 14
7	128.48	1H	5.26	dd	7.1, 7.2	12, 14 (long range)	-
8	124.71	-	-	-	-	-	3, 4, 9, 12, 13
9	111.08	1H	6.26	brS	-	3	-
10	80.04	1H	4.93	ddt	1.7, 7.0, 7.2	11, 15 (long range)	2, 15
11a	43.58	2H	2.38	dd	7.2, 13.8	10,	14, 15
11b	-	-	2.24	dd	7.0, 13.8	-	-
12	28.49	2H	2.27	dt	7.1, 7.3	7, 13	-
13	24.75	2H	2.46	dd	n.d., 7.3	4, 12	14
14	16.83	3H	1.65	d	0.9	-	-
15	10.73	3H	1.89	dd	1.5, 1.7	2, 10	-

*Table A2-8 - NMR data for **t-Muurolol***

-	13C δ	Multiplicity	1H δ	*J* (Hz)
1	133.96	-	-	-
2	131.85	-	-	-
3	124.72	tp	5.12	1.4, 7.2
4	120.91	dt	5.39	1.4, 3.5
5	74.47	-	-	-
6	43.46	ddq	1.55	2.3, 11.5, 12.6
7	39.48	2H, dt	1.5	1.8, 7.0
8	31.20	2H, dt	1.97	5.7, 17.3
9	26.22	n.d.	2.04	n.d.
-	-	tp	1.86	2.0, 13.2
10	25.84	3Hs	1.64	broad
11	24.13	ddt	1.79	2.3, 7.5, 12.3
-	-	dq	1.26	5.6, 12.3
12	24.08	3Hs	1.13	-
13	23.46	3Hd	1.68	1.4
14	22.42	2H, dd	2.04	8.7, 16.6?
15	17.80	3HS	1.62	-

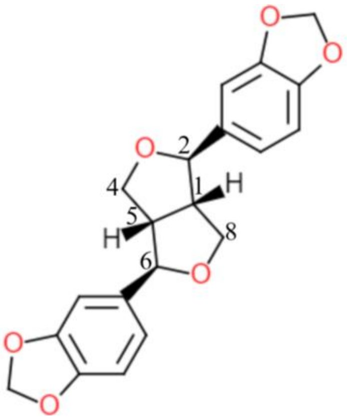

Sesamin.

*Table A2-9 -1H NMR spectra of **sesamin** in comparison with its commercial literature spectra.*

	^{1}H δ ppm	
Position	Experimental	Literature
1,5	3.05, *m*, 2H	3.05, *m*, 2H
2,6	4.72, *d*, *J* = 4.6 Hz, 2H	4.71, *d*, *J* = 4.5 Hz, 2H
4a,8a	3.87, *dd*, *J* = 3.9 Hz, 2H	3.87, *dd*, *J* = 4, 9 Hz, 2H
4b,8b	4.24, *m*, 2H	4.24, *dd*, *J* = 7, 9 Hz, 2H
OCH_2O	5.95, *s*, 4H	5.95, *s*, 4H
Aromatics	6.75–6.88, *m*, 6H	6.77–6.84, *m*, 6H

*Table A2-10 - 13C NMR spectra of **sesamin** in comparison with its commercial literature spectra.*

	^{13}C δ ppm	
Position	Experimental	Literature
1,5	53.4	54.3
2,6	85.8	85.8
4a,8a	71.7	71.7
4b,8b	-	-
OCH_2O	101.1	101.2
Aromatics	-	-
1’	135.1	134.9
2’	106.5	106.6
3’	147.2	147.3
4’	148.0	148.1
5’	108.2	108.3
6’	119.4	119.5

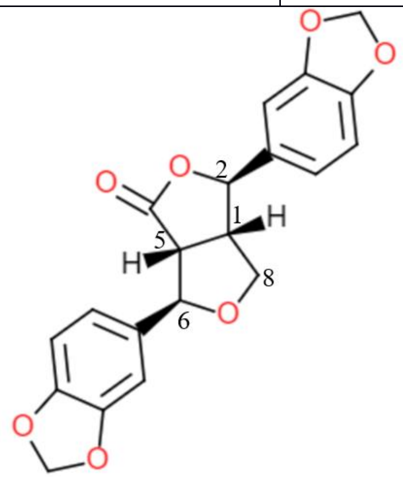

Aptosimon.

*Table A2-11 -1H NMR spectra of **aptosimon** in comparison with its literature spectra.*

	^{1}H δ ppm	
Position	Experimental	Literature
1	3.05, *m,* 1H	3.2, *m,* 1H
2	5.29, *d, J*=3.9 Hz, 1H	5.28, *d, J*=3.9 Hz, 1H
4	-	-
5	3.42, *dd, J*=3.7, 9.2 Hz, 1H	3.42, *dd,* J=9.0, 3.4 Hz, 1H
6	5.3, *d, J*=3.9 Hz, 1H	5.30, *d, J*=3.4 Hz, 1H
8a	4.33, *dd, J*=6.9, 9.6 Hz, 1H	4.32, *dd, J*= 9.6, 6.8 Hz, 1H
8b	4.00, *dd, J*=4.6, 9.5 Hz, 1H	4.00, *dd,* J=9.6, 4.9 Hz, 1H
OCH_2O	5.96, *s,* 2H	5.95, *s,* 2H
OCH_2O	5.98, *s,* 2H	5.97, *s,* 2H
Aromatics	6.75–6.88, *m,* 6H	6.75–6.86, *m,* 6H

*Table A2-12 - 13C NMR spectra of **aptosimon** in comparison with its literature spectra.*

	^{13}C δ ppm	
Position	Experimental	Literature
1	50.0	49.9
2	83.4	83.3
4	176.7	176.6
5	53.2	53.2
6	84.4	84.3
8a	72.7	72.6
8b	-	-
OCH_2O	101.2	101.1
OCH_2O	101.5	101.4
Aromatics	-	-
1’	133.0	133.0
1”	134.3	134.3
2’	105.7	105.7
2”	106.0	105.9
3’	147.3	147.2
3”	147.3	147.2
4’	148.0	148.0
4”	148.4	148.3
5’	108.4	108.3
5”	108.6	108.5
6’	118.8	118.8
6”	119.0	119.0

Supplementary A3 - Structures

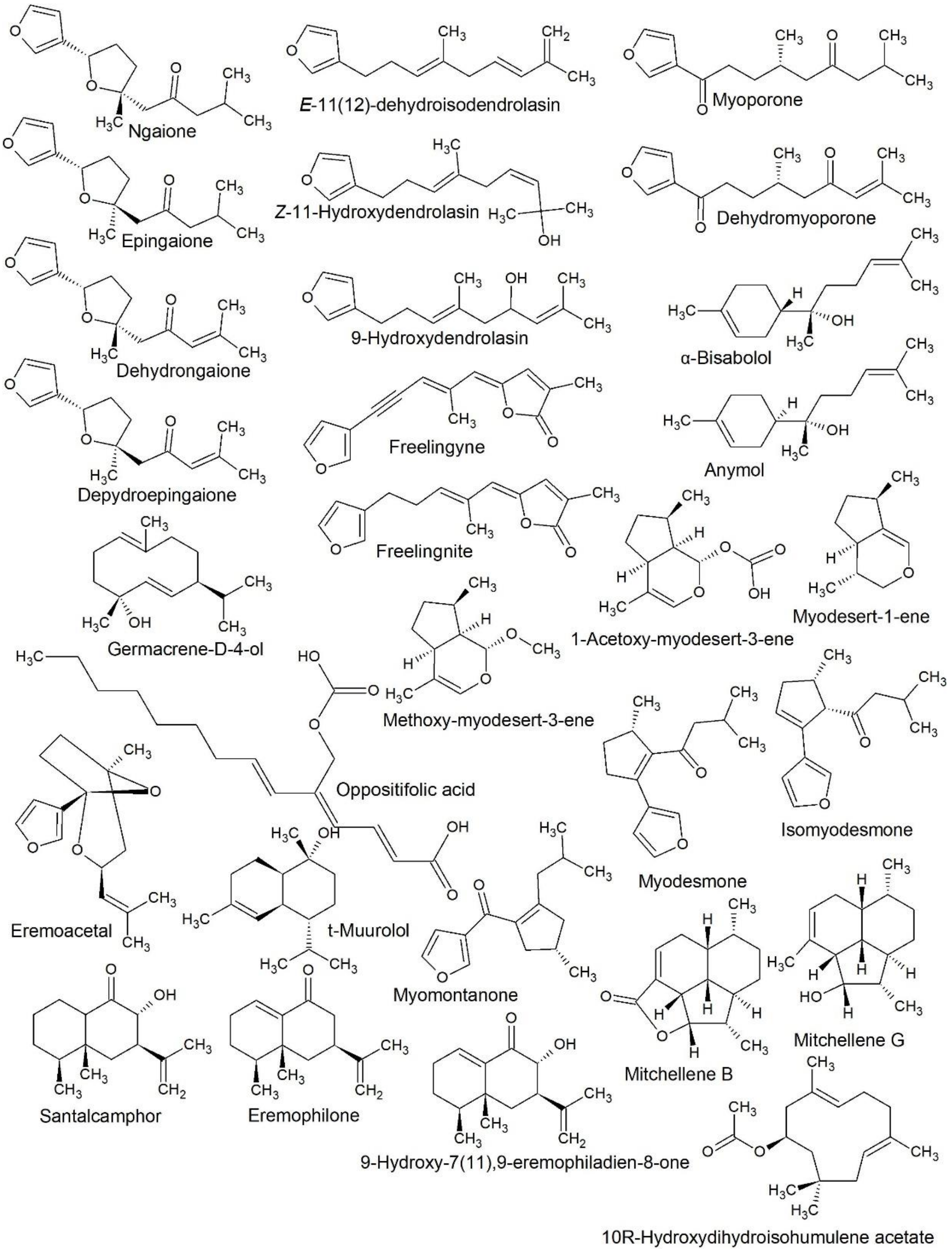

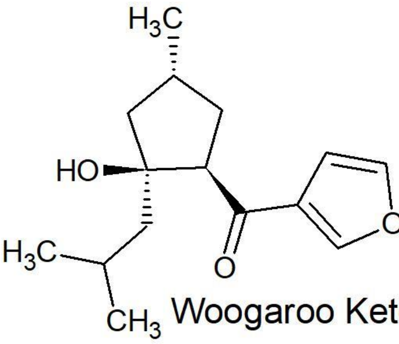
Woogaroo Ketol

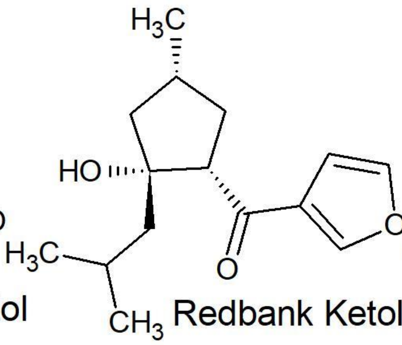
Redbank Ketol

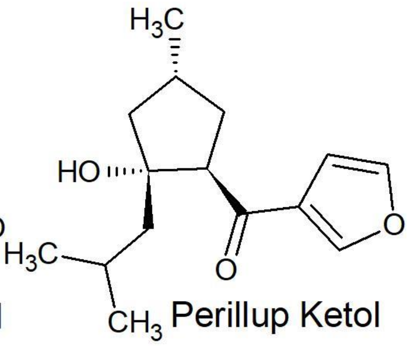
Perillup Ketol

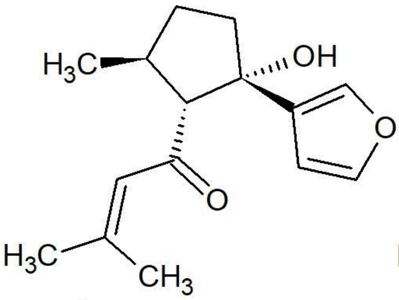
Carney's Ketol

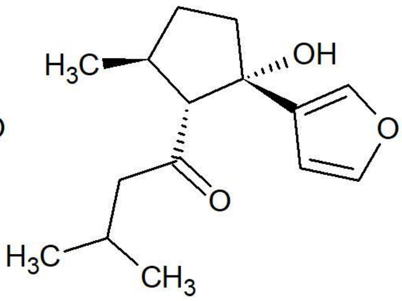
Kindon Ketol

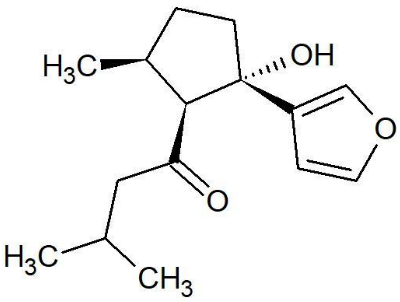
Carr's Ketol

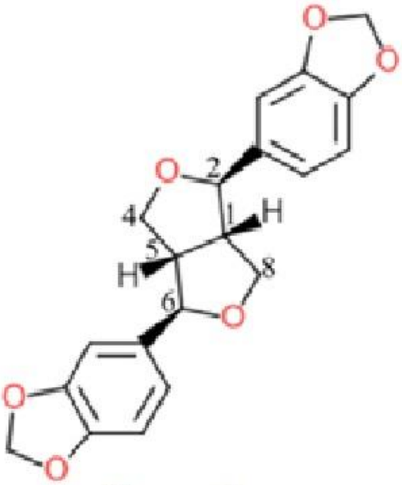
Sesamin

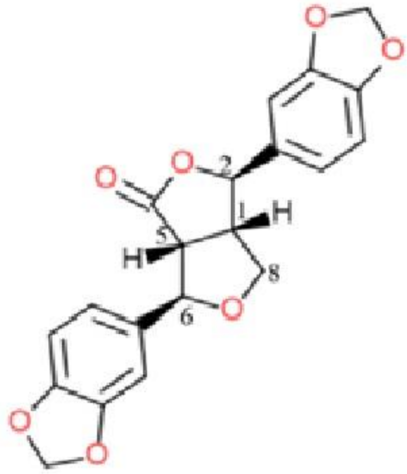
Apoptosin

Supplementary Files – A4; EI mass spectral data (images) of selected unknown compounds

E. bowmannii Unknows

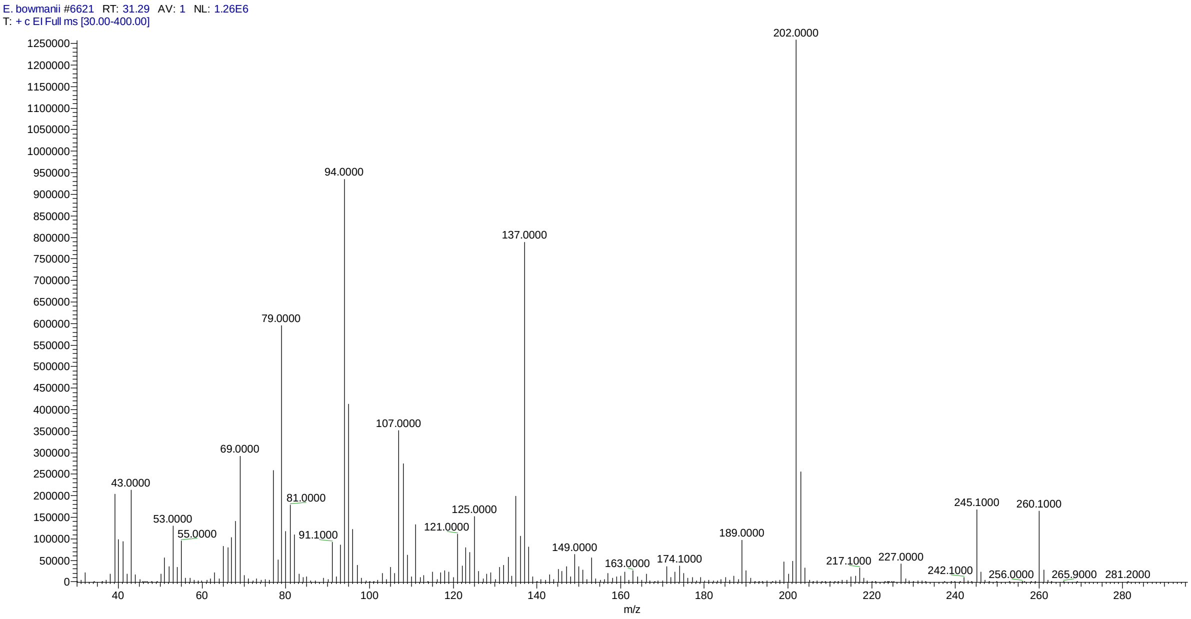

*Figure A4-1 - Electron impact mass spectrum of **Unknown From E. bowmannii**, DB5 column.*

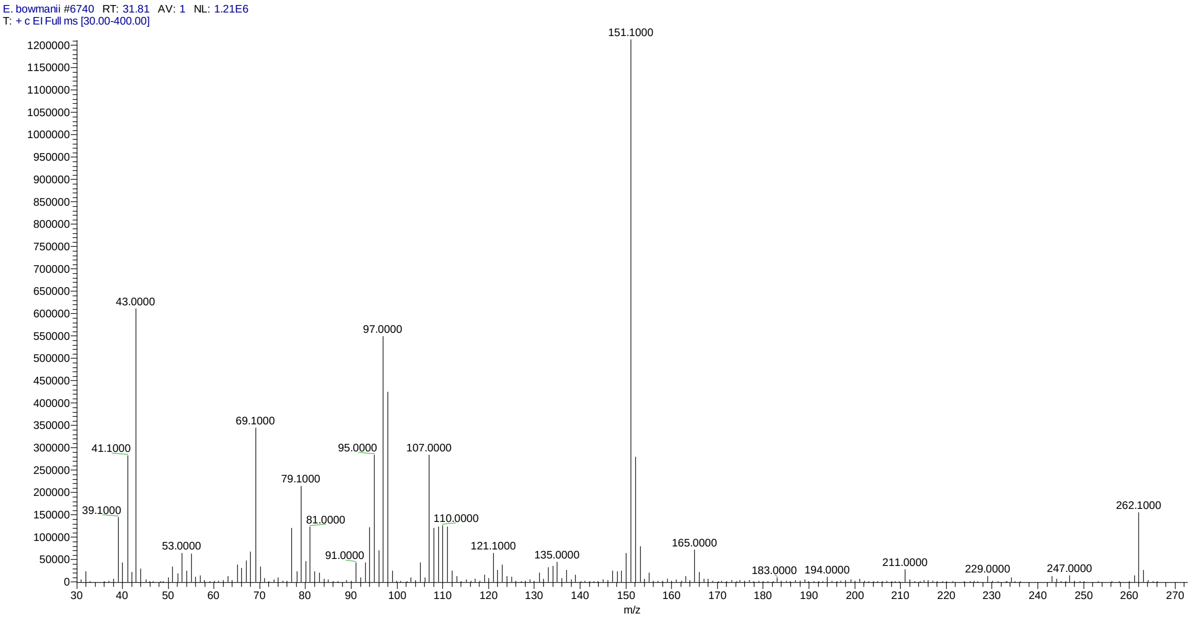

*Figure A4-2 - Electron impact mass spectrum of **Unknown From E. bowmannii**, DB5 column.*

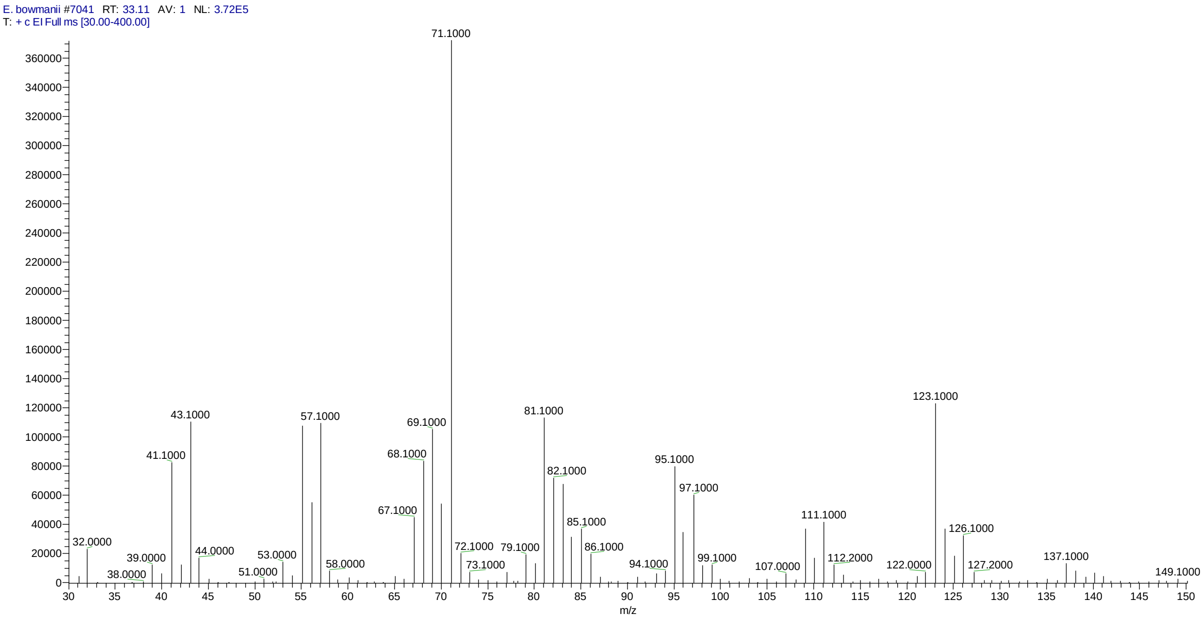

Figure A4-3 - Electron impact mass spectrum of ***Unknown From E. bowmannii****, DB5 column.*

E. oppositifolia Unknows

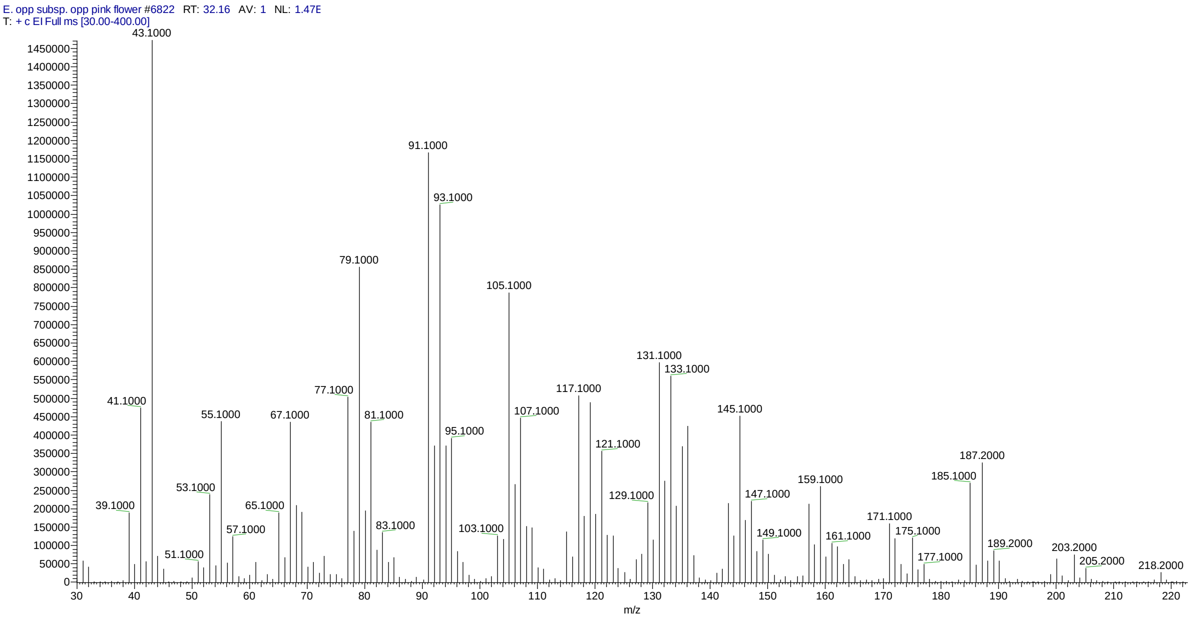

Figure A4-4 - Electron impact mass spectrum of ***Unknown From E. oppositifolia****, DB5 column.*

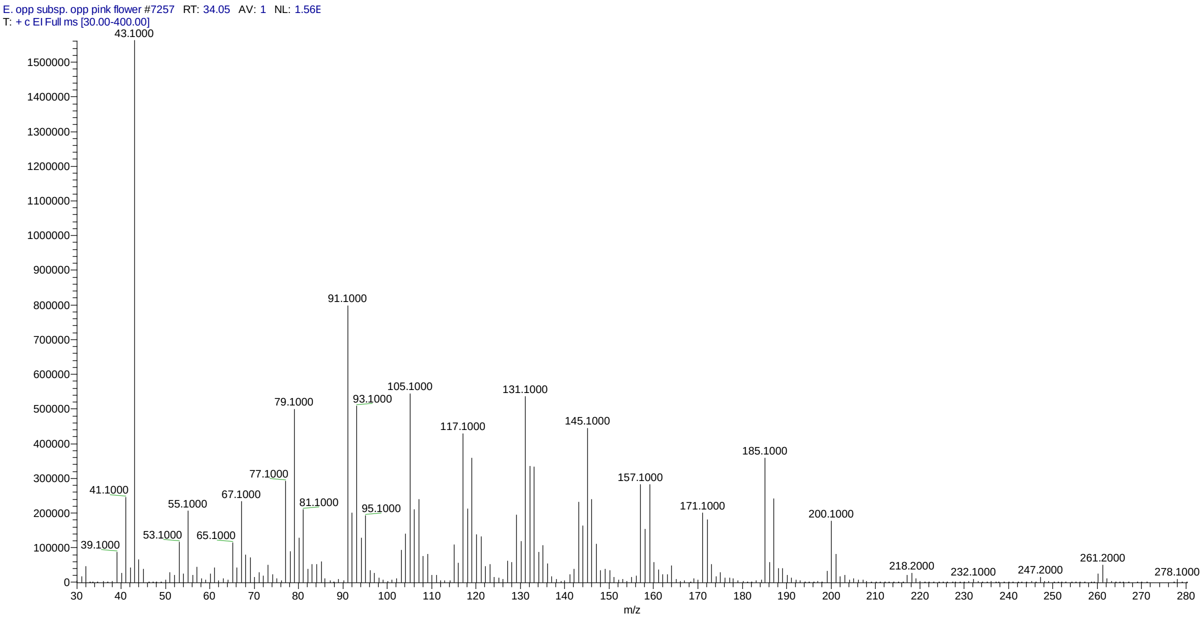

Figure A4-5 - Electron impact mass spectrum of ***Unknown From E. oppositifolia****, DB5 column.*

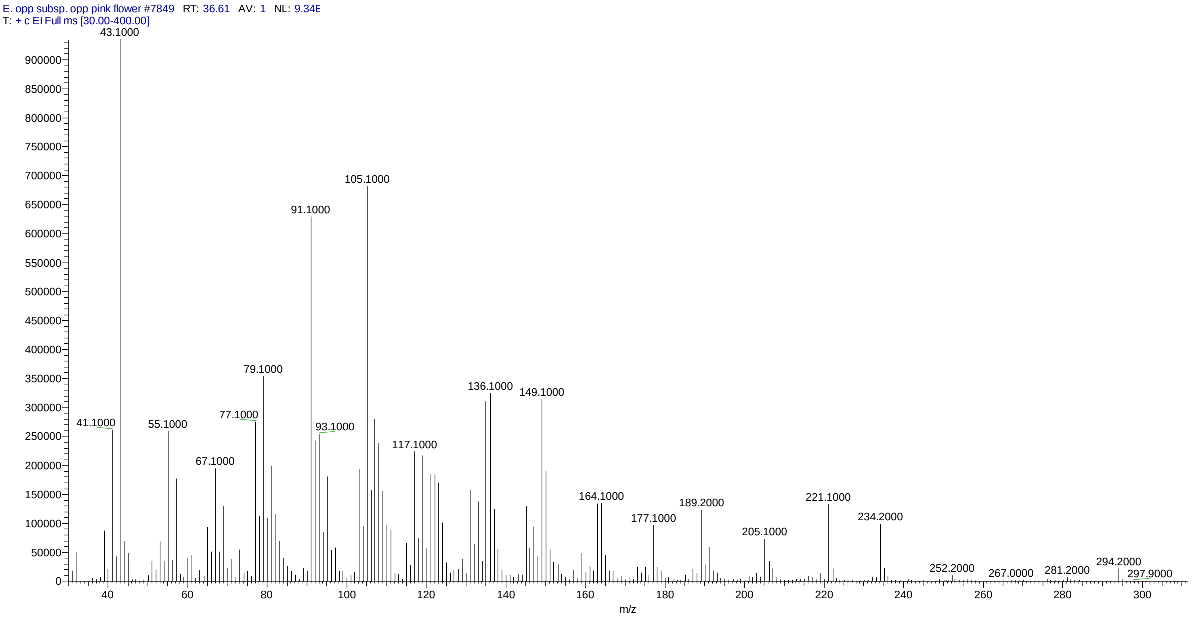

Figure A4-6 - Electron impact mass spectrum of ***Unknown From E. oppositifolia****, DB5 column.*

E. gilesii Unknows

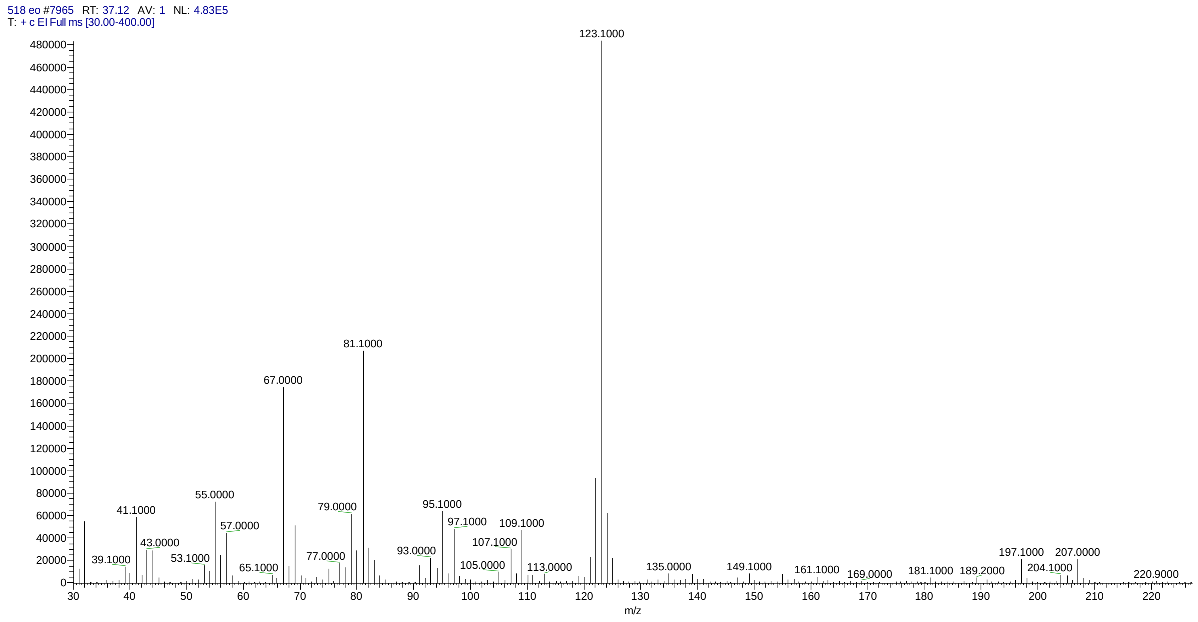

Figure A4-7 - Electron impact mass spectrum of ***Unknown From E.gilesii****, DB5 column.*

Supplementary Files B: The diversity of volatile compounds in Australia’s semi-desert genus *Eremophila* (Scrophulariaceae)

Nicholas J. Sadgrove [1,*], Guillermo F. Paddilla-González[1], Alison Green[1], Moses K. Langat[1], Eduard Mas-Claret[1], Dane Lyddiard[2], Julian Klepp[2], Sarah V.A.-M. Legendre[2], Ben W. Greatrex[2], Graham L. Jones[2], Iskandar M. Ramli[2], Olga Leuner[3], and Eloy Fernandez-Cusimamani[3,*]

[1] Jodrell Science Laboratory, Royal Botanic Gardens Kew, Richmond TW9 3DS, UK; f.padilla@kew.org (G.F.P.-G.), m.langat@kew.org (M.K.L.)
[2] School of Science and Technology and School of Rural Medicine, University of New England, Armidale NSW 2351, Australia; bgreatre@une.edu.au (B.W.G.), gjones2@une.edu.au (G.L.J.)
[3] Department of Crop Sciences and Agroforestry, Faculty of Tropical AgriSciences, Czech University of Life Sciences Prague, Kamýcká 129, 16500 Prague, Czech Republic
* Correspondence: n.sadgrove@kew.org (N.J.S.); eloy@ftz.czu.cz (E.F.-C.); Tel.: +44-7857569823 (N.J.S.);
+420-224382183 (E.F.-C.)

Document Structure

Supplementary Files B – 1H and 13C NMR images of rare or new compounds from species of *Eremophila* (Scrophulariaceae)

9-Hydroxy-7(11),9-eremophiladien-8-one 10 1 "C:\Users\nmrsu\OneDrive - The Royal Botanic Gardens, Kew\Desktop\Eremophila NMR"

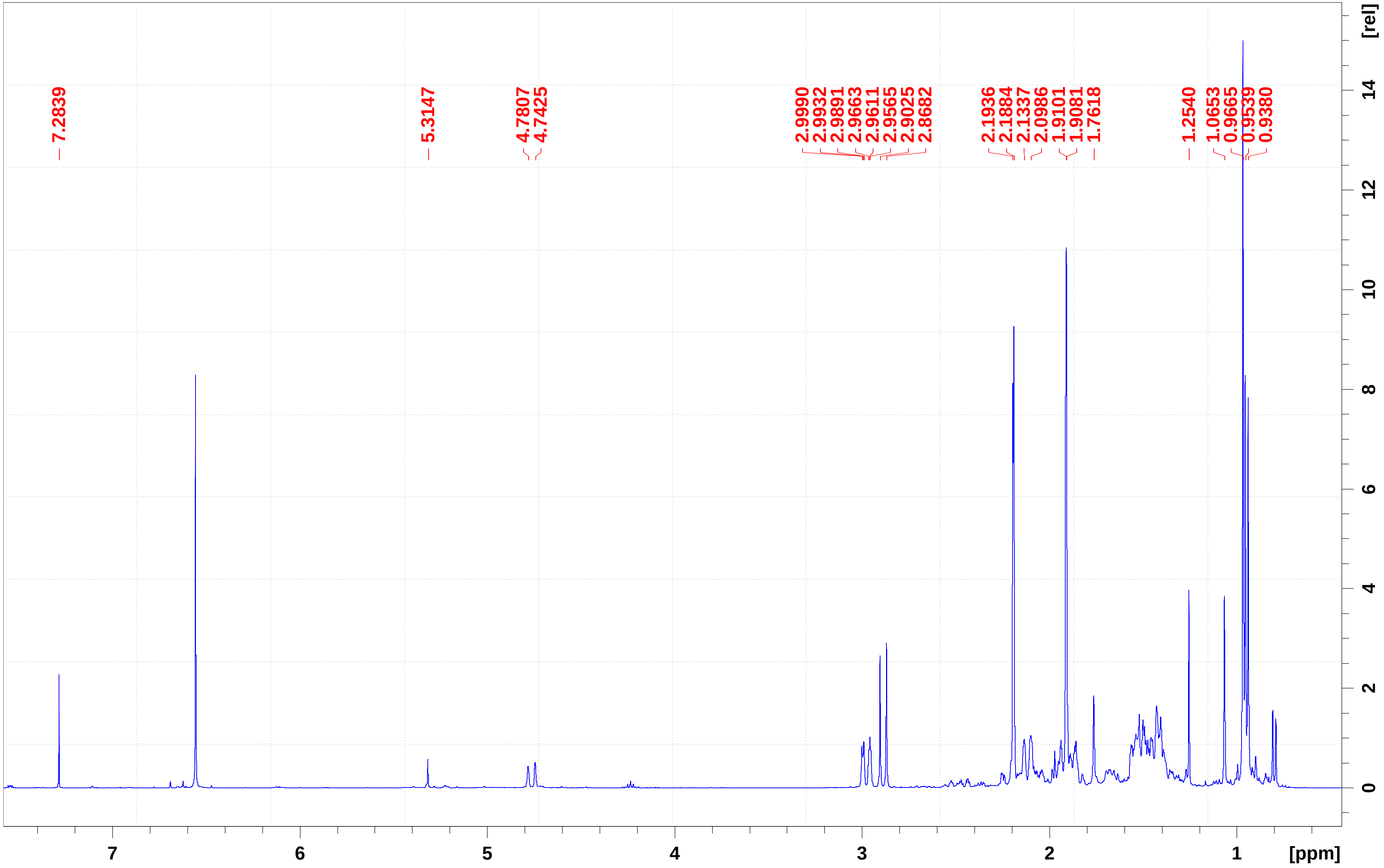

9-Hydroxy-7(11),9-eremophiladien-8-one 11 1 "C:\Users\nmrsu\OneDrive - The Royal Botanic Gardens, Kew\Desktop\Eremophila NMR"

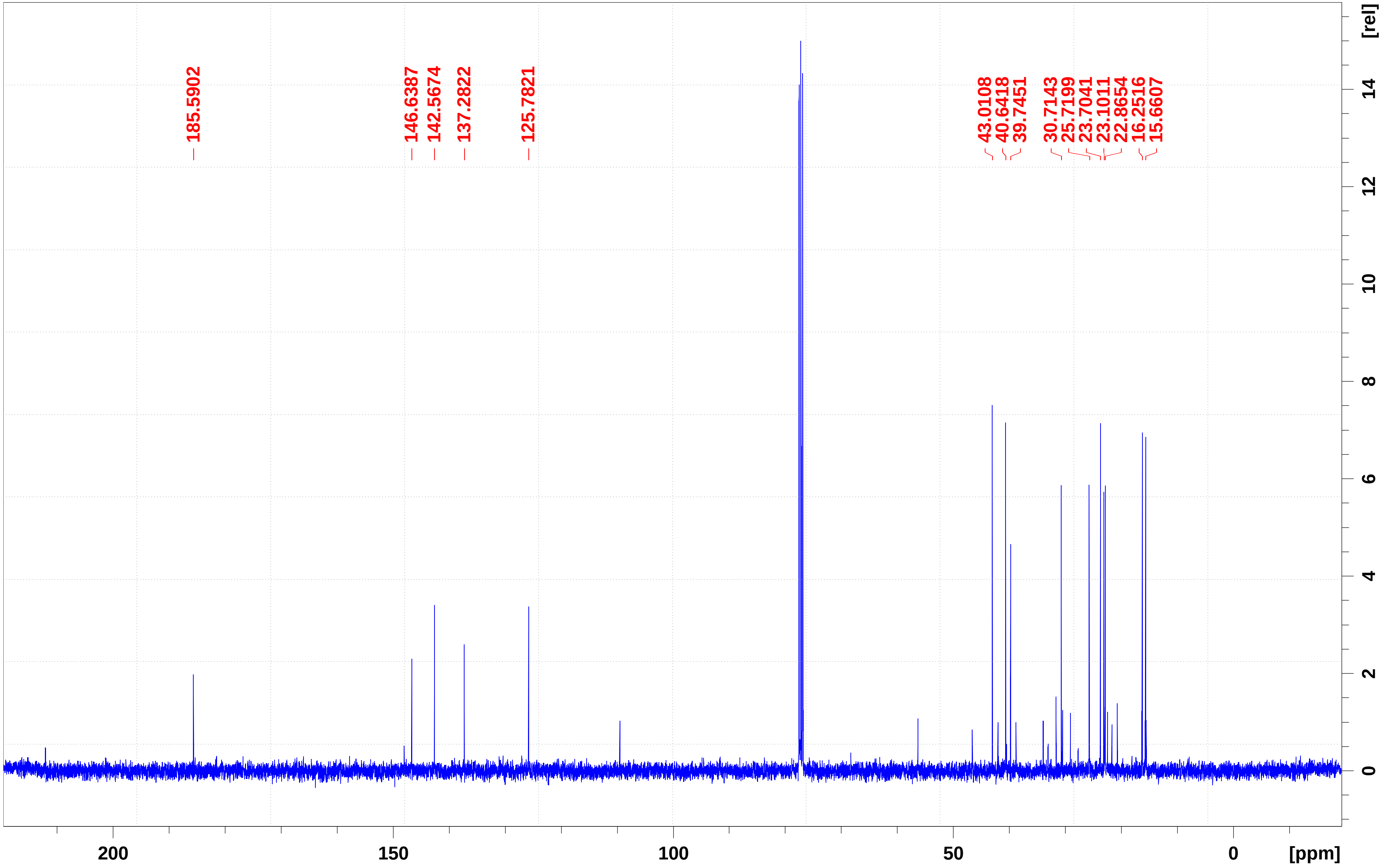

9-Hydroxydendrolasin 1 1 "C:\Users\nmrsu\OneDrive - The Royal Botanic Gardens, Kew\Desktop\Eremophila NMR"

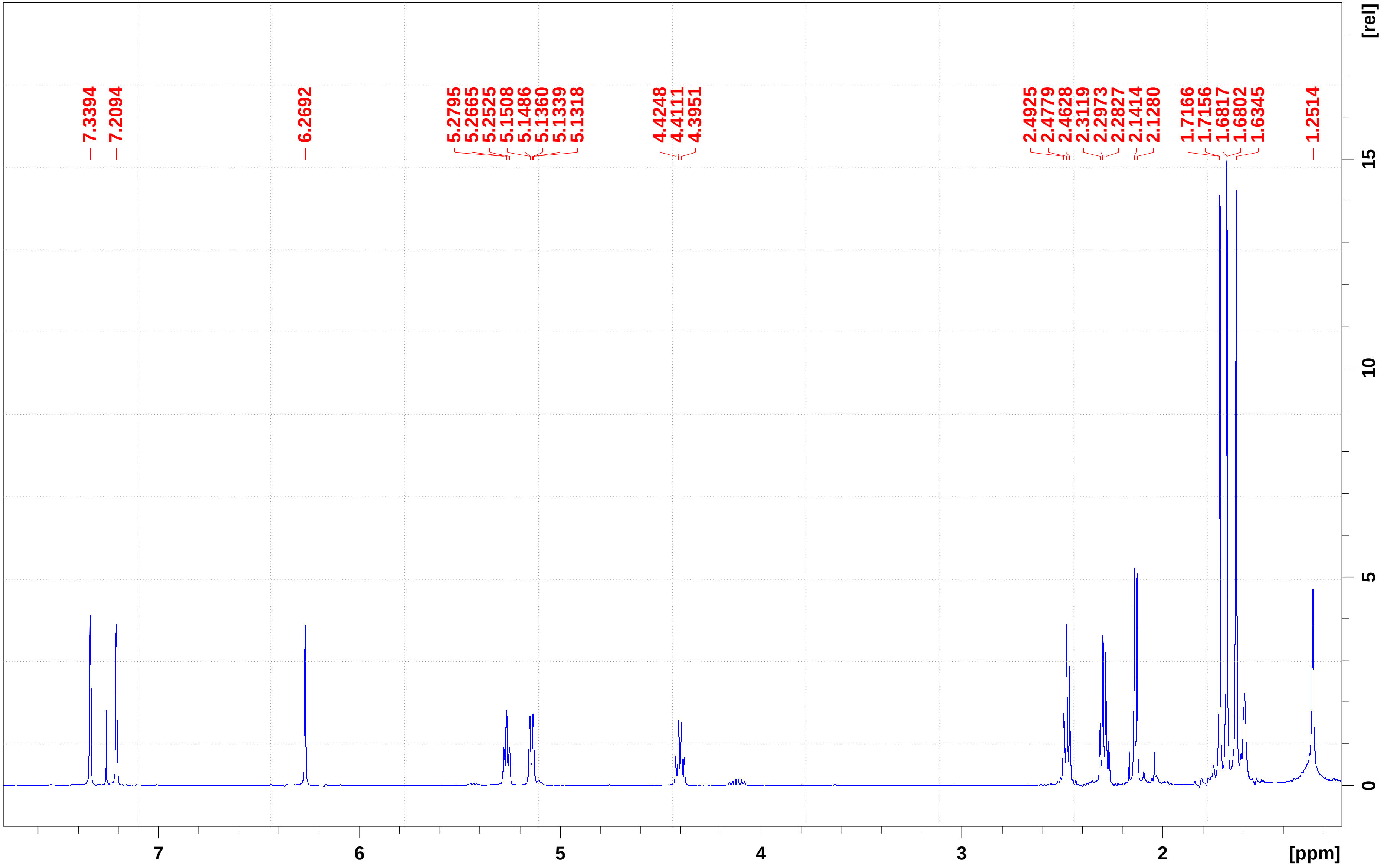

9-Hydroxydendrolasin 2 1 "C:\Users\nmrsu\OneDrive - The Royal Botanic Gardens, Kew\Desktop\Eremophila NMR"

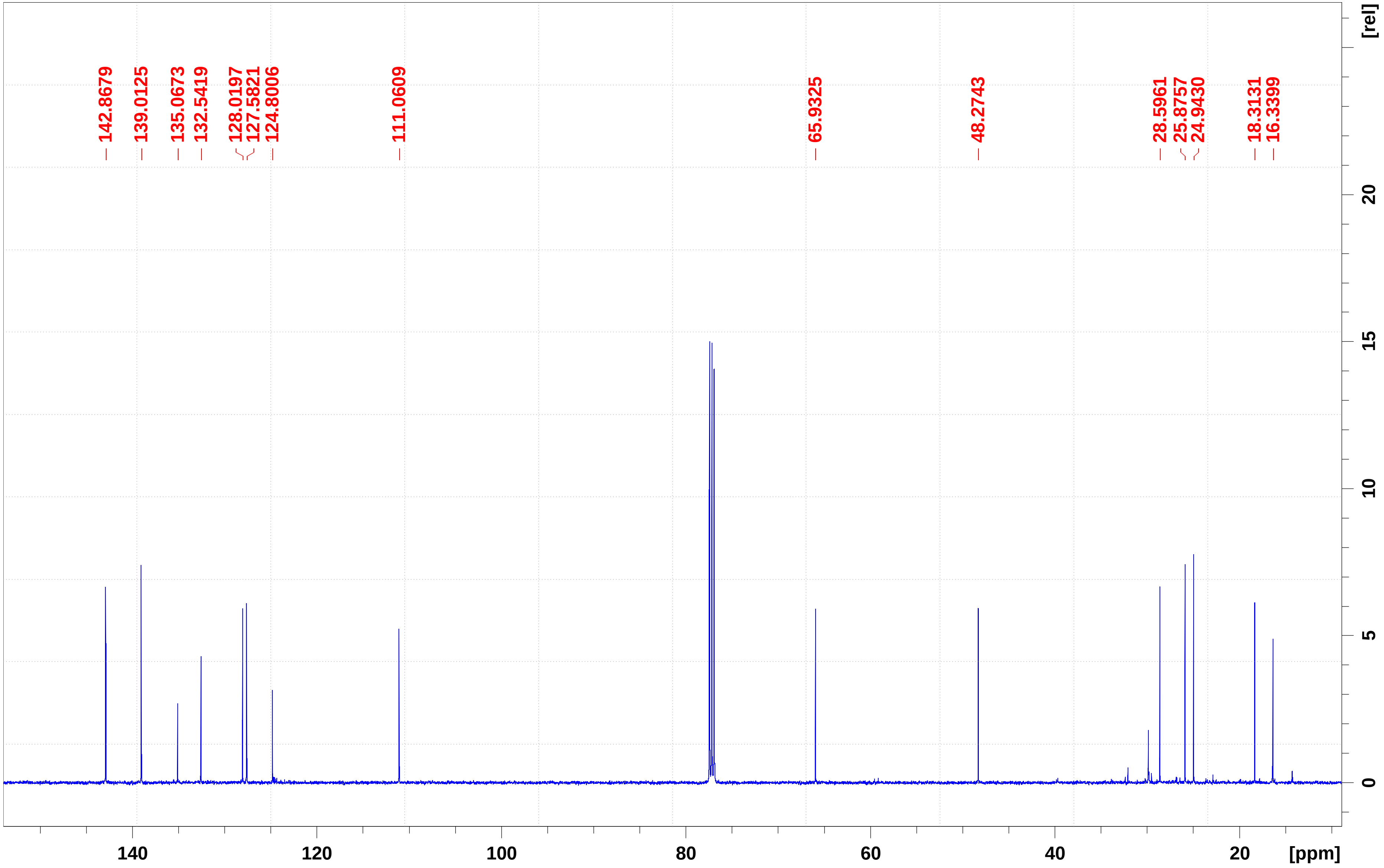

"10-Hydroxyisohumulene acetate" 1 1 "C:\Users\nmrsu\OneDrive - The Royal Botanic Gardens, Kew\Desktop\Eremophila NMR"

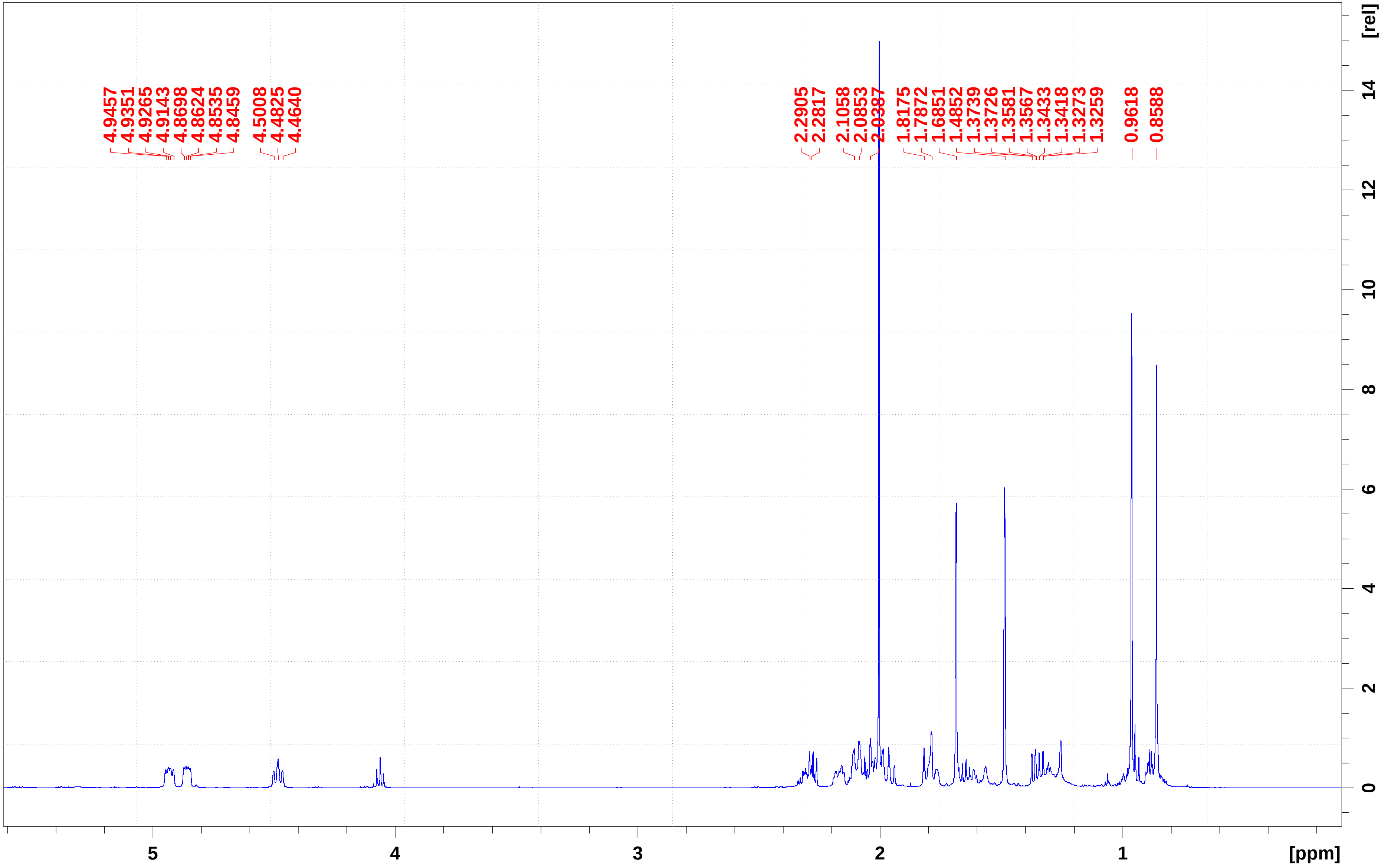

"10-Hydroxyisohumulene acetate" 2 1 "C:\Users\nmrsu\OneDrive - The Royal Botanic Gardens, Kew\Desktop\Eremophila NMR"

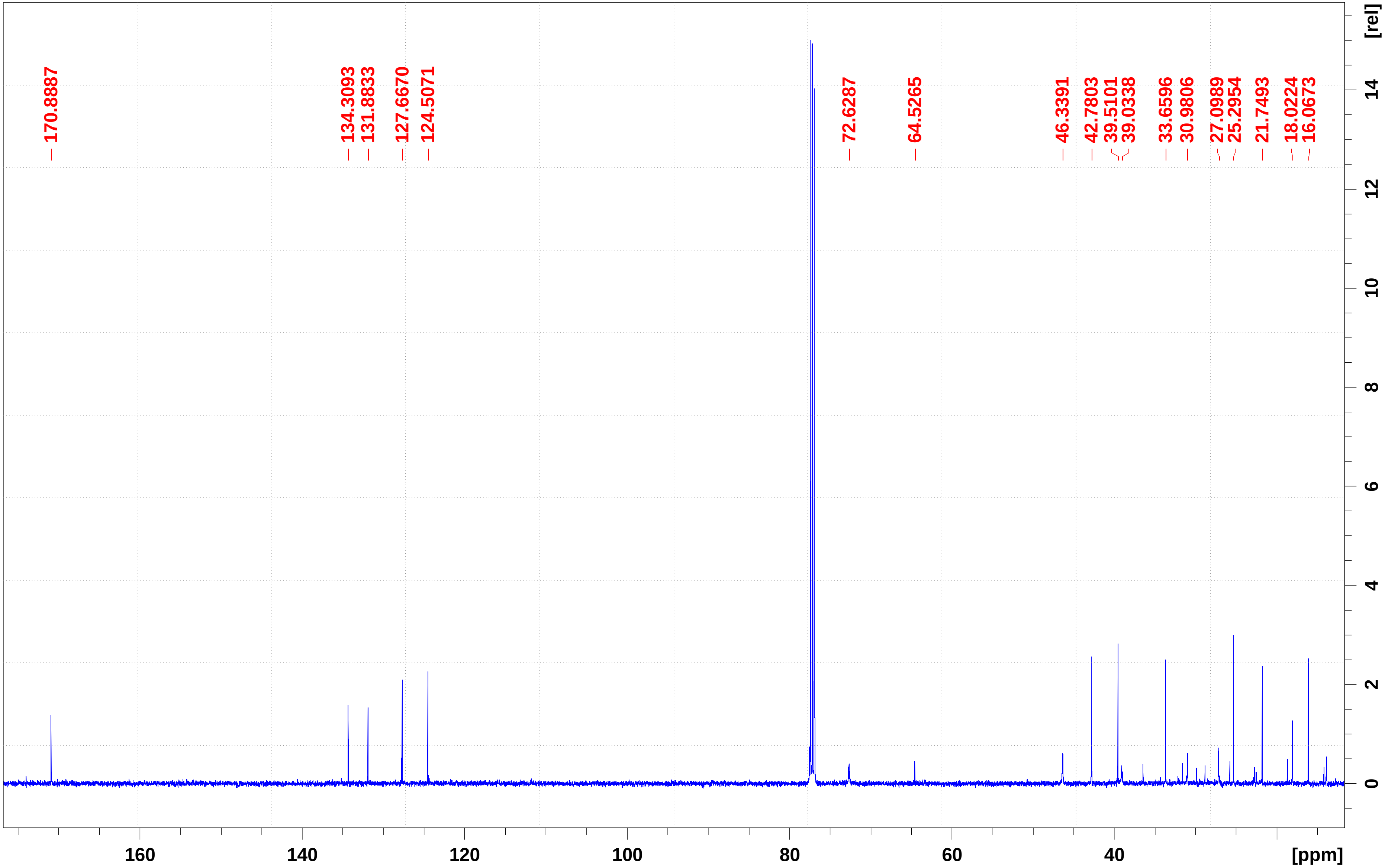

11,12-Dehydroisodendrolasin 3 1 "C:\Users\nmrsu\OneDrive - The Royal Botanic Gardens, Kew\Desktop\Eremophila NMR"

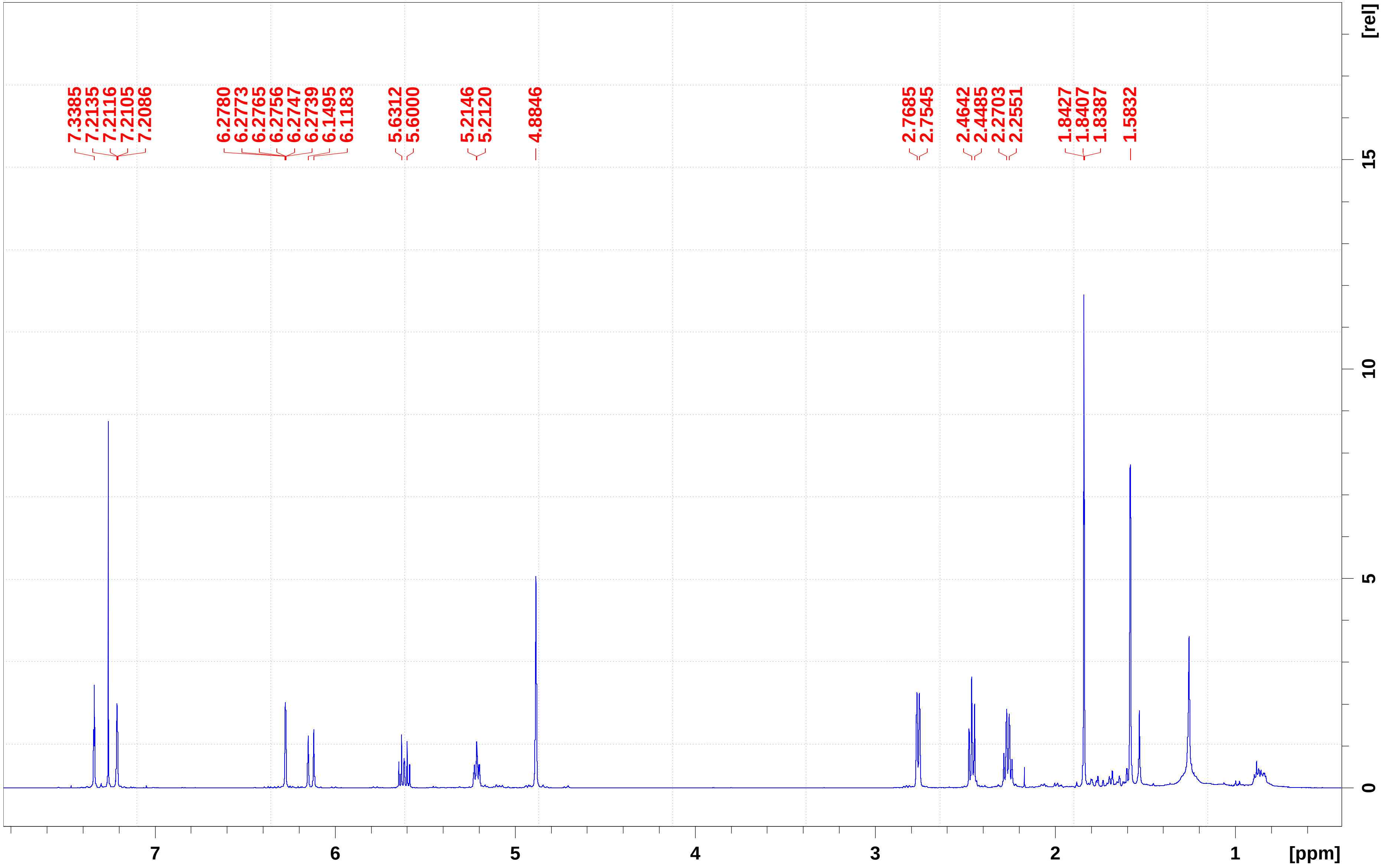

11,12-Dehydroisodendrolasin 4 1 "C:\Users\nmrsu\OneDrive - The Royal Botanic Gardens, Kew\Desktop\Eremophila NMR"

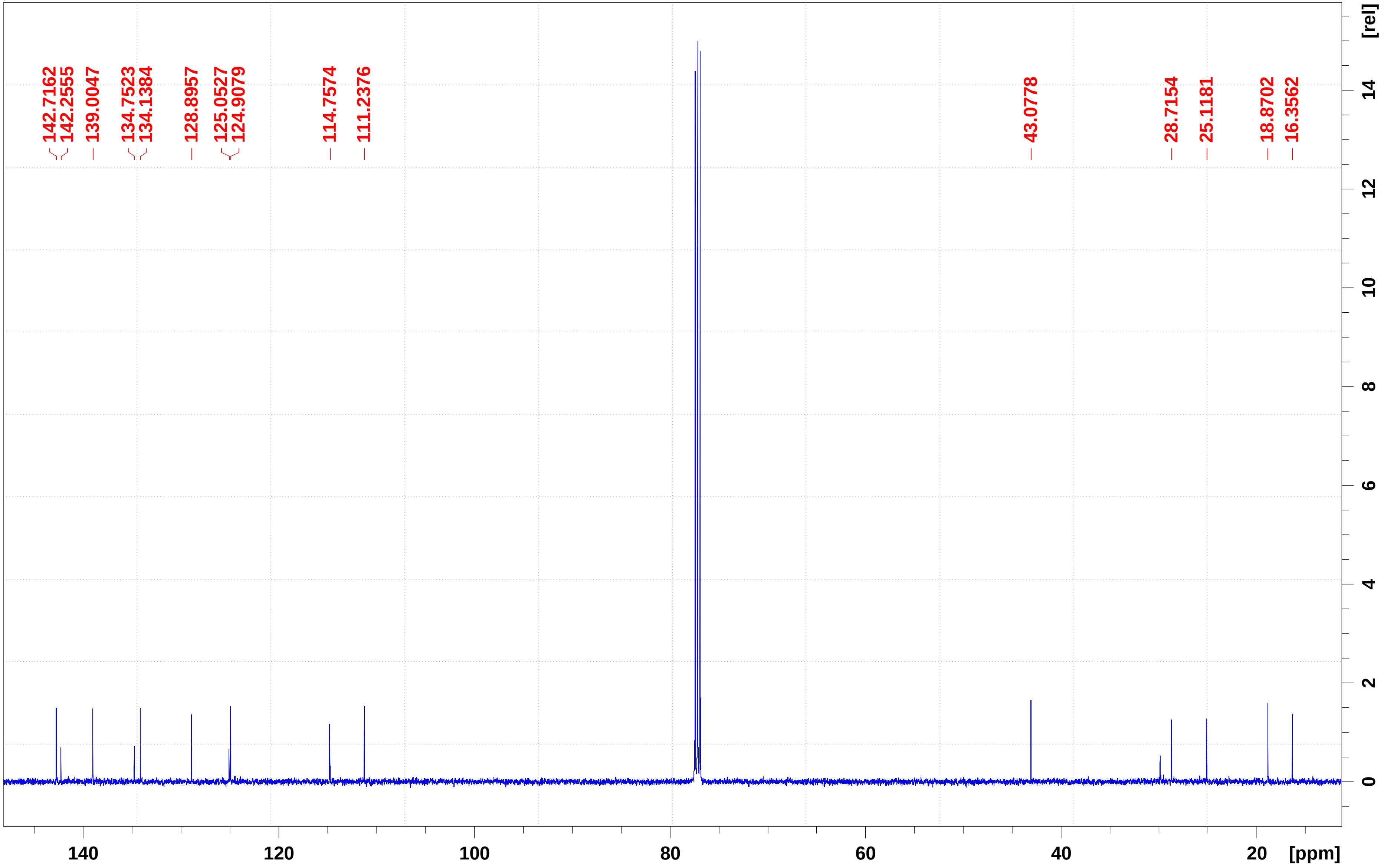

11-Hydroxyisodendrolasin 10 1 "C:\Users\nmrsu\OneDrive - The Royal Botanic Gardens, Kew\Desktop\Eremophila NMR"

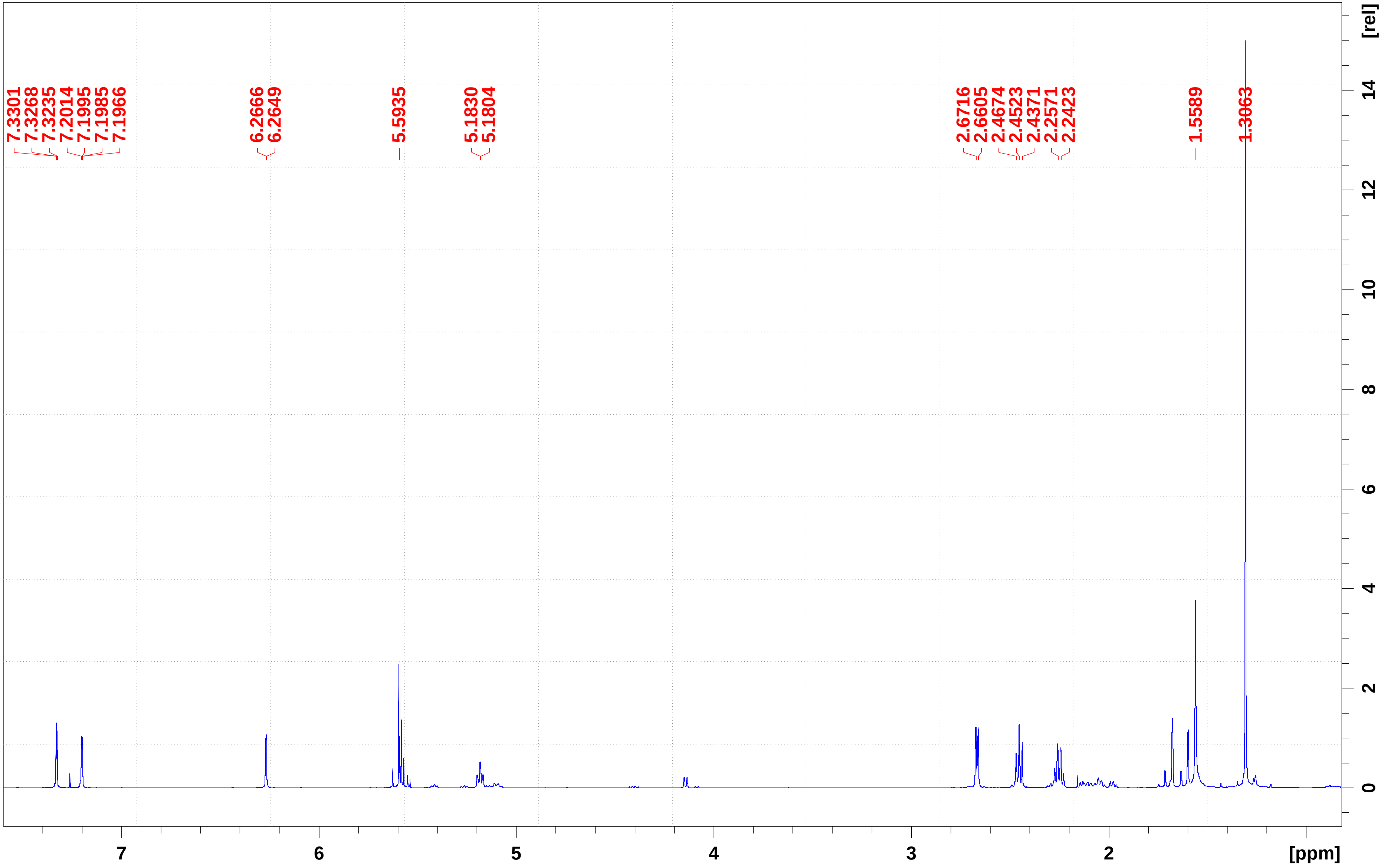

11-Hydroxyisodendrolasin 11 1 "C:\Users\nmrsu\OneDrive - The Royal Botanic Gardens, Kew\Desktop\Eremophila NMR"

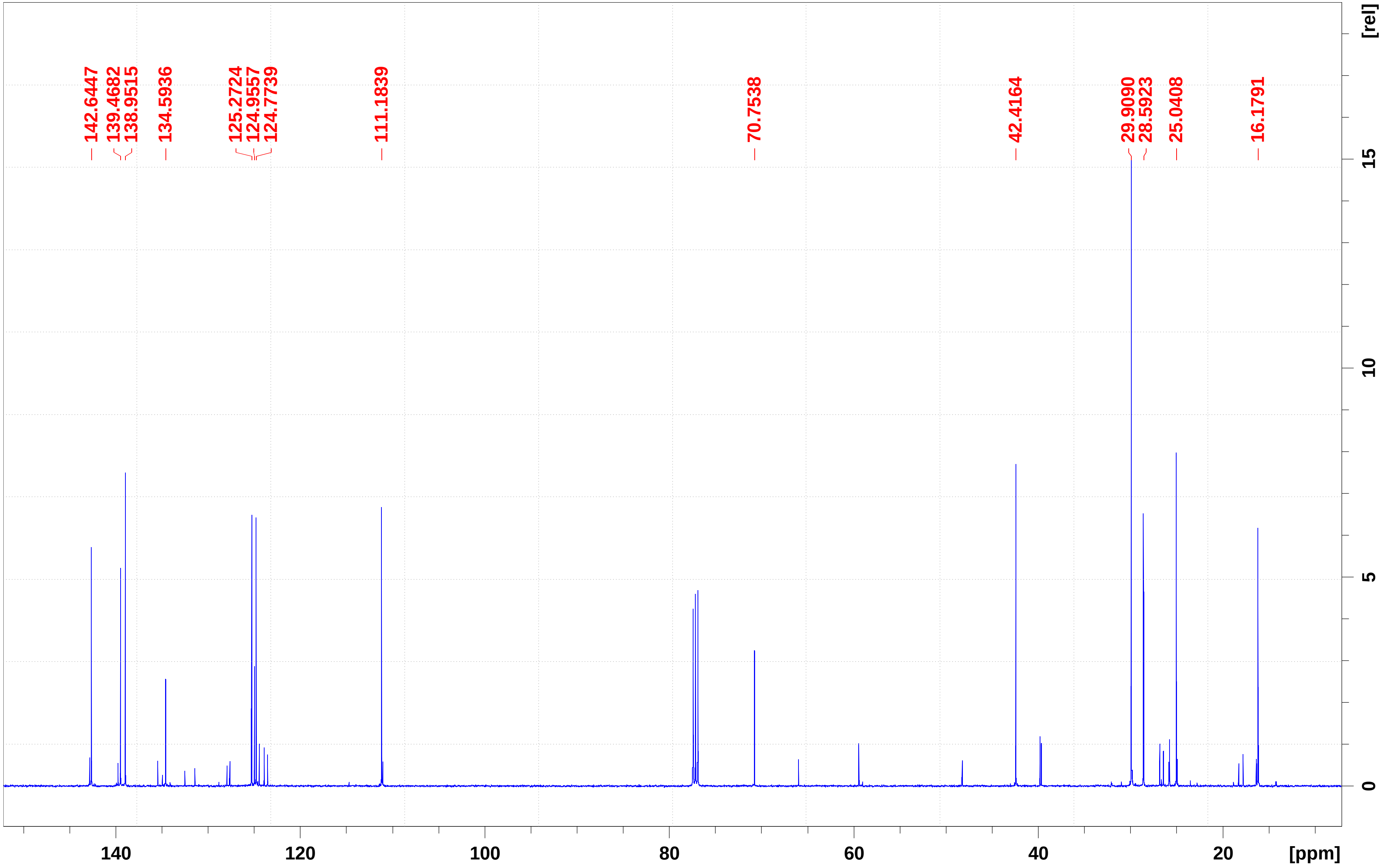

Acetoxymyodesert-3-ene 1 1 "C:\Users\nmrsu\OneDrive - The Royal Botanic Gardens, Kew\Desktop\Eremophila NMR"

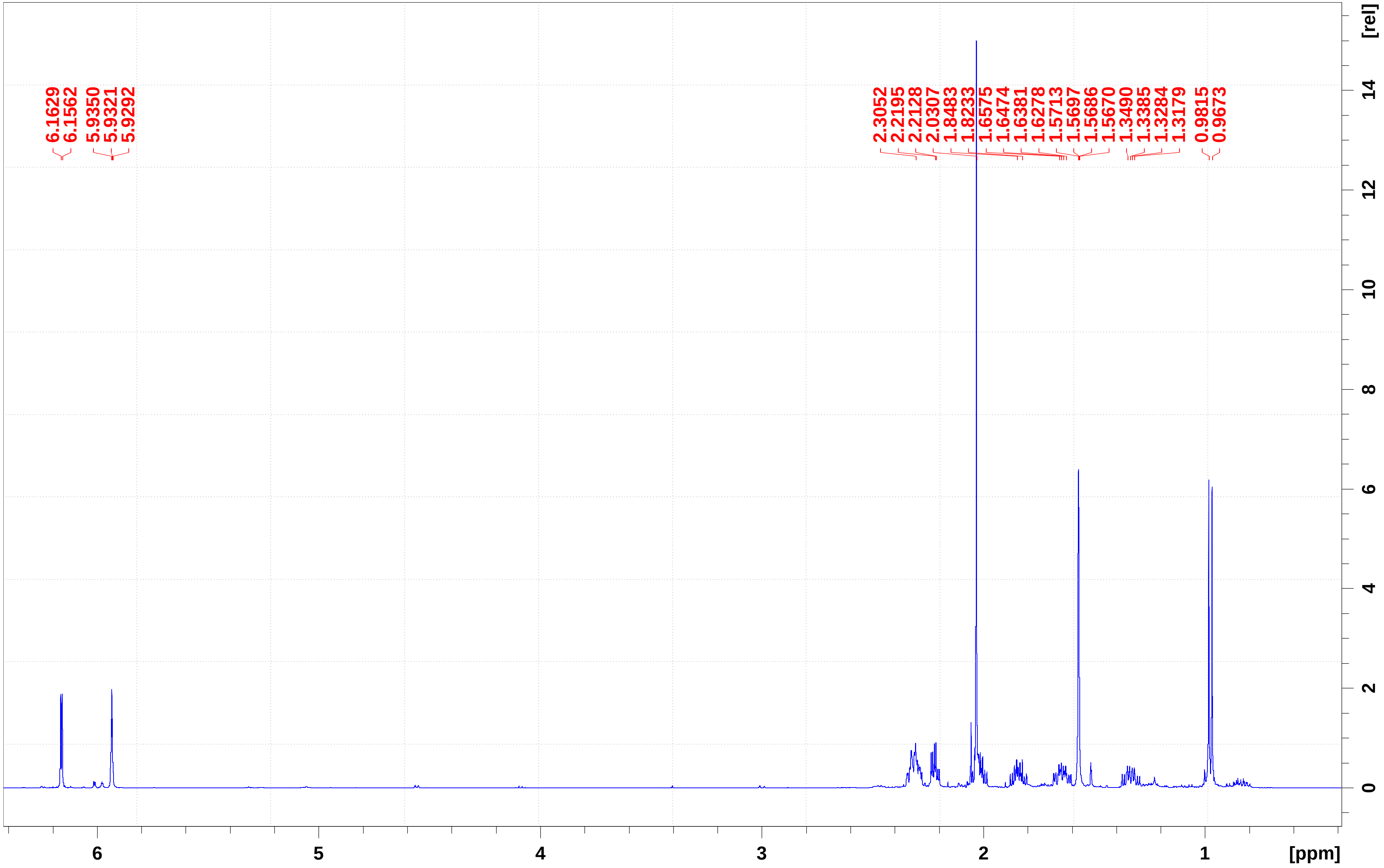

Acetoxymyodesert-3-ene 2 1 "C:\Users\nmrsu\OneDrive - The Royal Botanic Gardens, Kew\Desktop\Eremophila NMR"

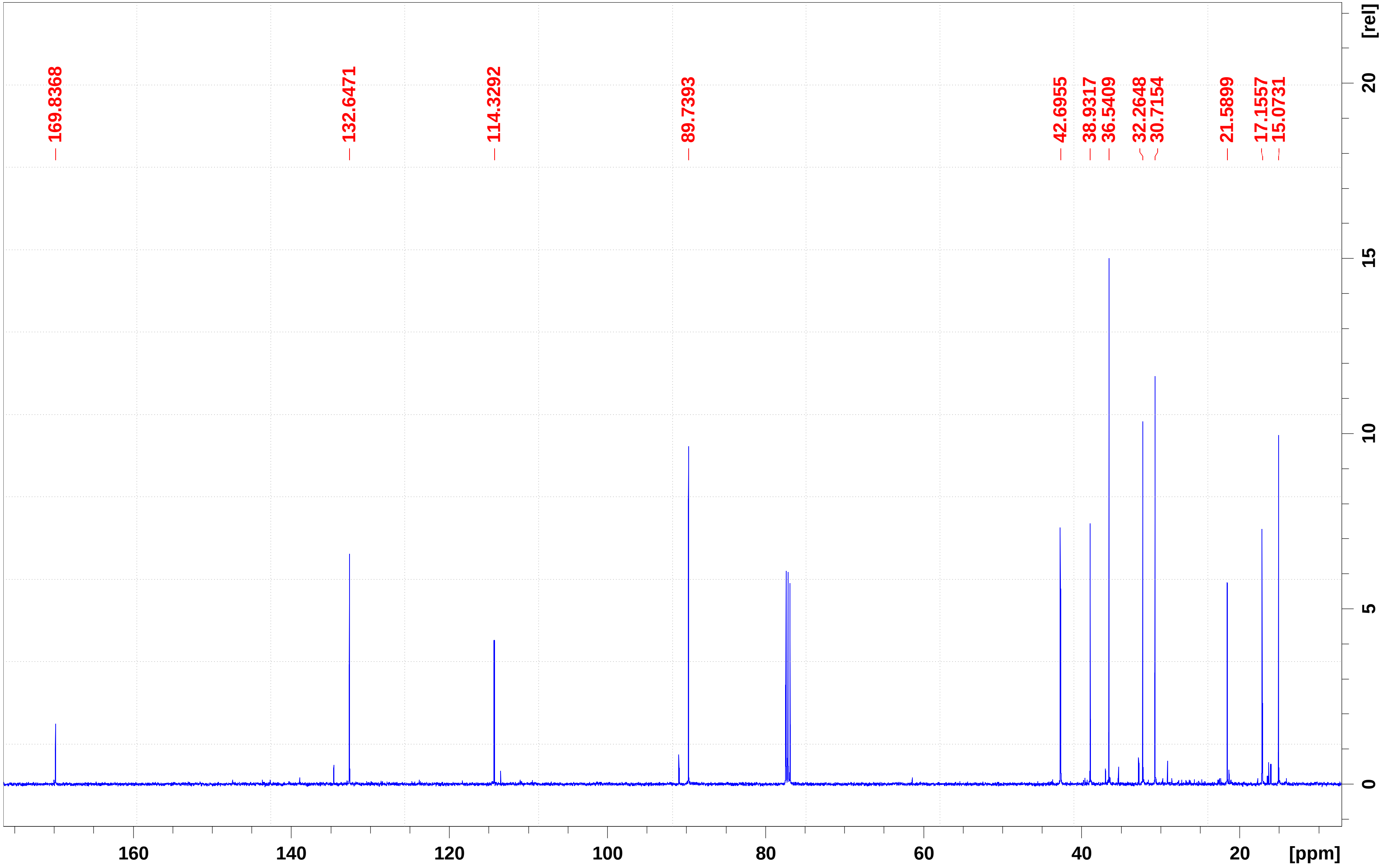

Anymol 1 1 "C:\Users\nmrsu\OneDrive - The Royal Botanic Gardens, Kew\Desktop\Eremophila NMR"

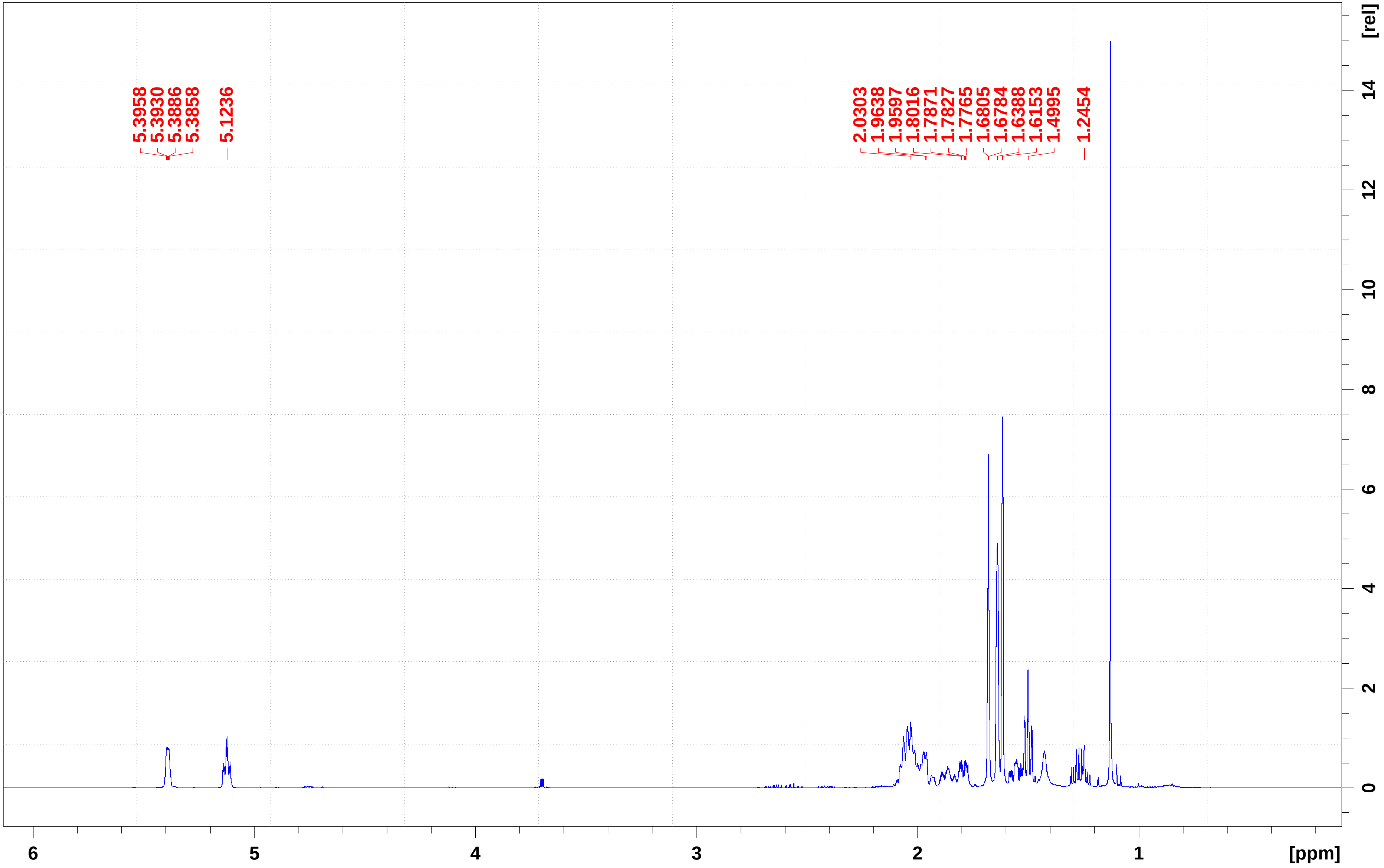

Anymol 2 1 "C:\Users\nmrsu\OneDrive - The Royal Botanic Gardens, Kew\Desktop\Eremophila NMR"

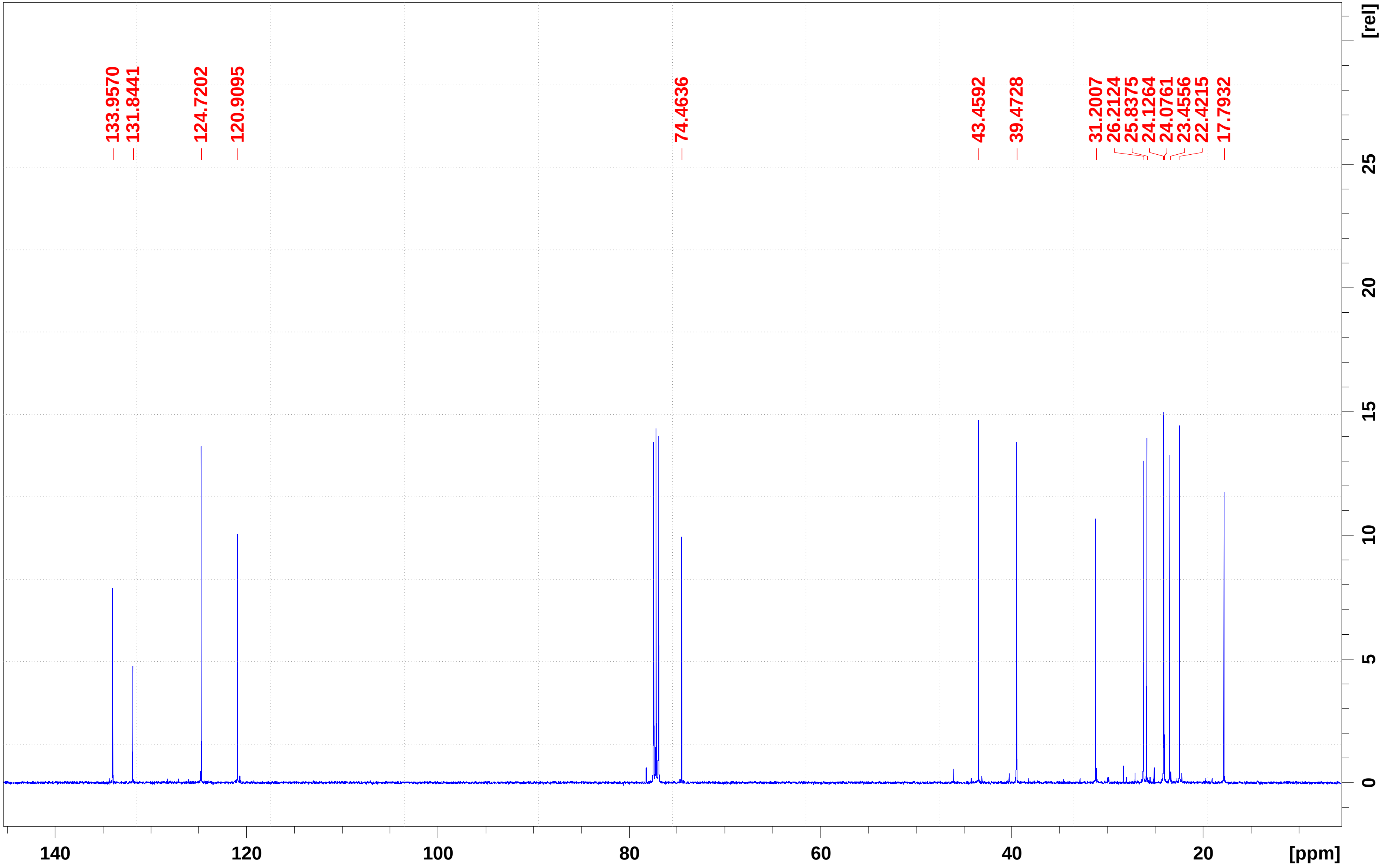

Dehydromyoporone 1 1 "C:\Users\nmrsu\OneDrive - The Royal Botanic Gardens, Kew\Desktop\Eremophila NMR"

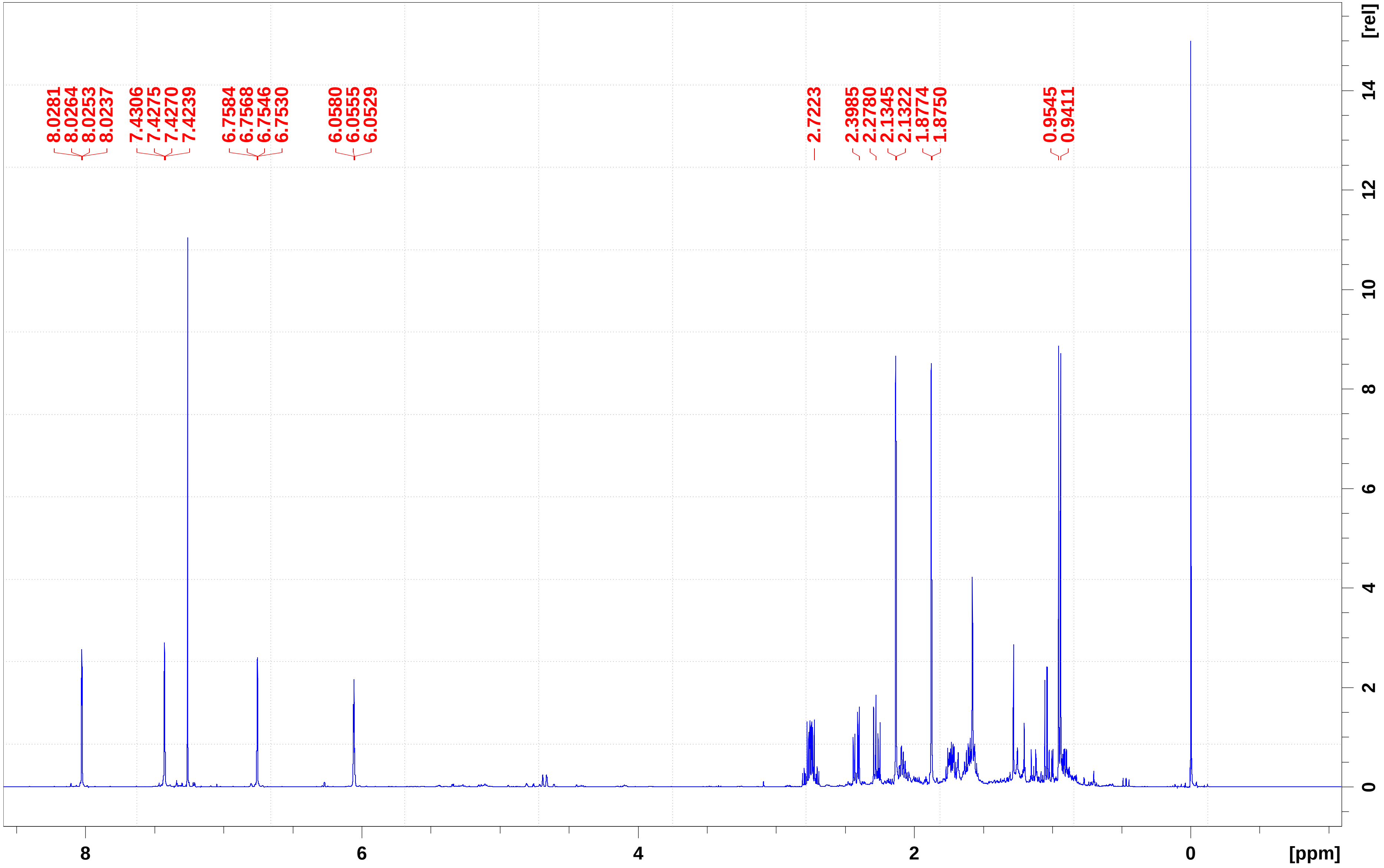

Dehydromyoporone 2 1 "C:\Users\nmrsu\OneDrive - The Royal Botanic Gardens, Kew\Desktop\Eremophila NMR"

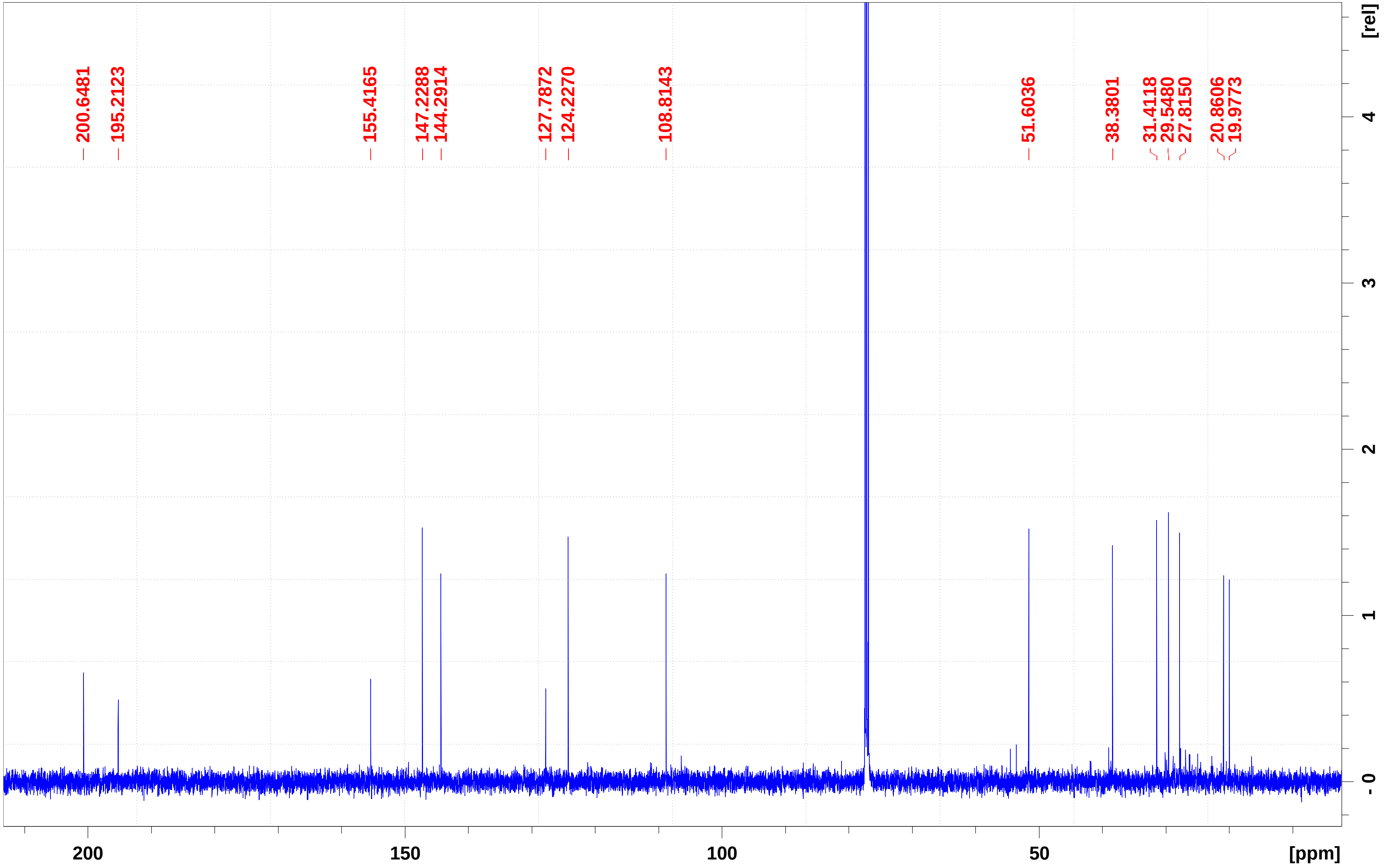

Dehydrongaione 1 1 "C:\Users\nmrsu\OneDrive - The Royal Botanic Gardens, Kew\Desktop\Eremophila NMR\Dihydrongaione"

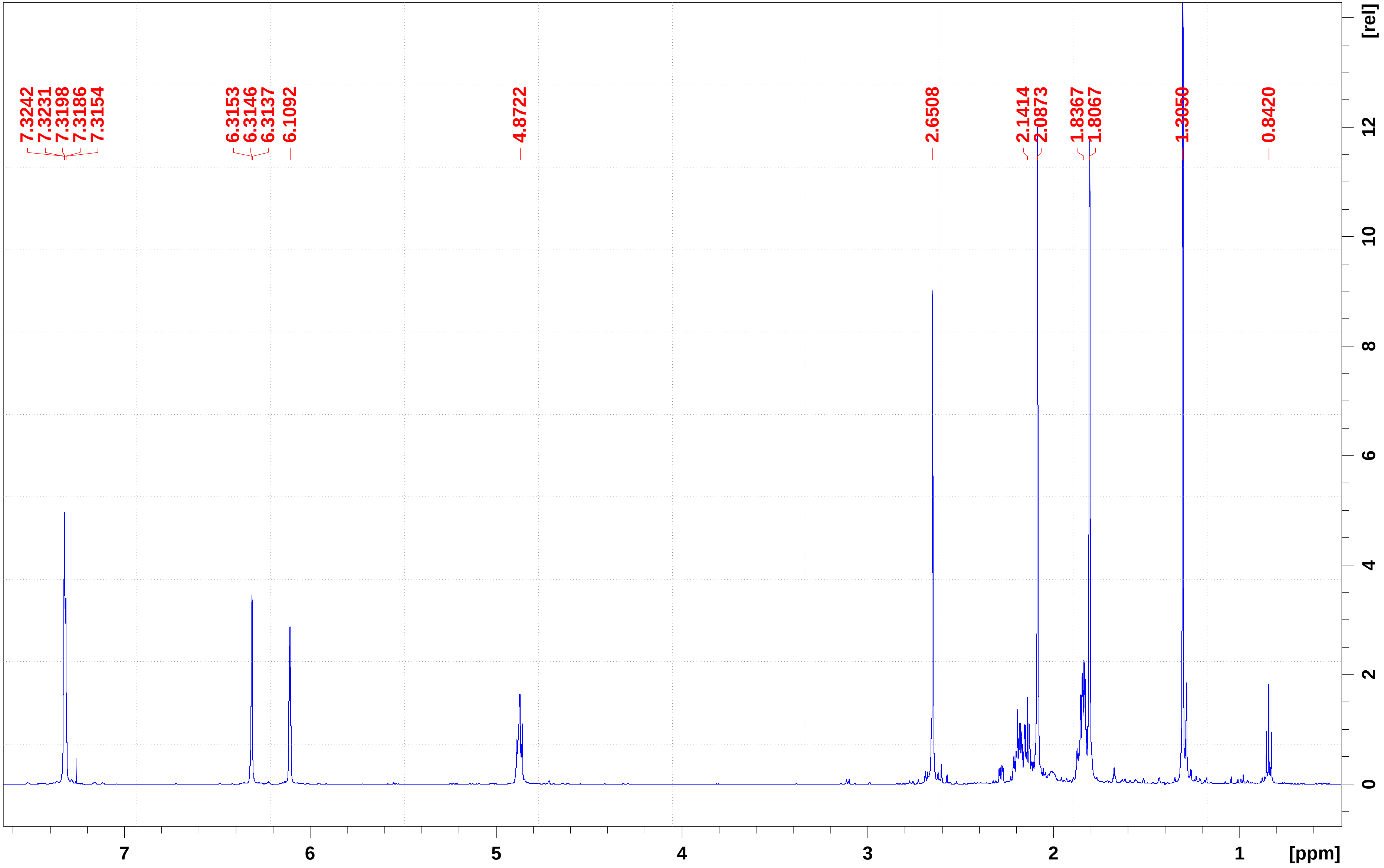

Dehydrongaione 2 1 "C:\Users\nmrsu\OneDrive - The Royal Botanic Gardens, Kew\Desktop\Eremophila NMR\Dihydrongaione"

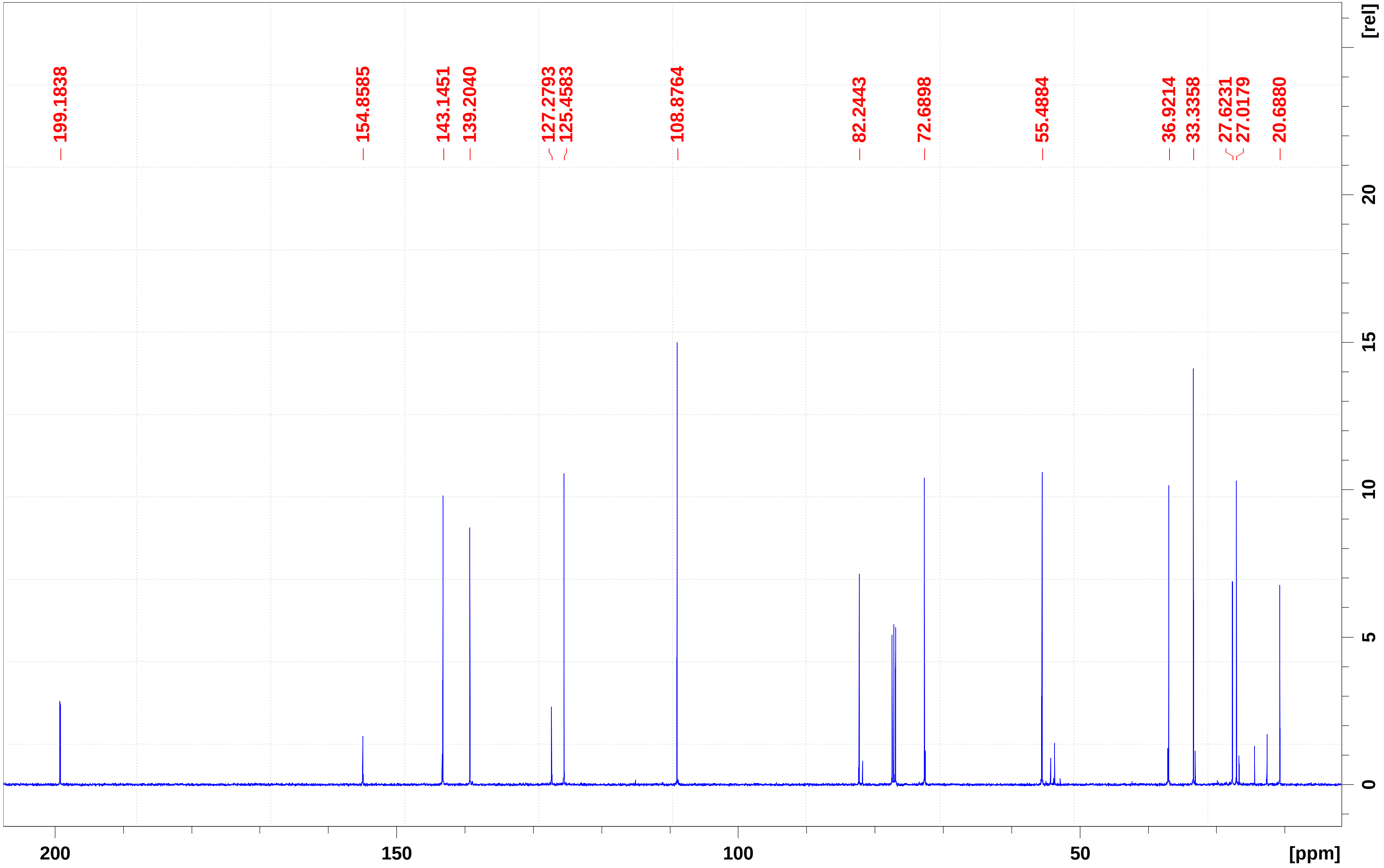

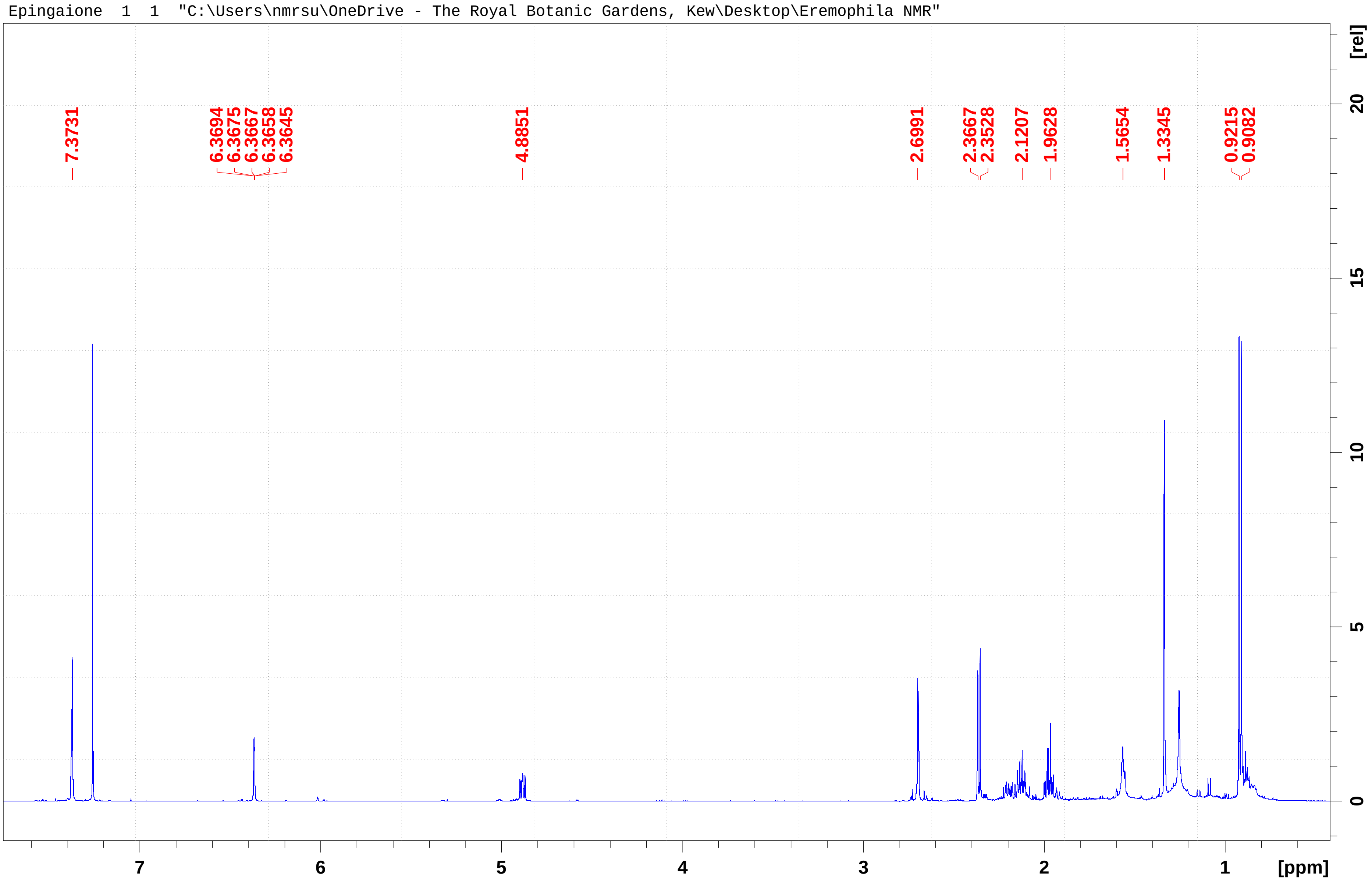
Epingaione 1 1 "C:\Users\nmrsu\OneDrive - The Royal Botanic Gardens, Kew\Desktop\Eremophila NMR"
7.3731
6.3694
6.3675
6.3667
6.3658
6.3645
4.8851
2.6991
2.3667
2.3528
2.1207
1.9628
1.5654
1.3345
0.9215
0.9082
[rel]
20
15
10
5
0
7
6
5
4
3
2
1
[ppm]

Epingaione 2 1 "C:\Users\nmrsu\OneDrive - The Royal Botanic Gardens, Kew\Desktop\Eremophila NMR"

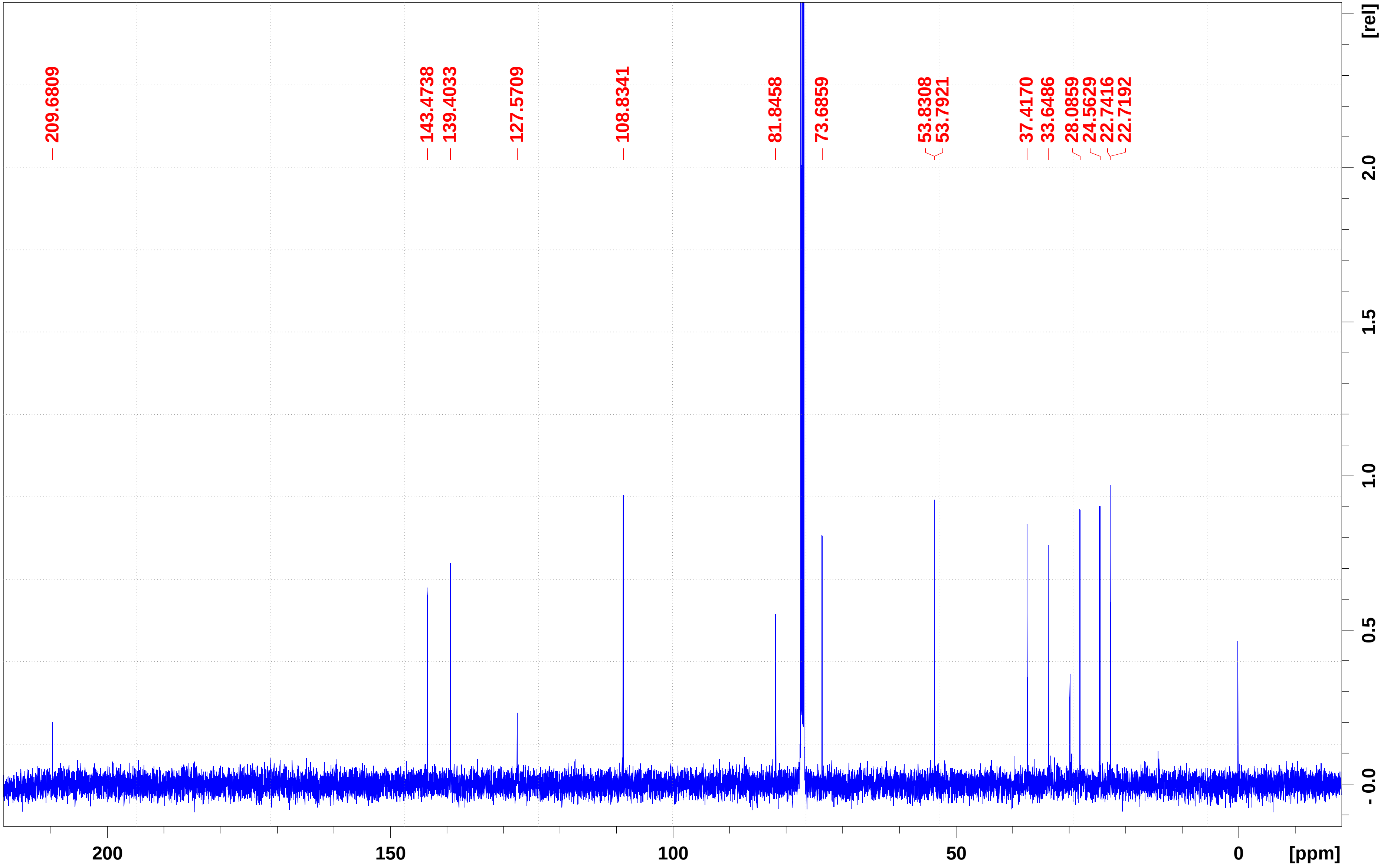

Eremophilone 10 1 "C:\Users\nmrsu\OneDrive - The Royal Botanic Gardens, Kew\Desktop\Eremophila NMR"

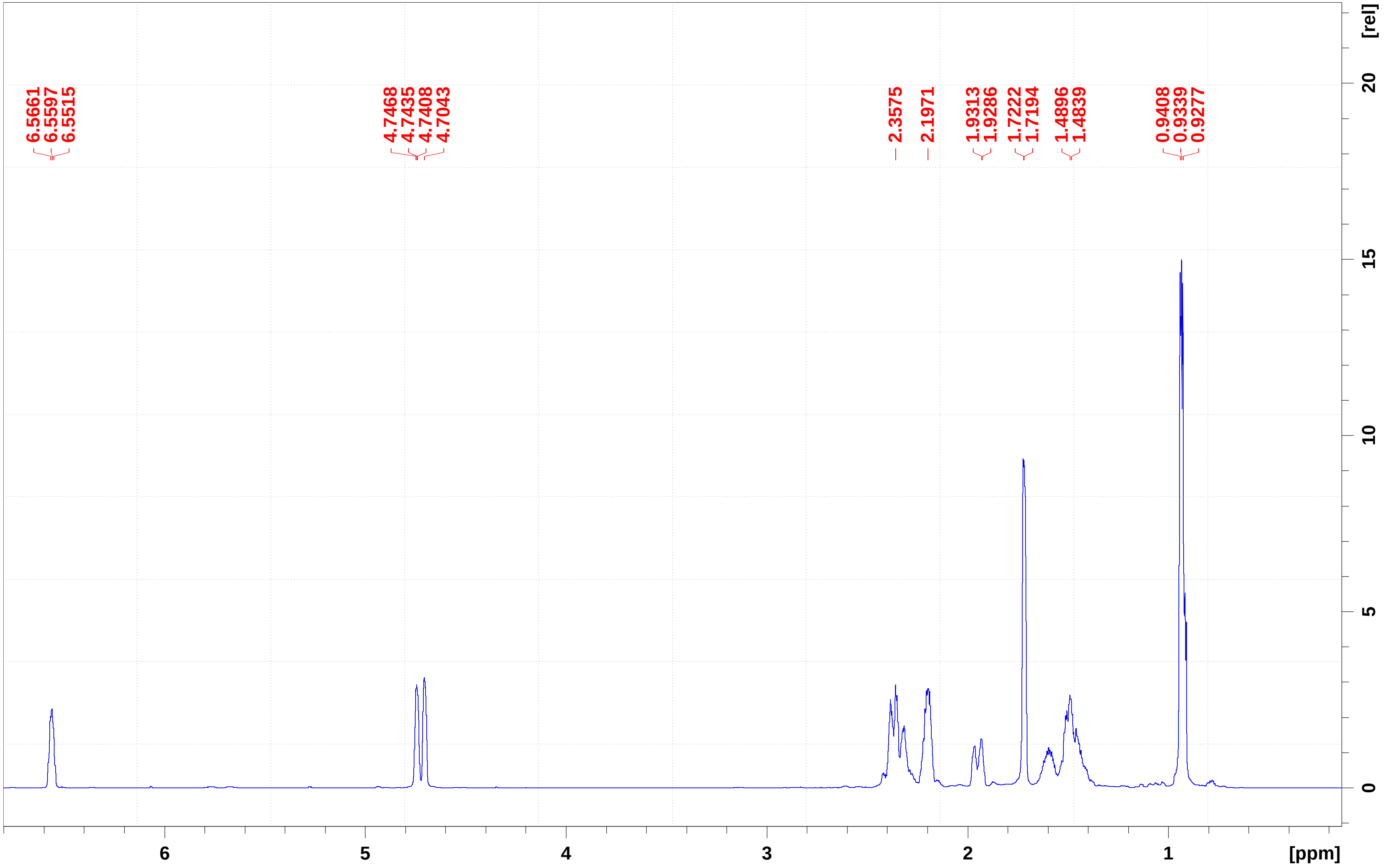

Eremophilone 11 1 "C:\Users\nmrsu\OneDrive - The Royal Botanic Gardens, Kew\Desktop\Eremophila NMR"

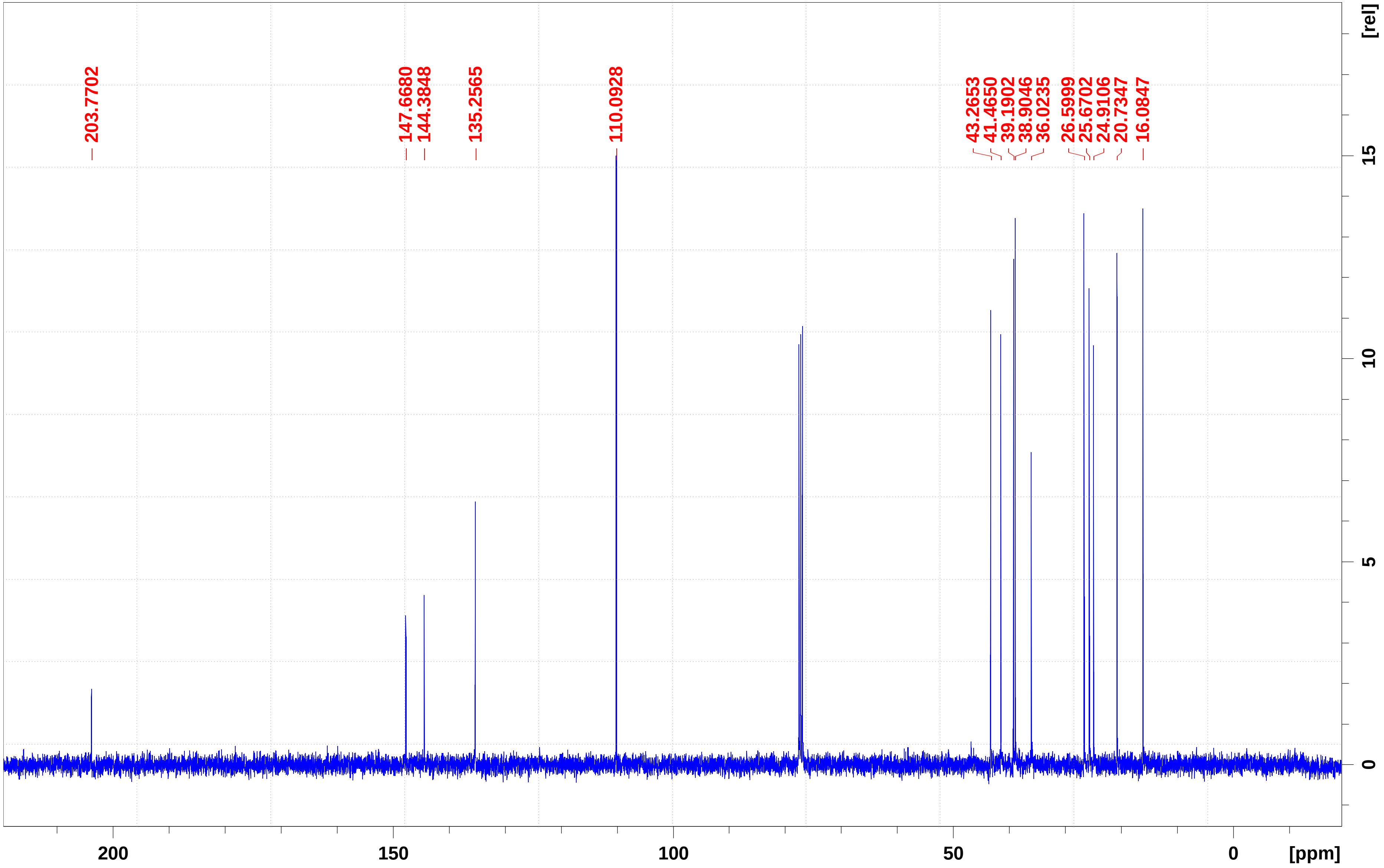

Freelingnite 1 1 "C:\Users\nmrsu\OneDrive - The Royal Botanic Gardens, Kew\Desktop\Eremophila NMR"

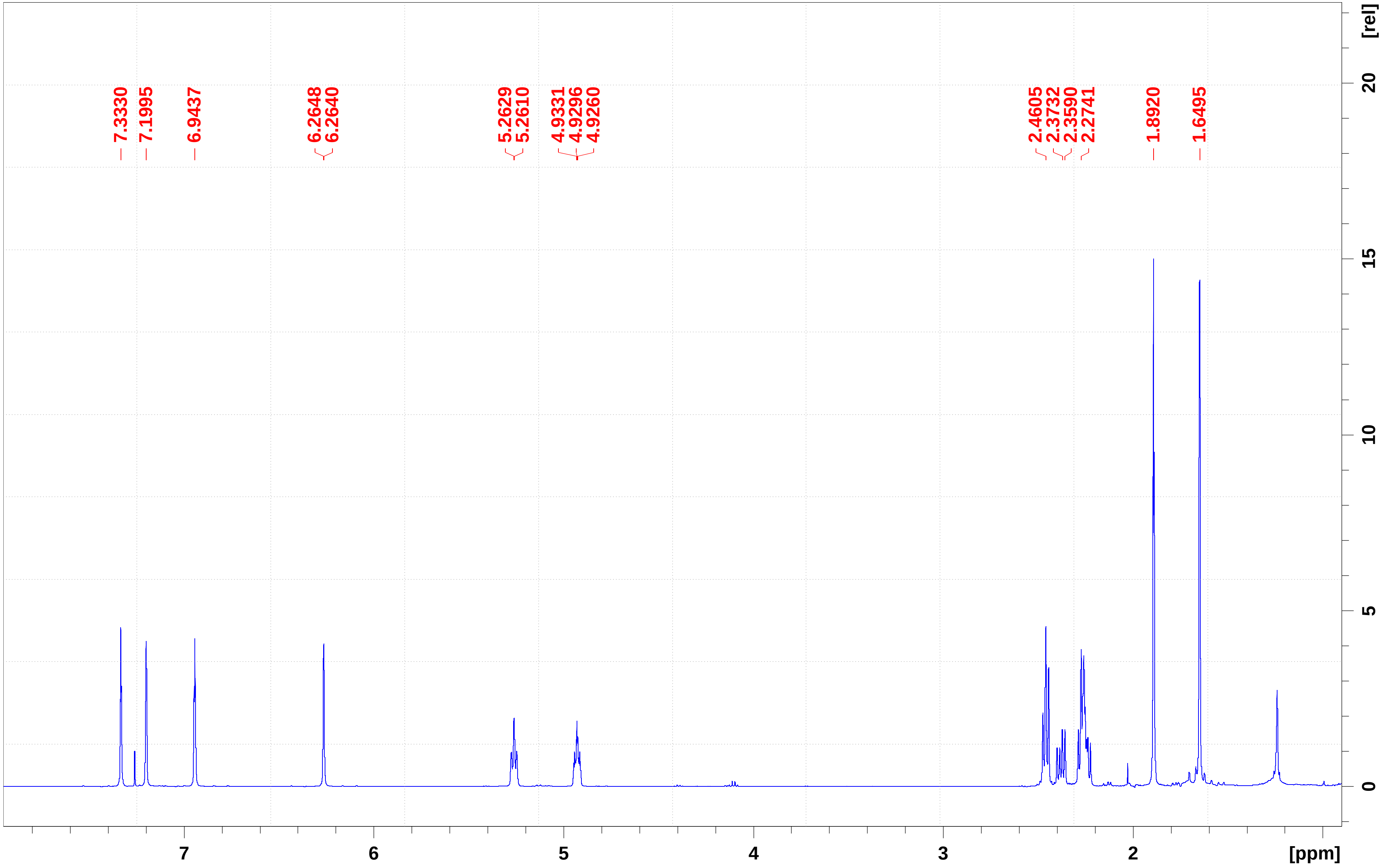

Freelingnite 2 1 "C:\Users\nmrsu\OneDrive - The Royal Botanic Gardens, Kew\Desktop\Eremophila NMR"

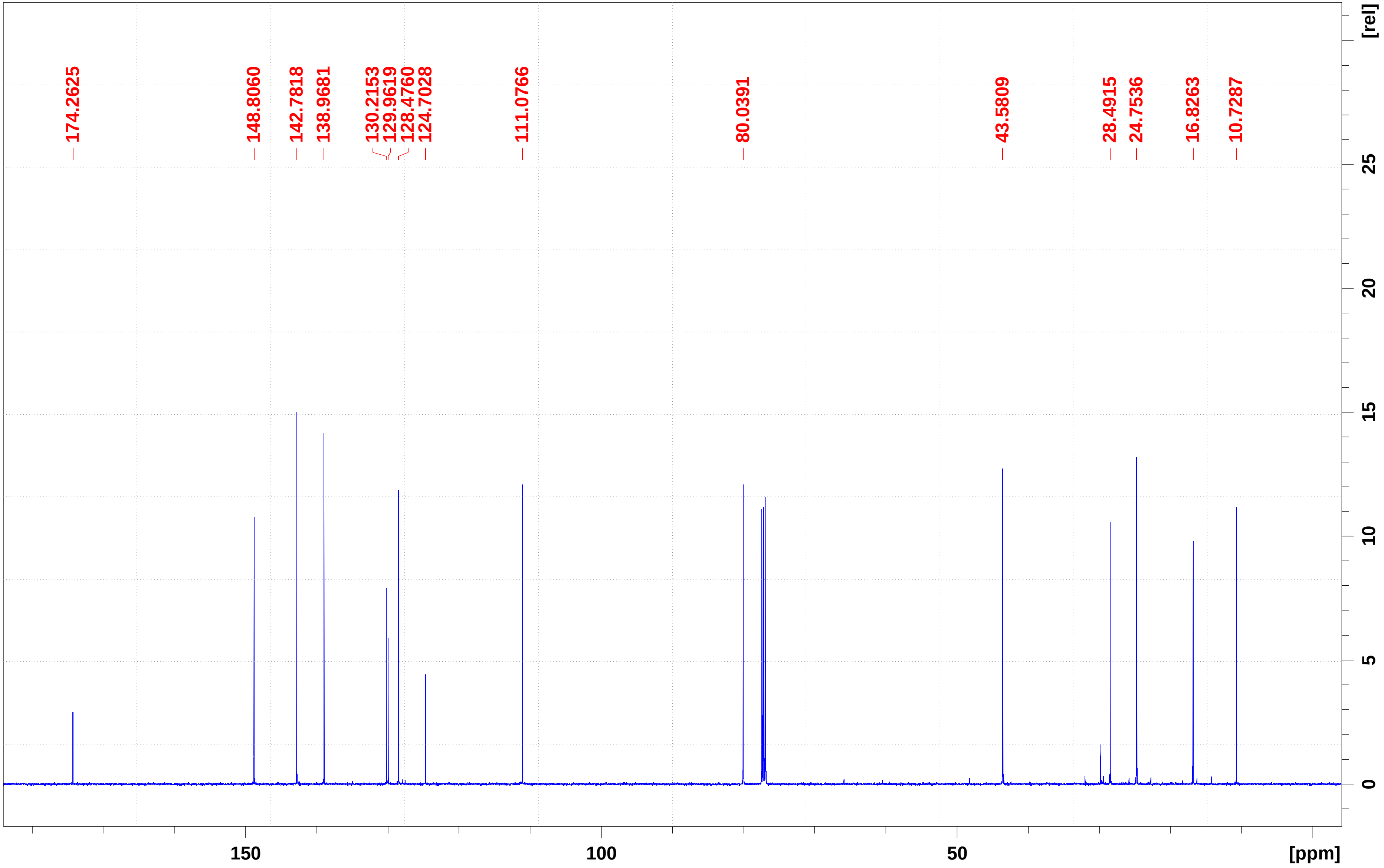

Hydroxyngaione 1 1 "C:\Users\nmrsu\OneDrive - The Royal Botanic Gardens, Kew\Desktop\Eremophila NMR"

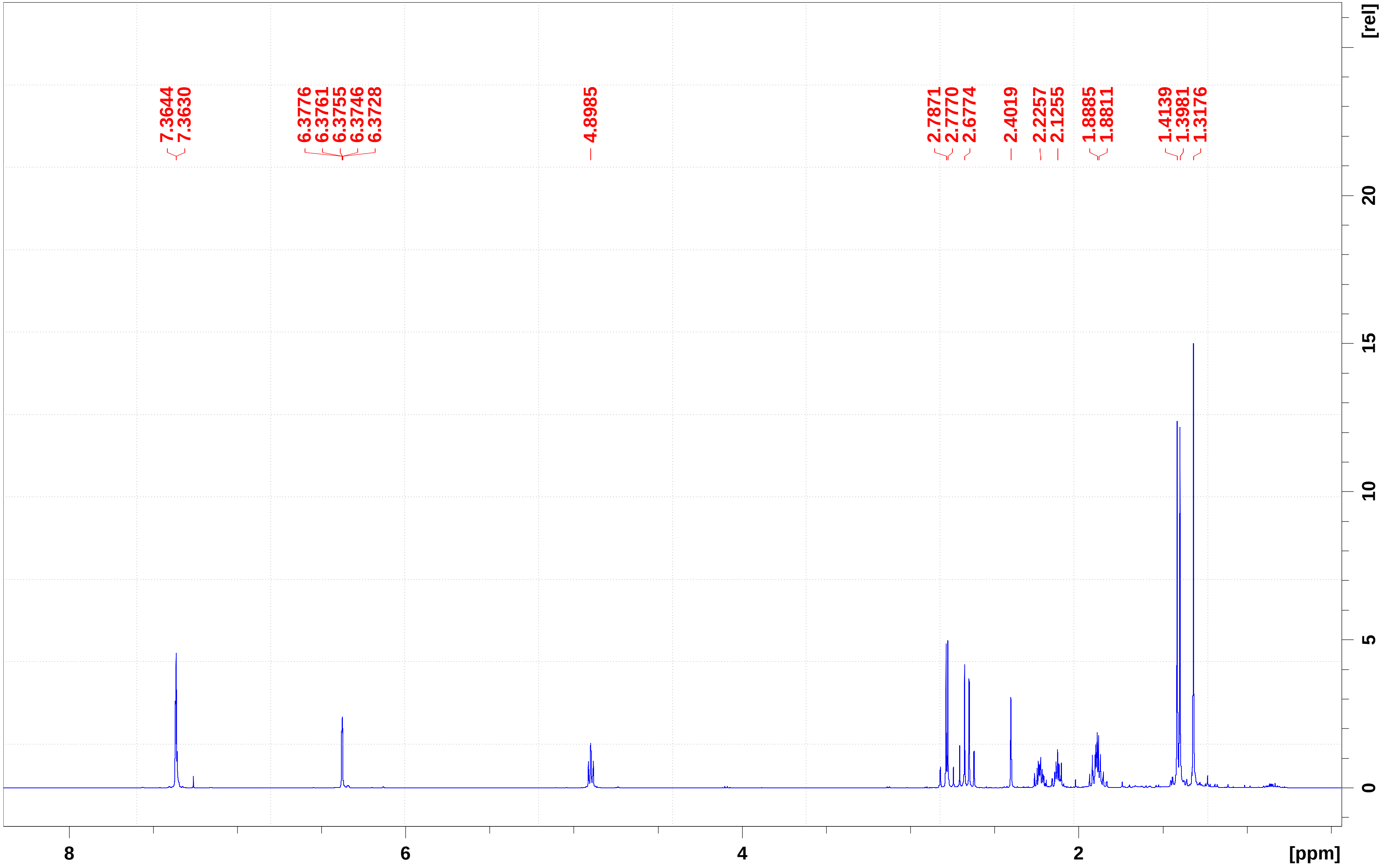

Hydroxyngaione 2 1 "C:\Users\nmrsu\OneDrive - The Royal Botanic Gardens, Kew\Desktop\Eremophila NMR"

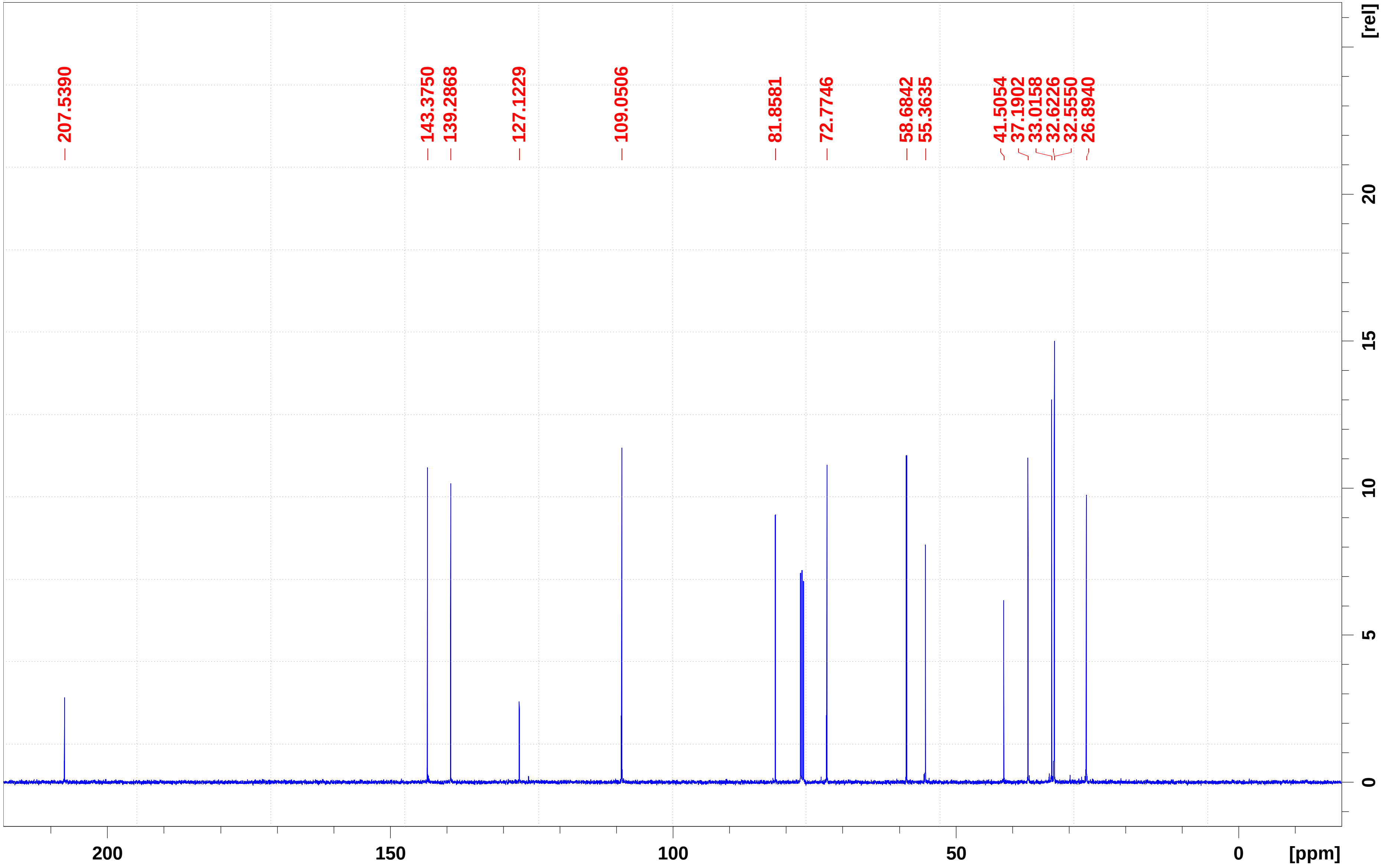

Methoxymyodesert-3-ene 1 1 "C:\Users\nmrsu\OneDrive - The Royal Botanic Gardens, Kew\Desktop\Eremophila NMR"

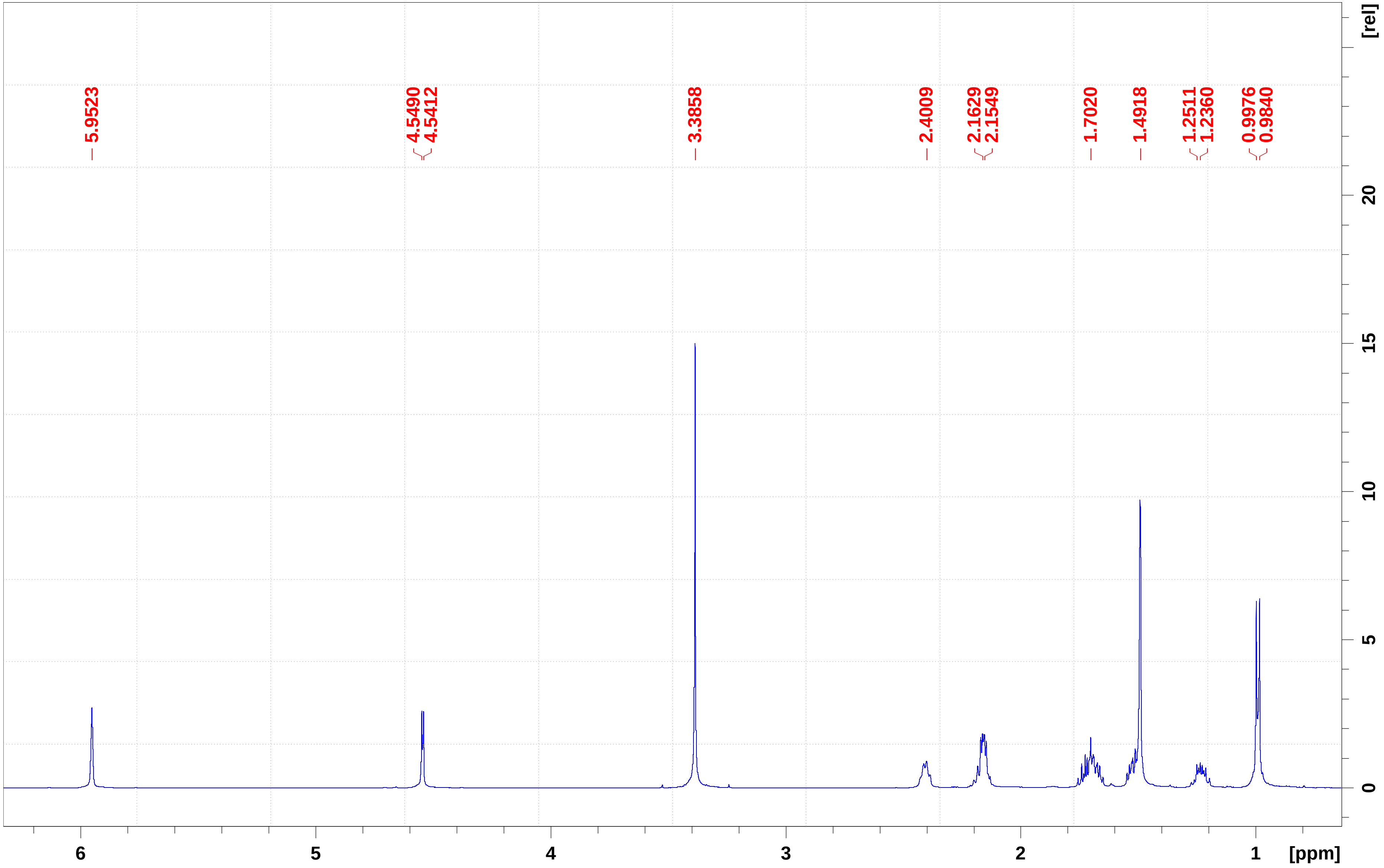

Methoxymyodesert-3-ene 2 1 "C:\Users\nmrsu\OneDrive - The Royal Botanic Gardens, Kew\Desktop\Eremophila NMR"

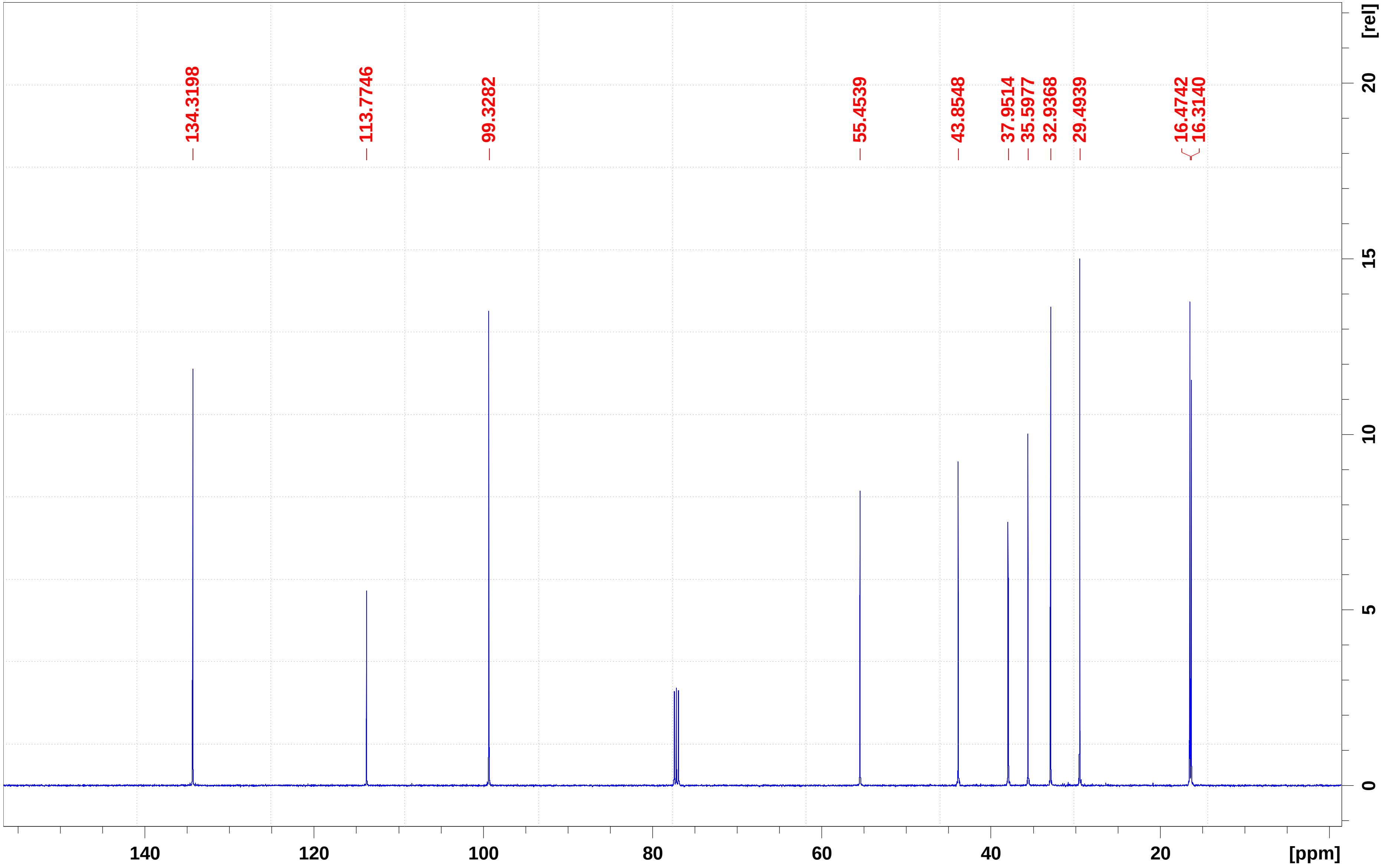

"Mitchellene B" 1 1 "C:\Users\nmrsu\OneDrive - The Royal Botanic Gardens, Kew\Desktop\Eremophila NMR"

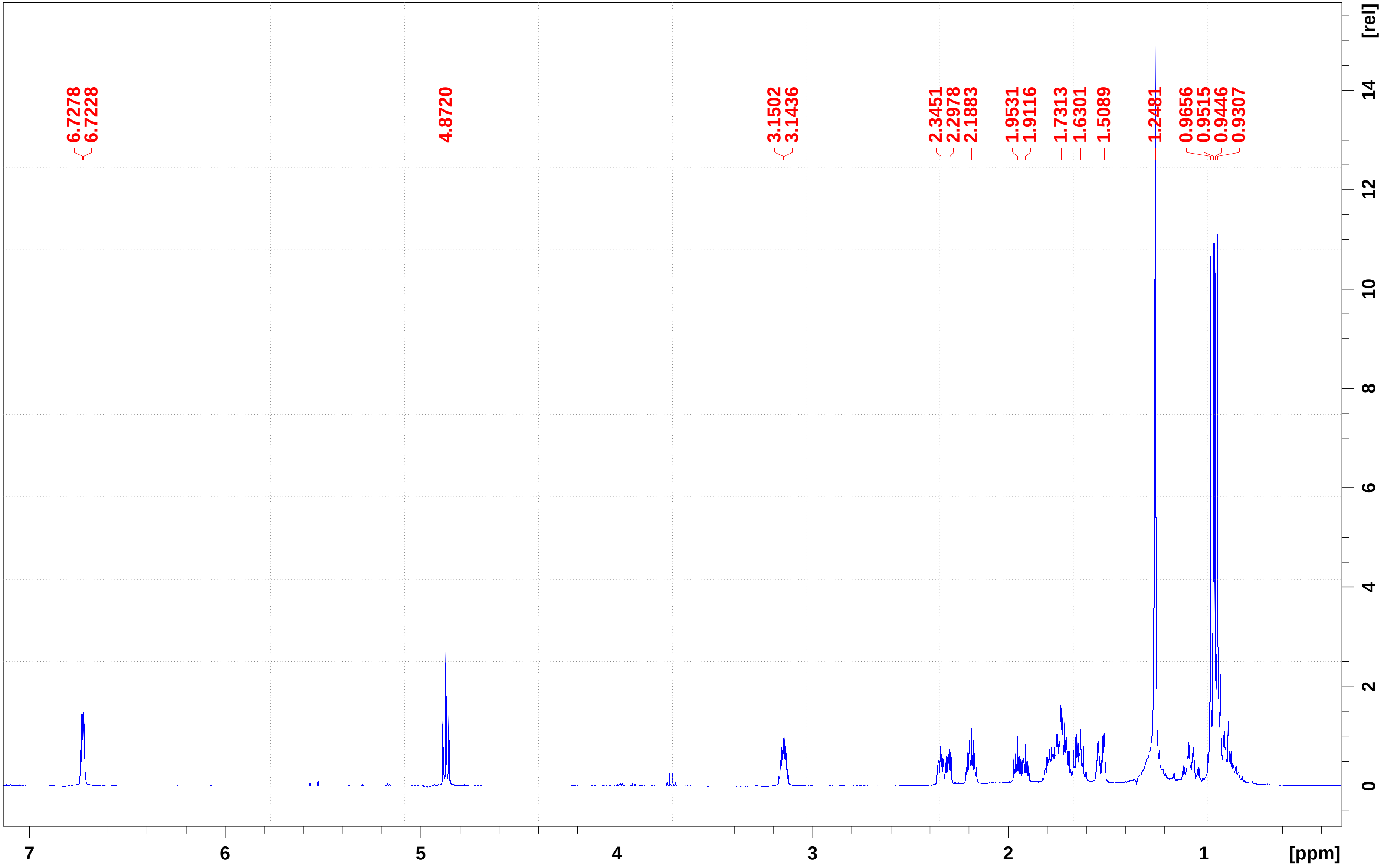

"Mitchellene B" 2 1 "C:\Users\nmrsu\OneDrive - The Royal Botanic Gardens, Kew\Desktop\Eremophila NMR"

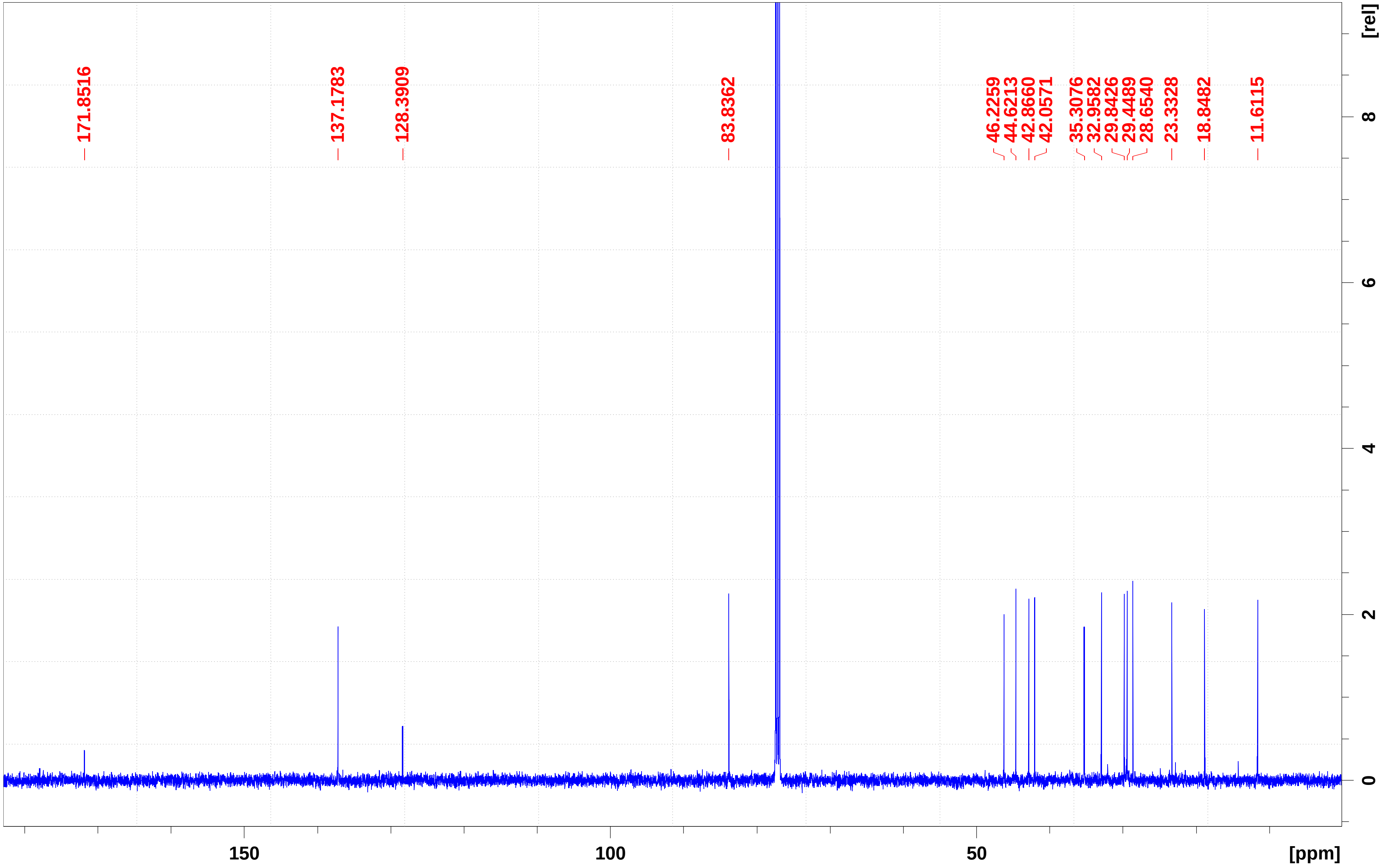

"Mitchellene F in CDCl3" 1 1 "C:\Users\nmrsu\OneDrive - The Royal Botanic Gardens, Kew\Desktop\Eremophila NMR\Mitchellene F"

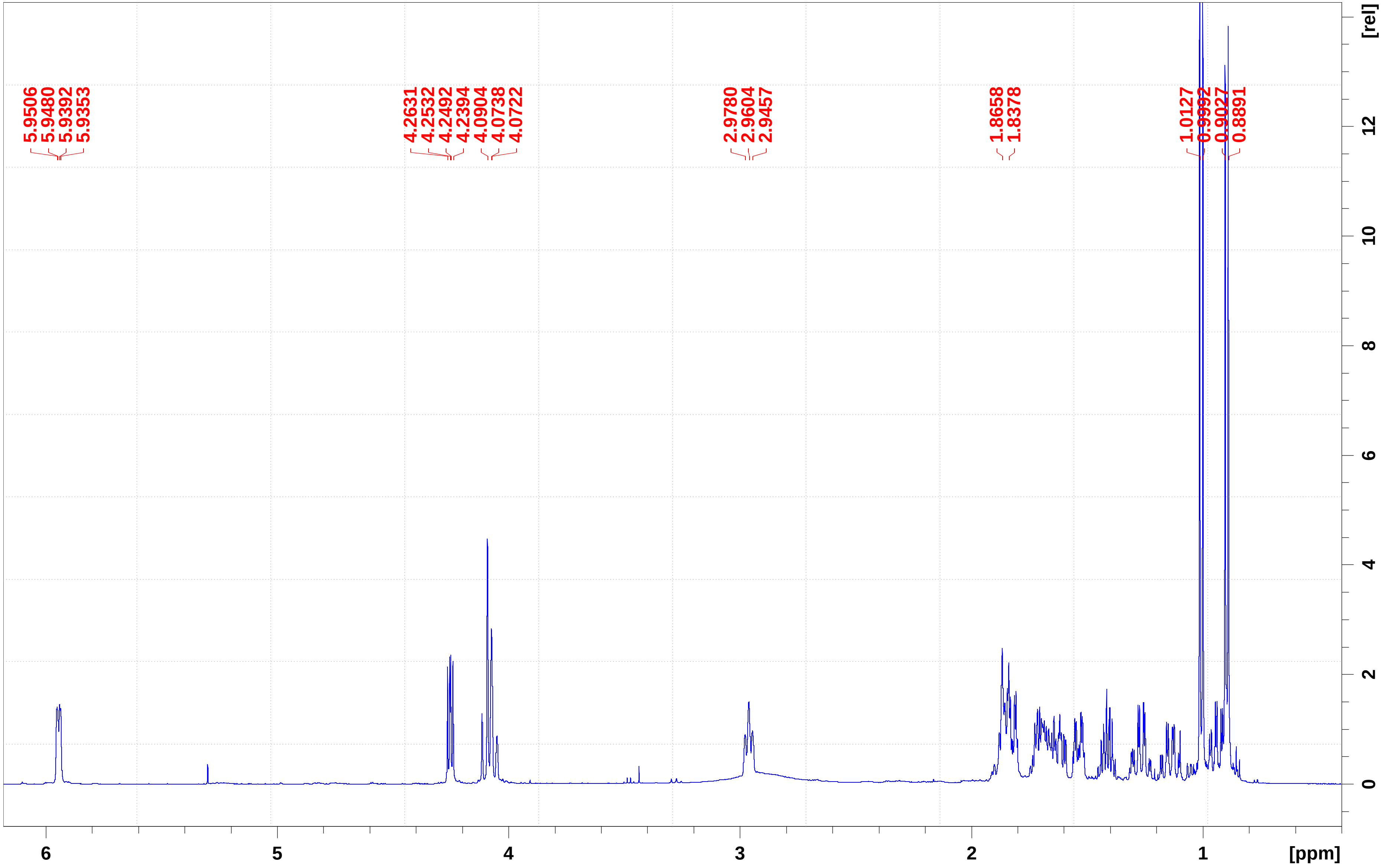

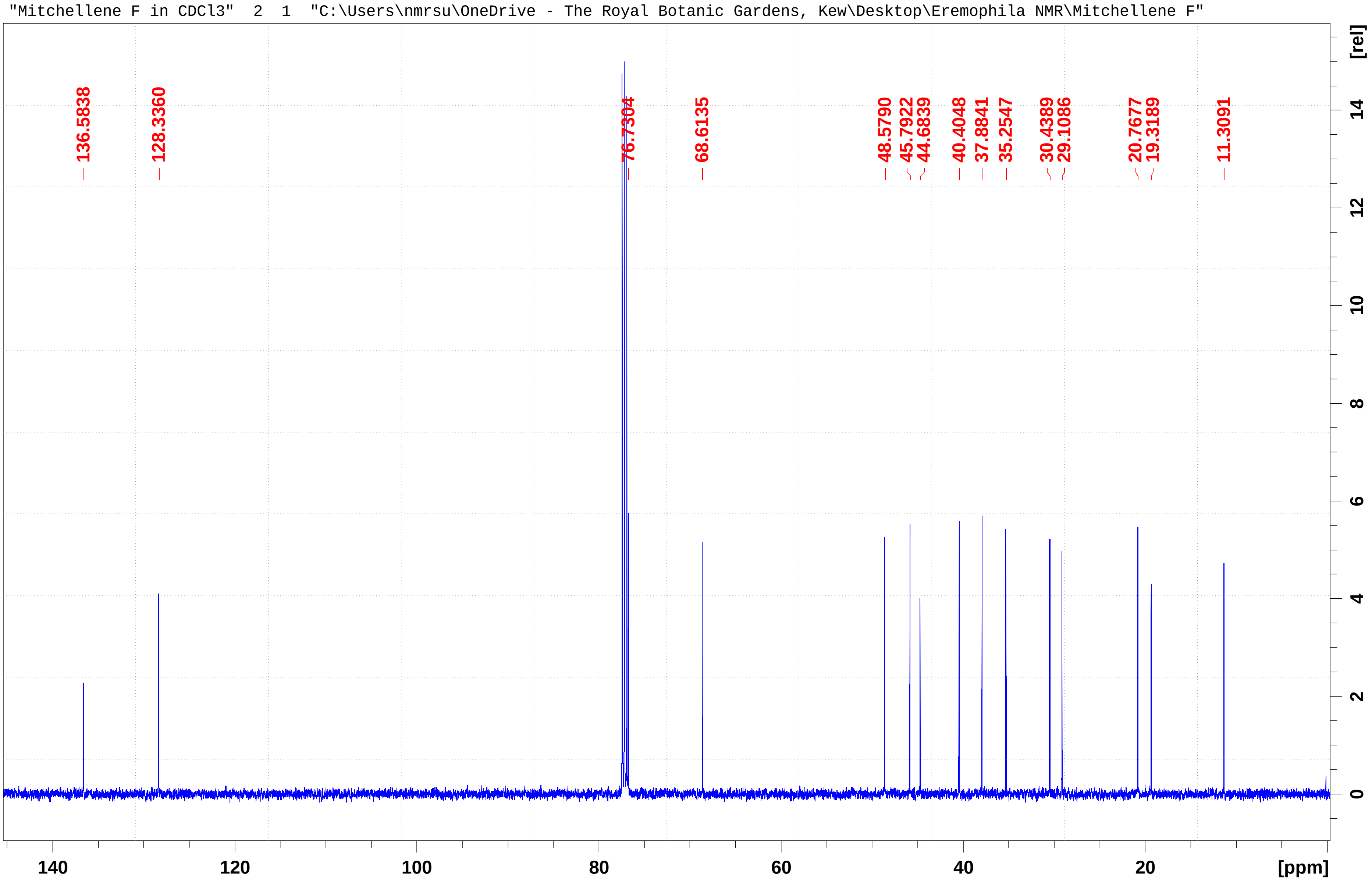
"Mitchellene F in CDCl3" 2 1 "C:\Users\nmrsu\OneDrive - The Royal Botanic Gardens, Kew\Desktop\Eremophila NMR\Mitchellene F"
136.5838
128.3360
76.7304
68.6135
48.5790
45.7922
44.6839
40.4048
37.8841
35.2547
30.4389
29.1086
20.7677
19.3189
11.3091
[rel]
14
12
10
8
6
4
2
0
140
120
100
80
60
40
20
[ppm]

"Mitchellene G" 1 1 "C:\Users\nmrsu\OneDrive - The Royal Botanic Gardens, Kew\Desktop\Eremophila NMR"

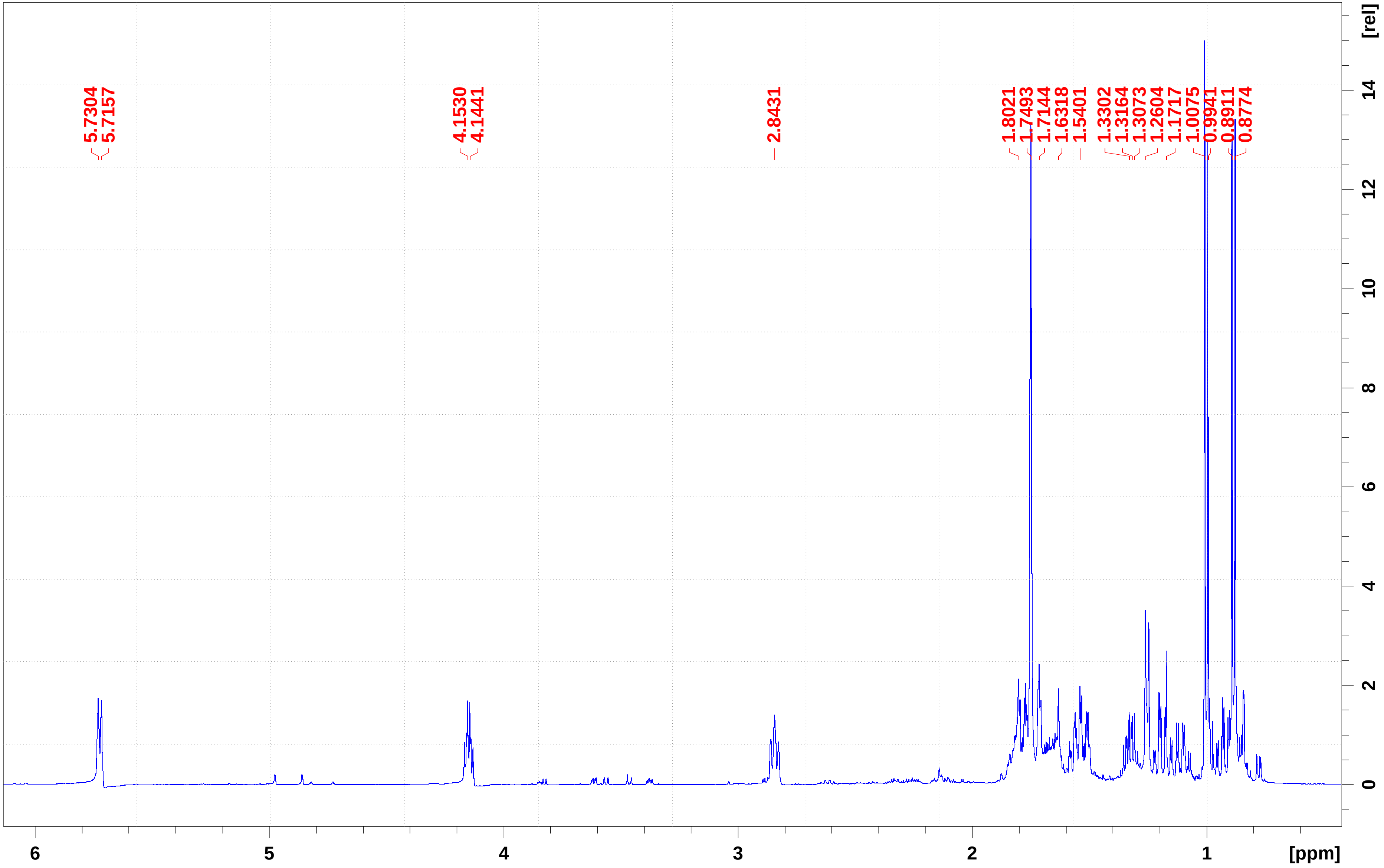

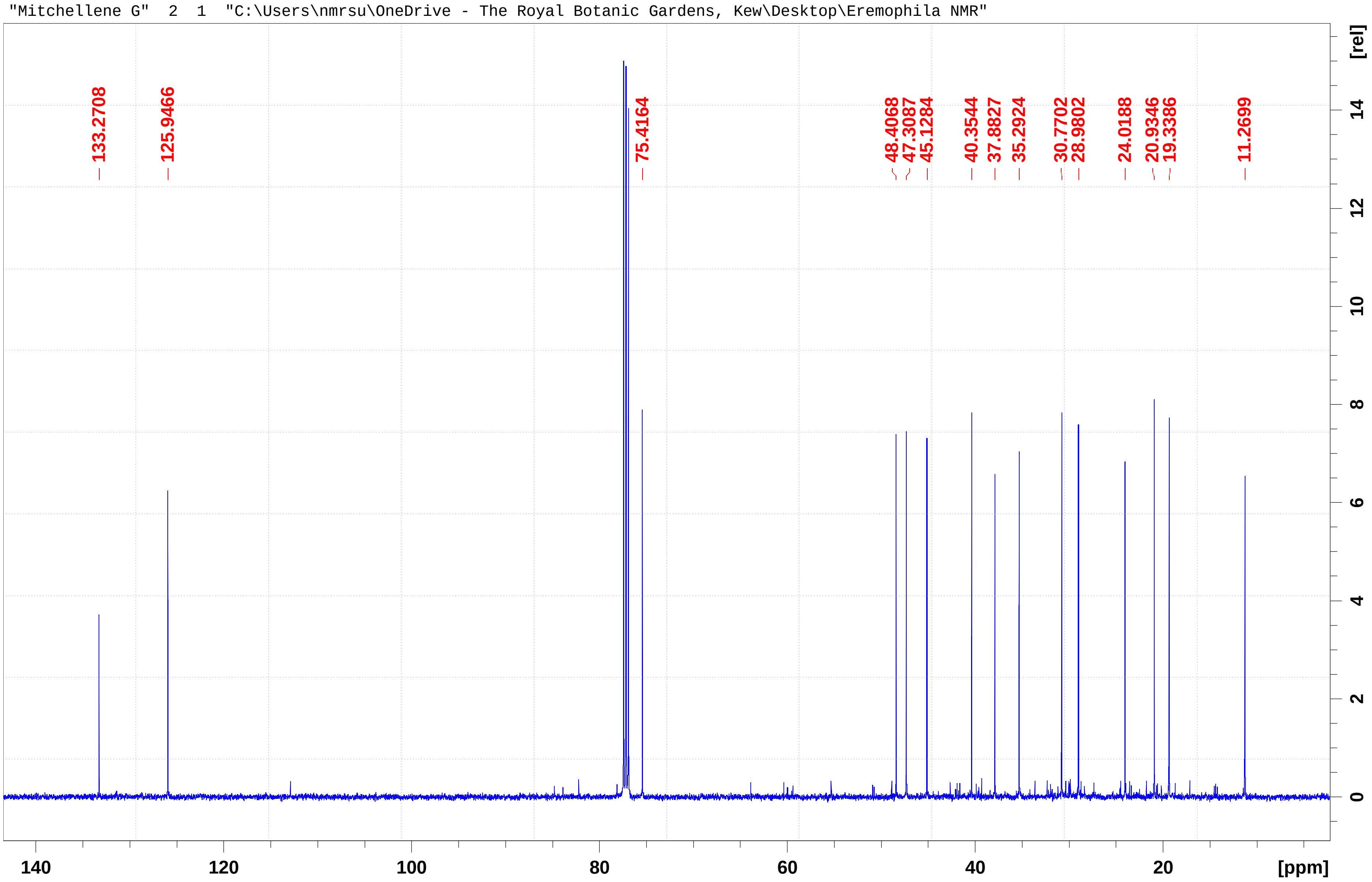
"Mitchellene G" 2 1 "C:\Users\nmrsu\OneDrive - The Royal Botanic Gardens, Kew\Desktop\Eremophila NMR"
133.2708
125.9466
75.4164
48.4068
47.3087
45.1284
40.3544
37.8827
35.2924
30.7702
28.9802
24.0188
20.9346
19.3386
11.2699
[rel]
[ppm]

Myodesmone 1 1 "C:\Users\nmrsu\OneDrive - The Royal Botanic Gardens, Kew\Desktop\Eremophila NMR"

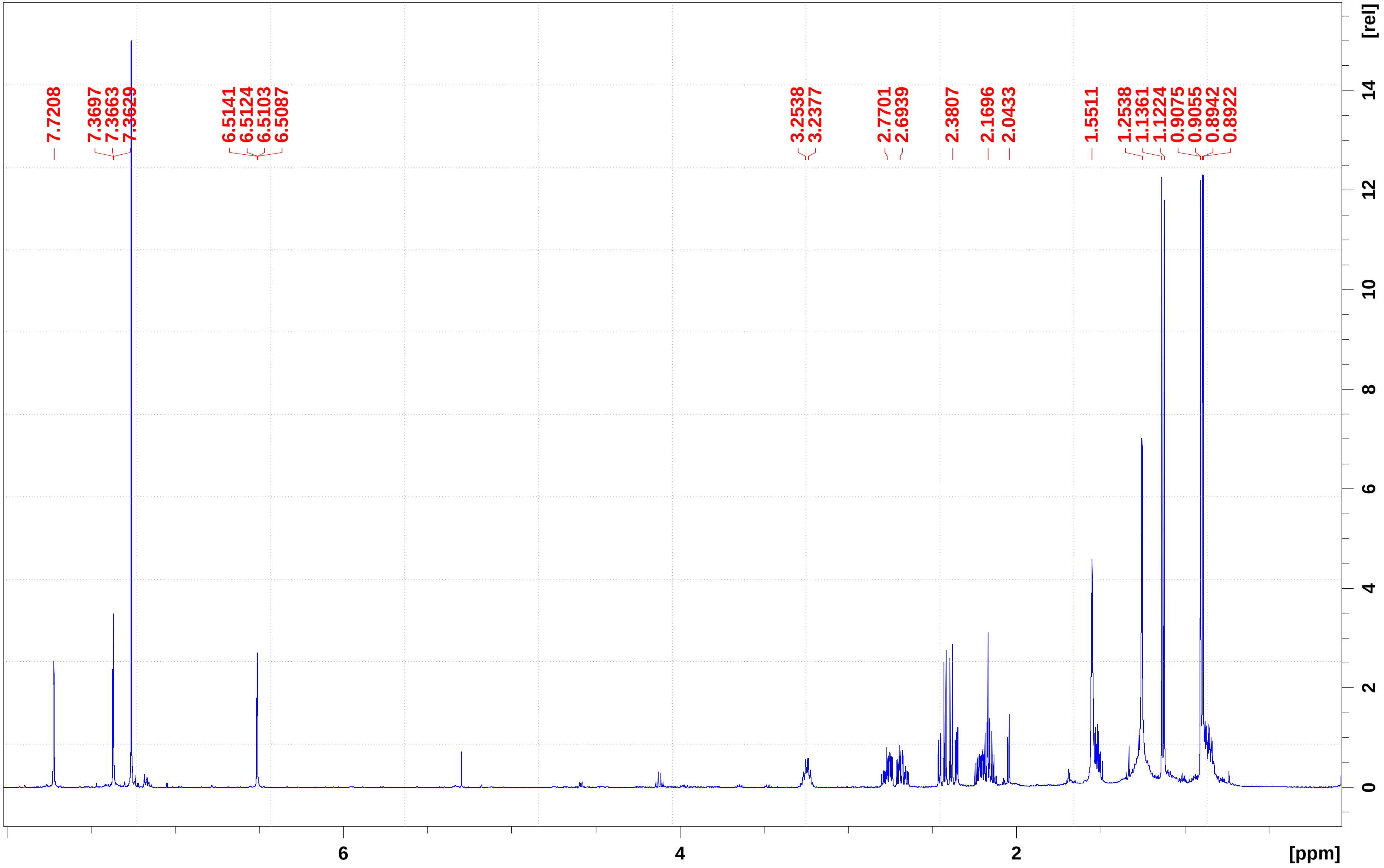

Myodesmone 3 1 "C:\Users\nmrsu\OneDrive - The Royal Botanic Gardens, Kew\Desktop\Eremophila NMR"

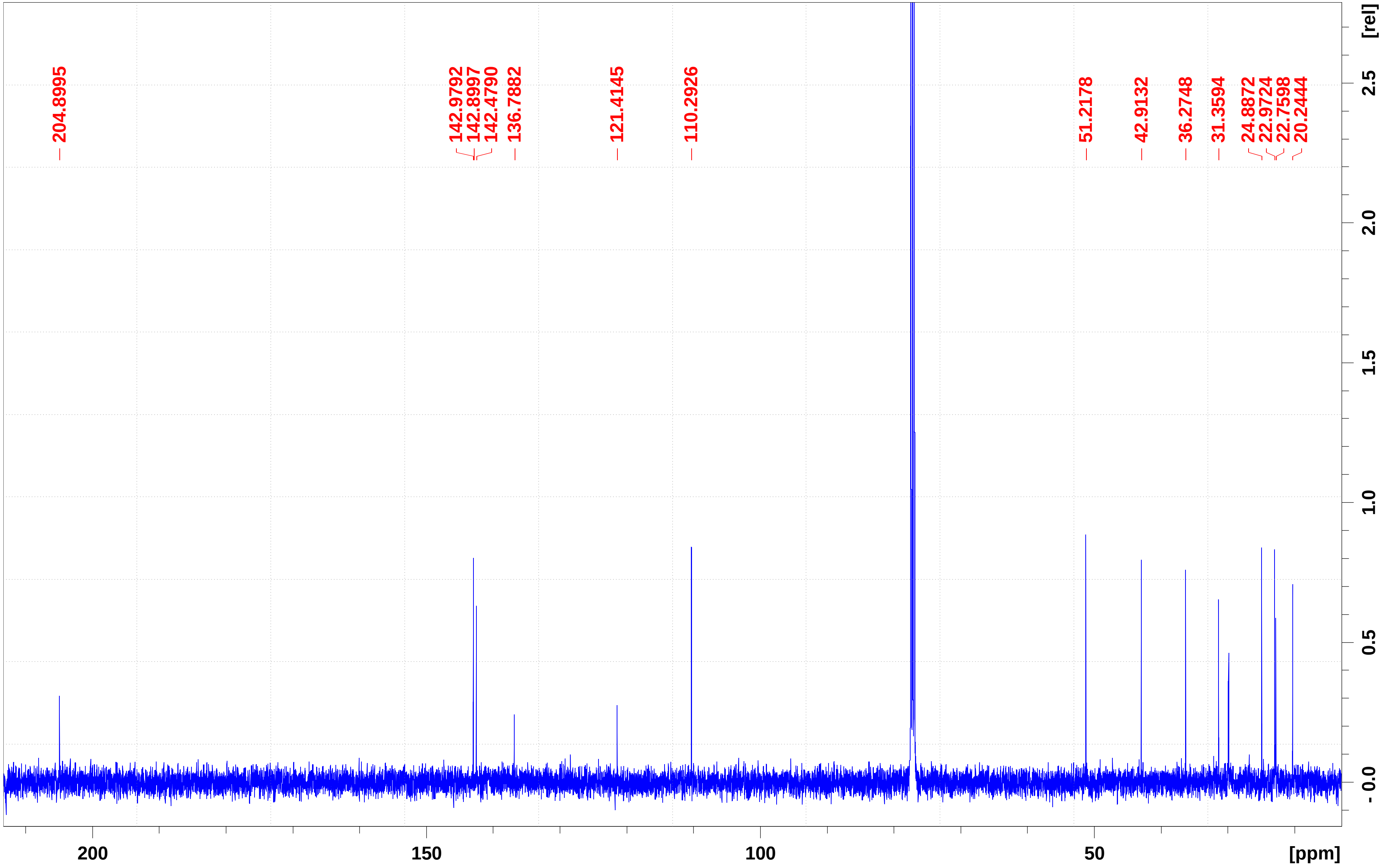

Myomontanone 1 1 "C:\Users\nmrsu\OneDrive - The Royal Botanic Gardens, Kew\Desktop\Eremophila NMR"

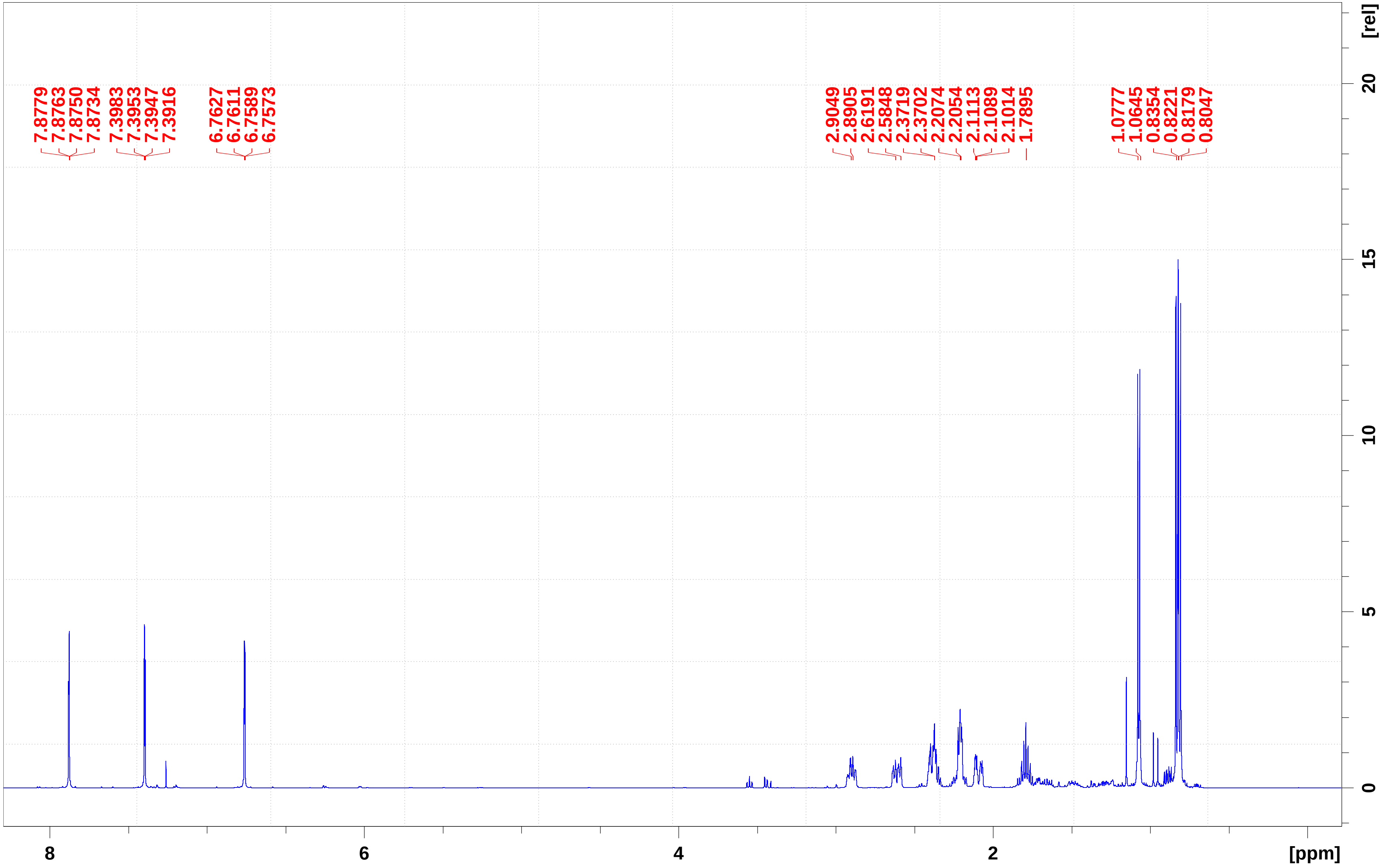

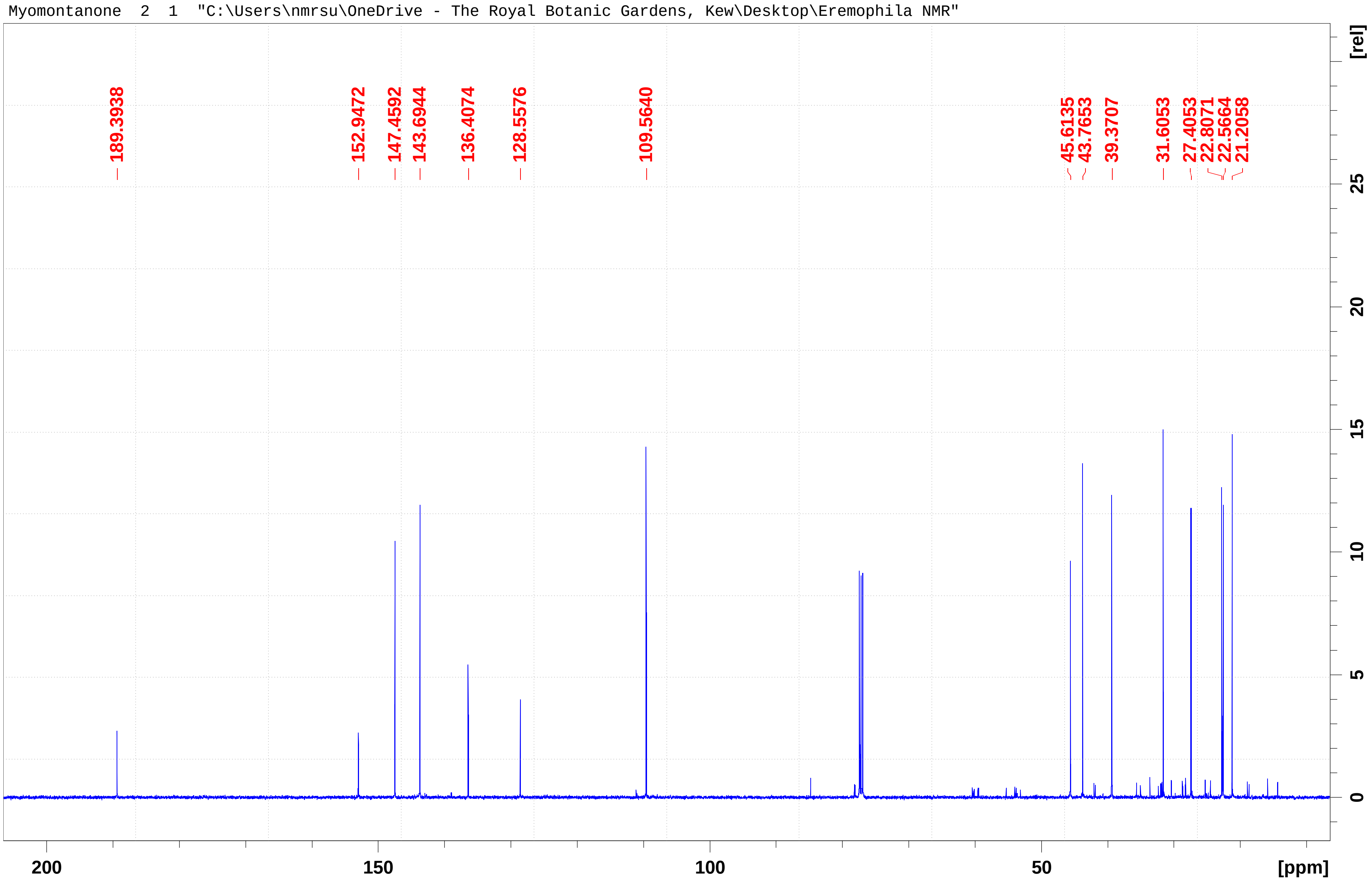

Myomontanone 2 1 "C:\Users\nmrsu\OneDrive - The Royal Botanic Gardens, Kew\Desktop\Eremophila NMR"
189.3938
152.9472
147.4592
143.6944
136.4074
128.5576
109.5640
45.6135
43.7653
39.3707
31.6053
27.4053
22.8071
22.5664
21.2058
[rel]
25
20
15
10
5
0
200
150
100
50
[ppm]

Myoporone 1 1 "C:\Users\nmrsu\OneDrive - The Royal Botanic Gardens, Kew\Desktop\Eremophila NMR"

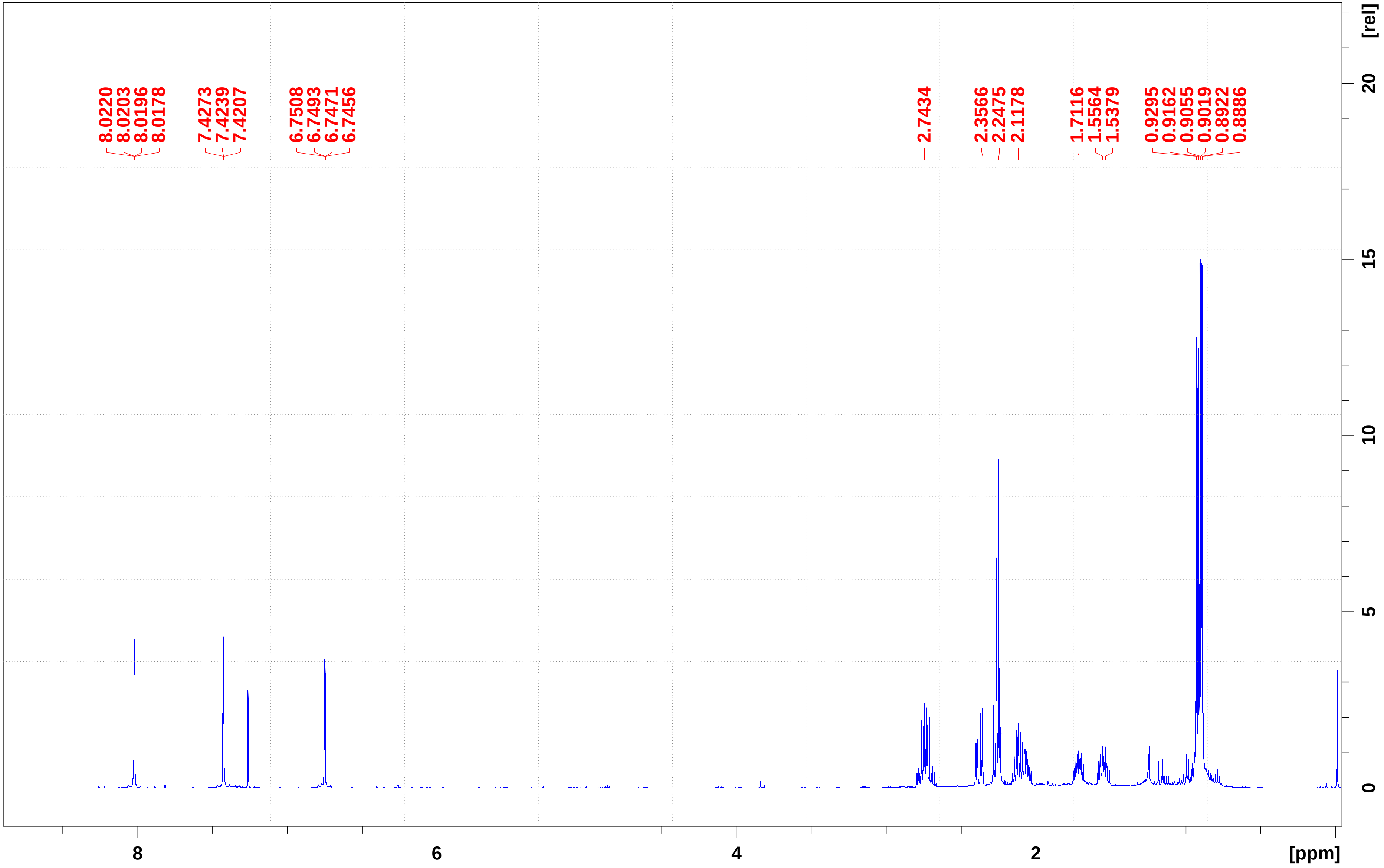

Myoporone 2 1 "C:\Users\nmrsu\OneDrive - The Royal Botanic Gardens, Kew\Desktop\Eremophila NMR"

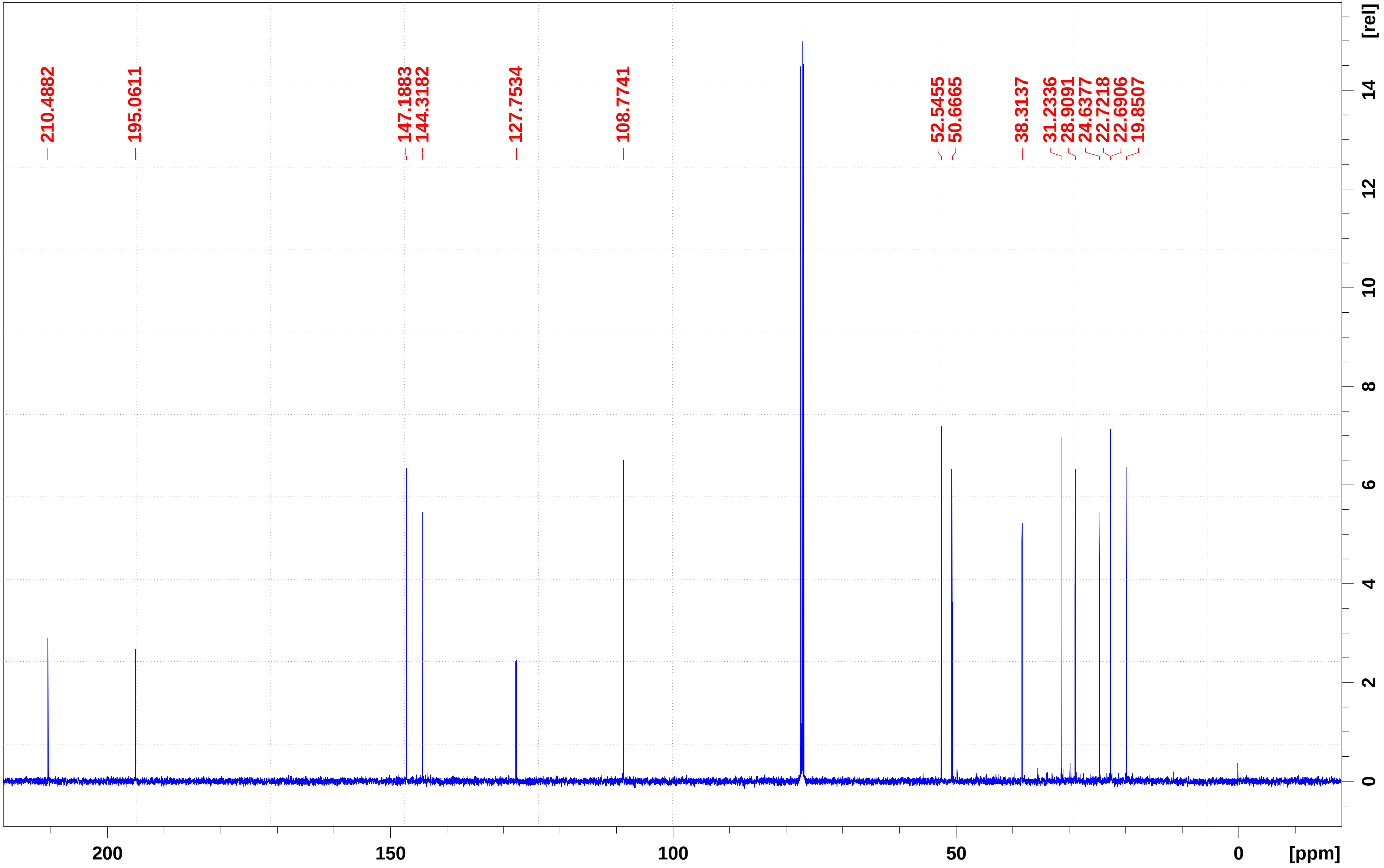

Ngaione 1 1 "C:\Users\nmrsu\OneDrive - The Royal Botanic Gardens, Kew\Desktop\Eremophila NMR"

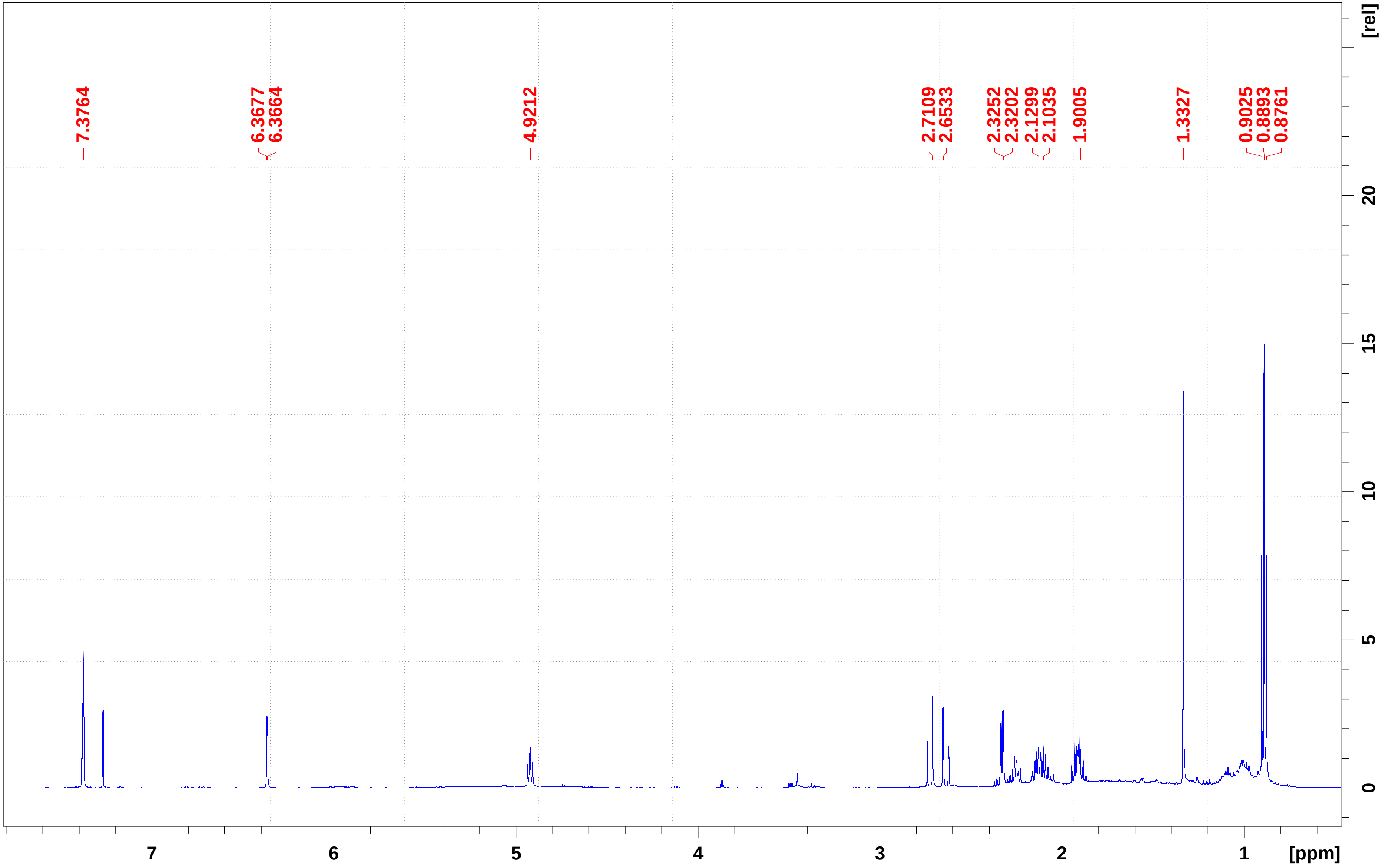

Ngaione 2 1 "C:\Users\nmrsu\OneDrive - The Royal Botanic Gardens, Kew\Desktop\Eremophila NMR"

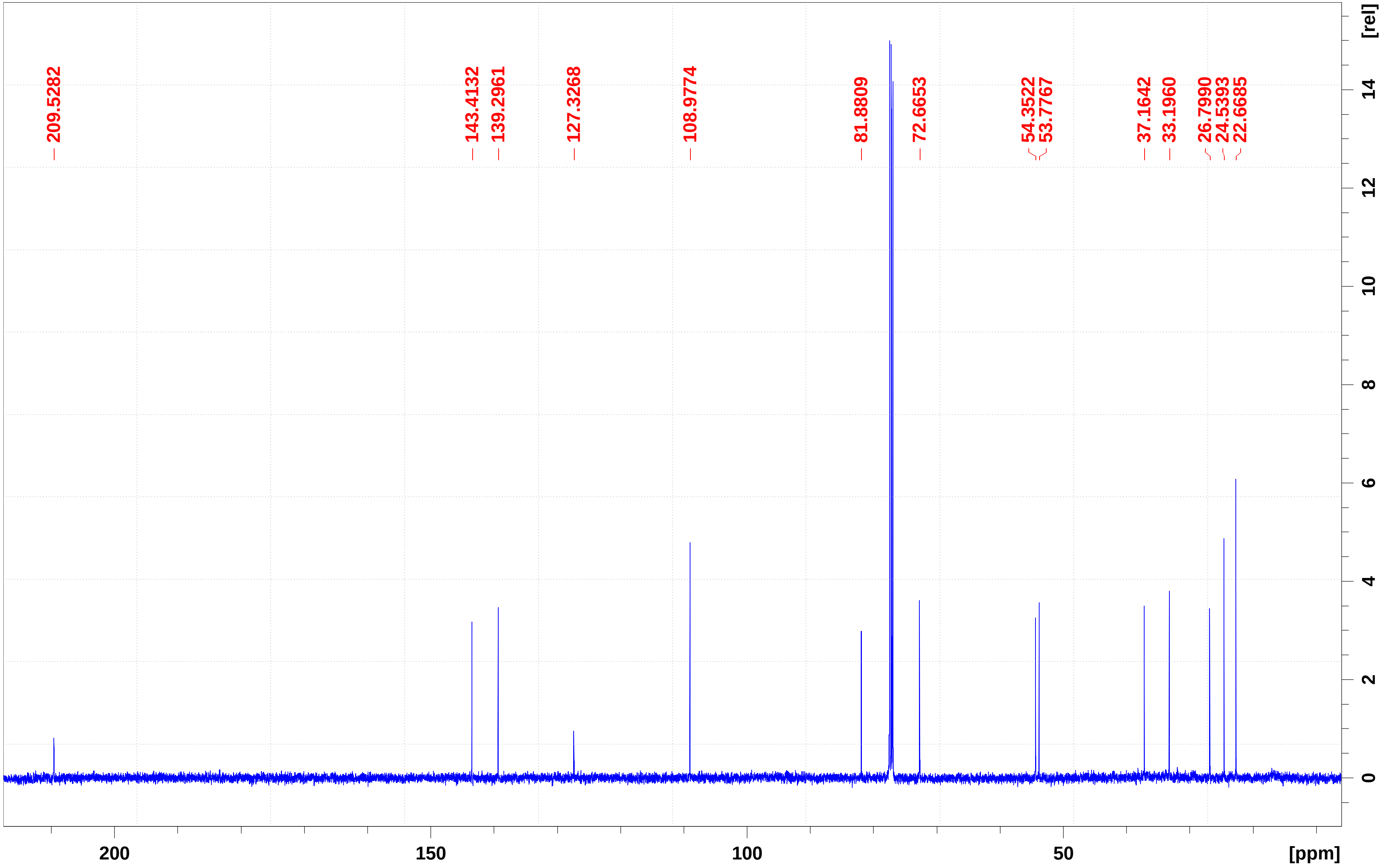

"Oppositifolic acid" 2 1 "C:\Users\nmrsu\OneDrive - The Royal Botanic Gardens, Kew\Desktop\Eremophila NMR"

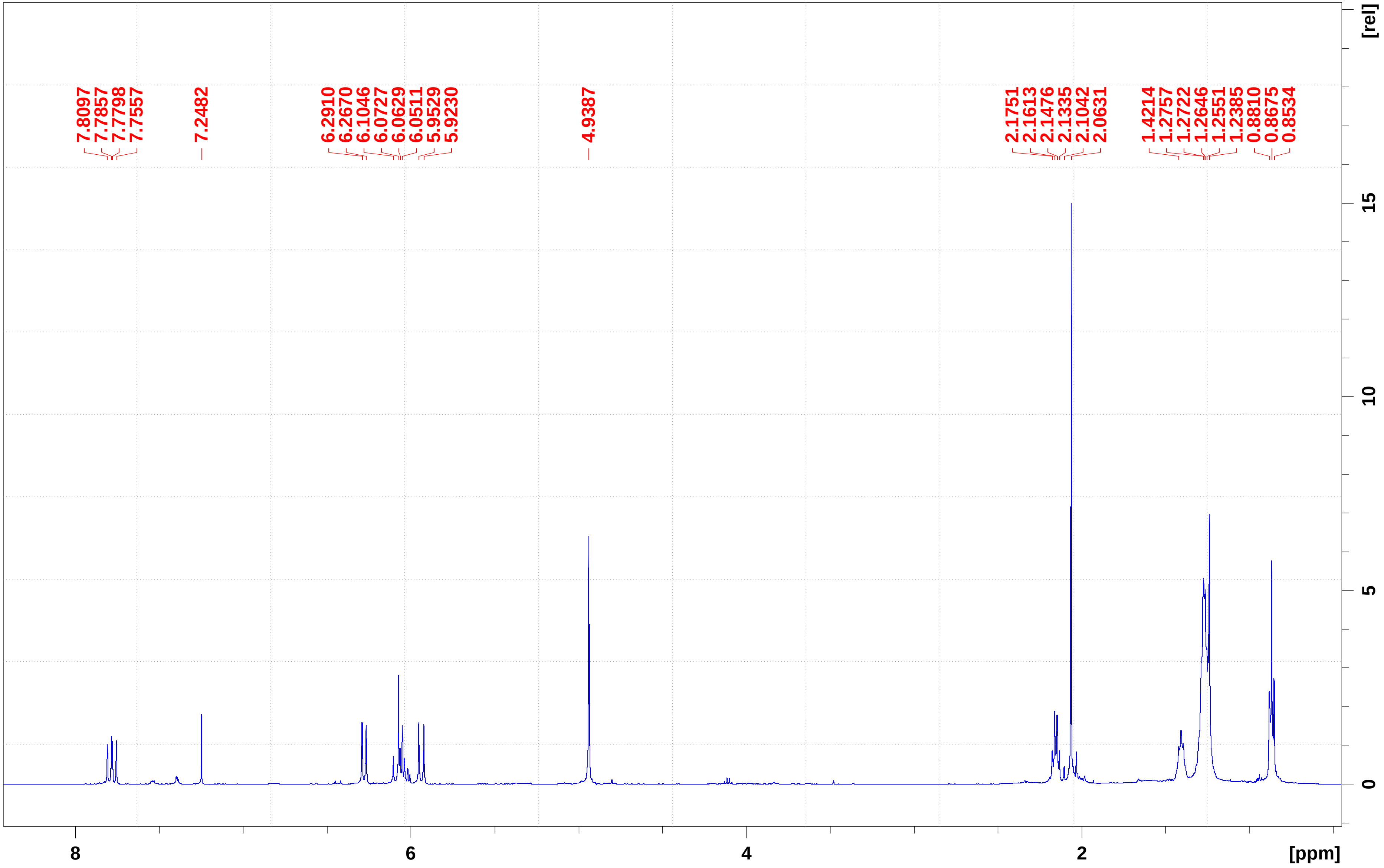

"Oppositifolic acid" 3 1 "C:\Users\nmrsu\OneDrive - The Royal Botanic Gardens, Kew\Desktop\Eremophila NMR"

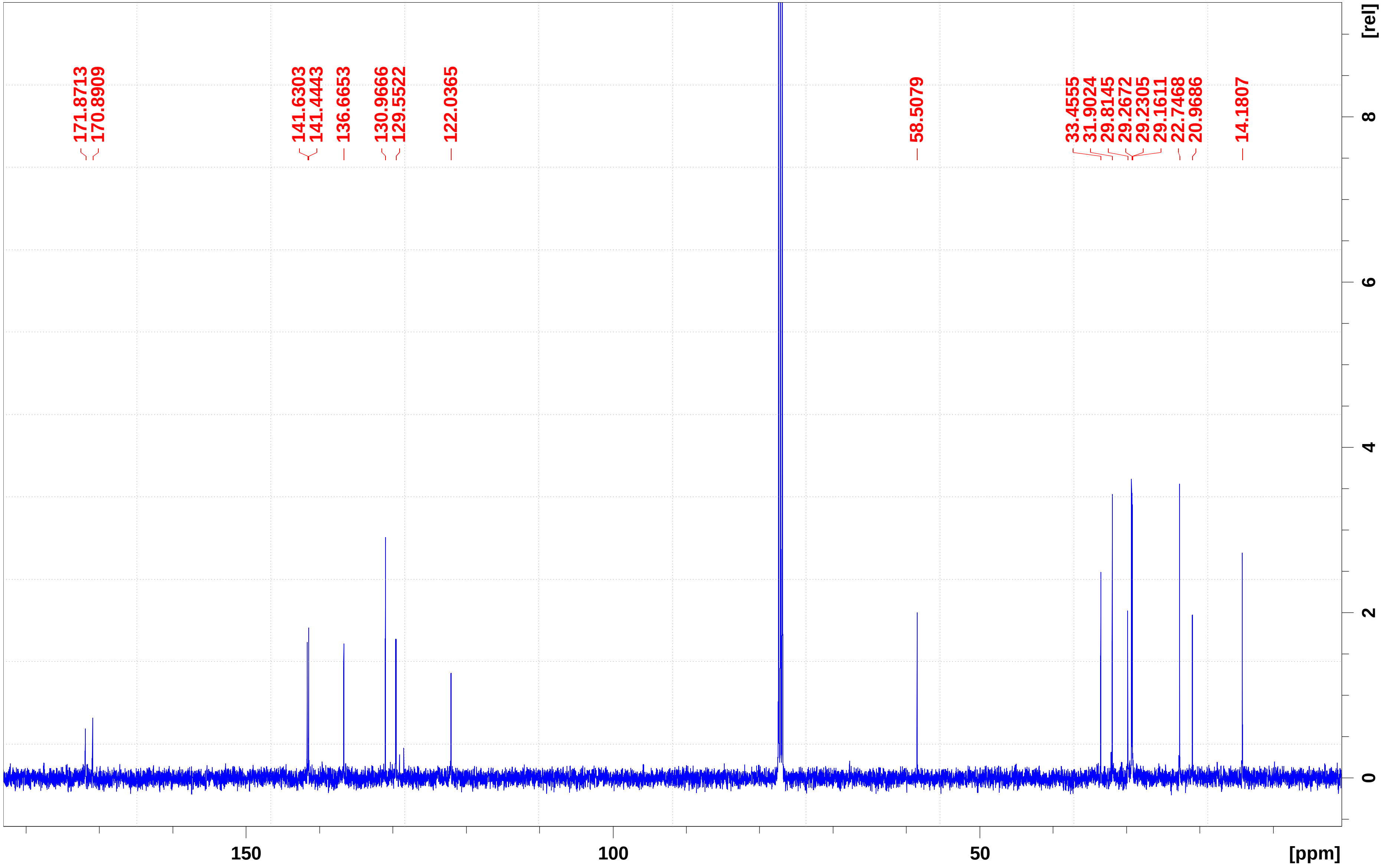

Santalcamphor 10 1 "C:\Users\nmrsu\OneDrive - The Royal Botanic Gardens, Kew\Desktop\Eremophila NMR"

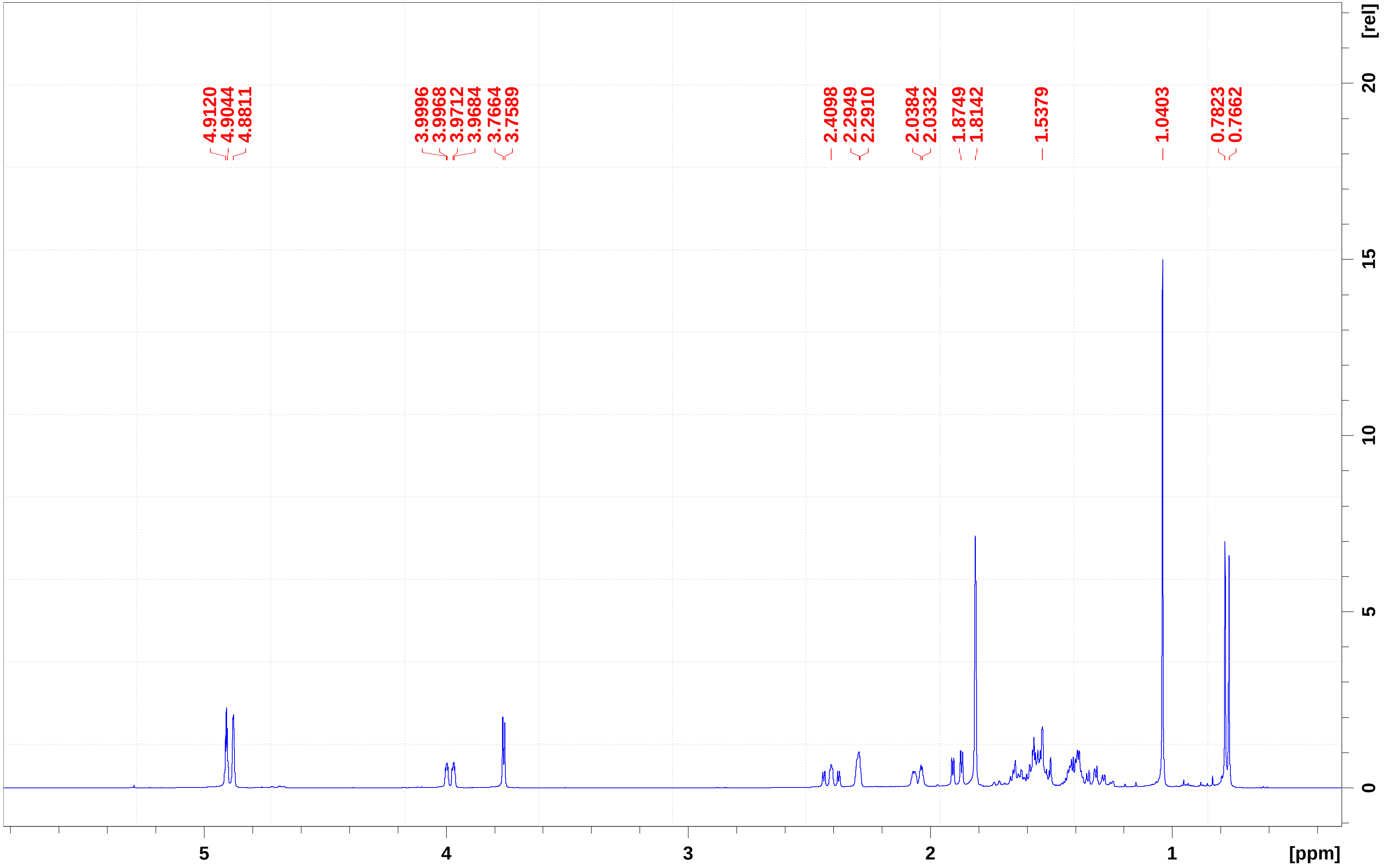

Santalcamphor 11 1 "C:\Users\nmrsu\OneDrive - The Royal Botanic Gardens, Kew\Desktop\Eremophila NMR"

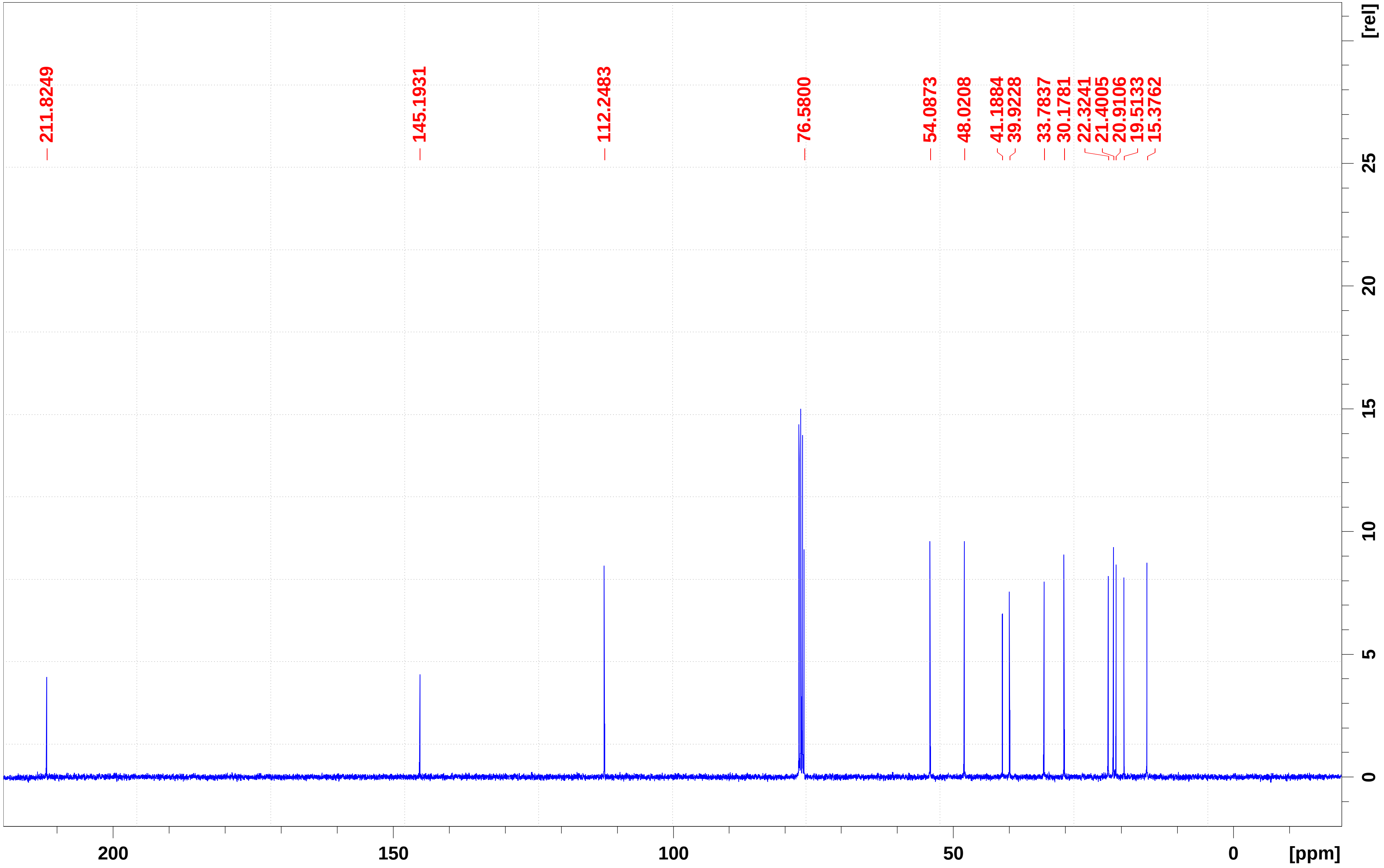

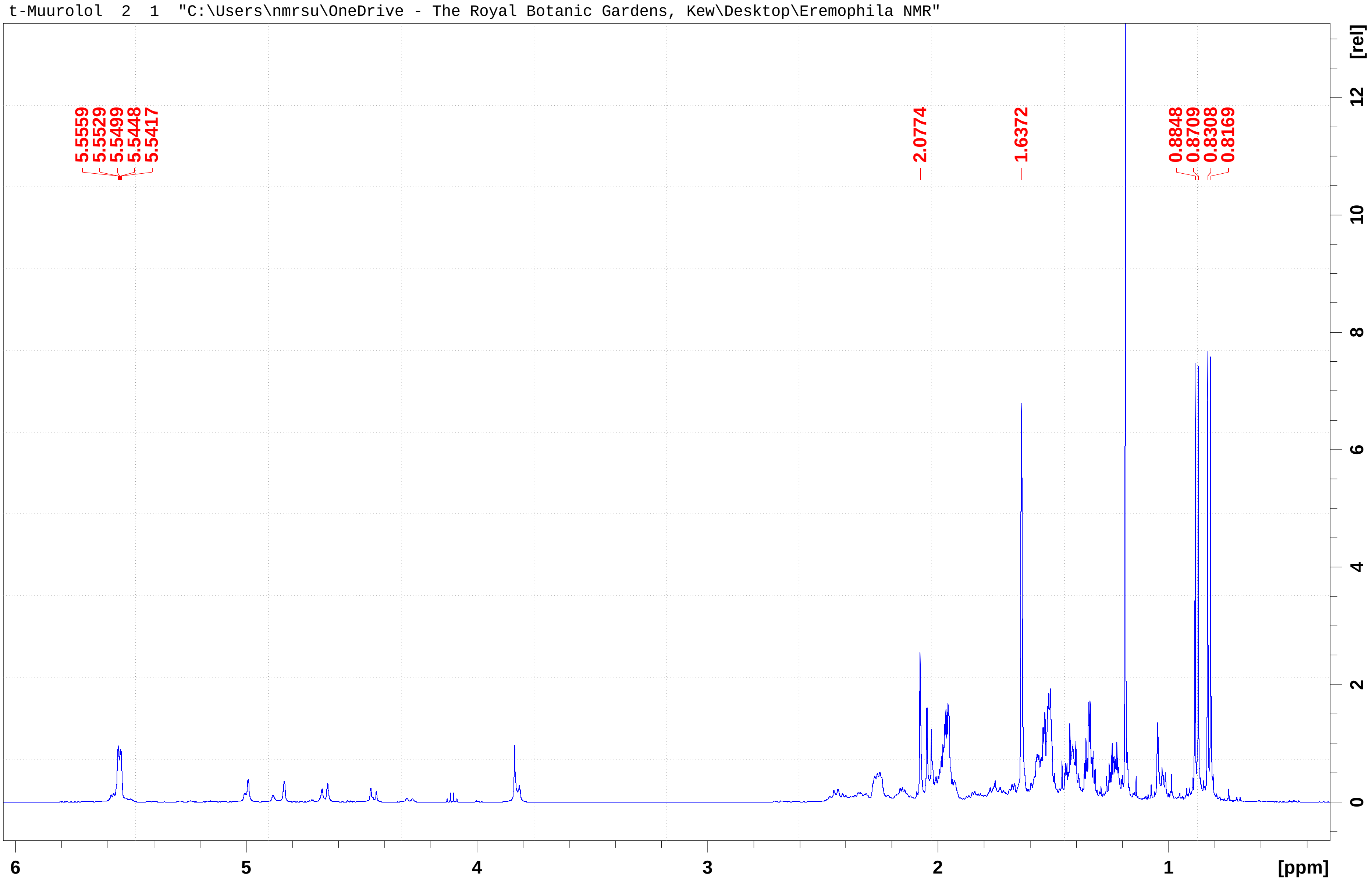
t-Muurolol 2 1 "C:\Users\nmrsu\OneDrive - The Royal Botanic Gardens, Kew\Desktop\Eremophila NMR"
5.5559
5.5529
5.5499
5.5448
5.5417
2.0774
1.6372
0.8848
0.8709
0.8308
0.8169
[rel]
12
10
8
6
4
2
0
6
5
4
3
2
1
[ppm]

t-Muurolol 3 1 "C:\Users\nmrsu\OneDrive - The Royal Botanic Gardens, Kew\Desktop\Eremophila NMR"

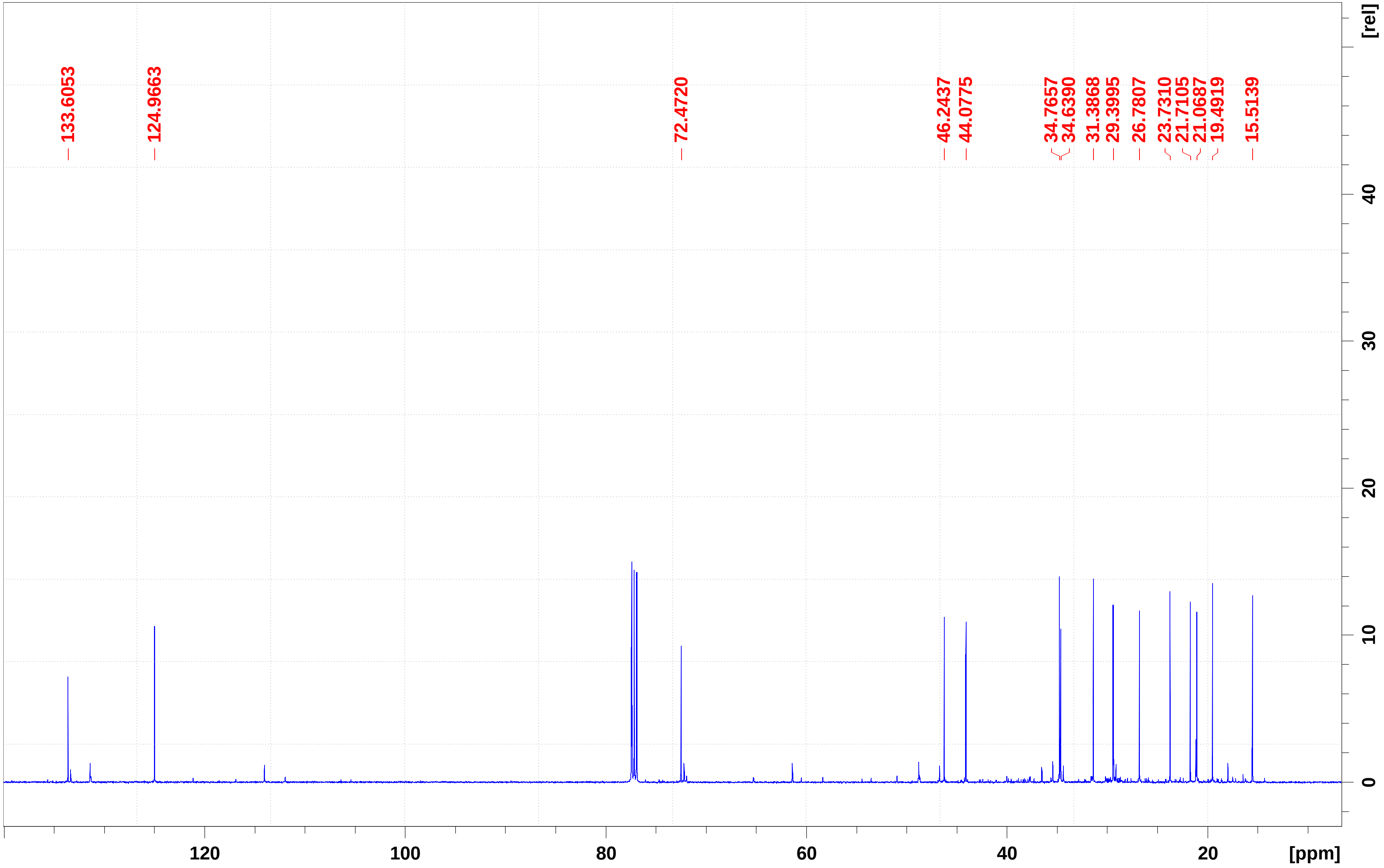

Supplement: Supplementary file 1 [file plants-10-00785-s001.zip › plants-1184483-supplementary.pdf]
